# Mapping the prevalence of cancer risk factors at the small area level in Australia

**DOI:** 10.1186/s12942-023-00352-5

**Published:** 2023-12-19

**Authors:** James Hogg, Jessica Cameron, Susanna Cramb, Peter Baade, Kerrie Mengersen

**Affiliations:** 1 Centre for Data Science, Queensland University of Technology (QUT), 2 George St, Brisbane City, Queensland 4000 Australia; 2 Viertel Cancer Research Centre, Cancer Council Queensland, 553 Gregory Terrace, Fortitude Valley, Queensland 4006 Australia; 3 Australian Centre for Health Services Innovation, School of Public Health and Social Work, Queensland University of Technology (QUT), 2 George St, Brisbane City, Queensland 4000 Australia

**Keywords:** Small area analysis, Cancer prevention, Disease mapping, Bayesian statistics

## Abstract

**Background:**

Cancer is a significant health issue globally and it is well known that cancer risk varies geographically. However in many countries there are no small area-level data on cancer risk factors with high resolution and complete reach, which hinders the development of targeted prevention strategies.

**Methods:**

Using Australia as a case study, the 2017–2018 National Health Survey was used to generate prevalence estimates for 2221 small areas across Australia for eight cancer risk factor measures covering smoking, alcohol, physical activity, diet and weight. Utilising a recently developed Bayesian two-stage small area estimation methodology, the model incorporated survey-only covariates, spatial smoothing and hierarchical modelling techniques, along with a vast array of small area-level auxiliary data, including census, remoteness, and socioeconomic data. The models borrowed strength from previously published cancer risk estimates provided by the Social Health Atlases of Australia. Estimates were internally and externally validated.

**Results:**

We illustrated that in 2017–2018 health behaviours across Australia exhibited more spatial disparities than previously realised by improving the reach and resolution of formerly published cancer risk factors. The derived estimates revealed higher prevalence of unhealthy behaviours in more remote areas, and areas of lower socioeconomic status; a trend that aligned well with previous work.

**Conclusions:**

Our study addresses the gaps in small area level cancer risk factor estimates in Australia. The new estimates provide improved spatial resolution and reach and will enable more targeted cancer prevention strategies at the small area level. Furthermore, by including the results in the next release of the Australian Cancer Atlas, which currently provides small area level estimates of cancer incidence and relative survival, this work will help to provide a more comprehensive picture of cancer in Australia by supporting policy makers, researchers, and the general public in understanding the spatial distribution of cancer risk factors. The methodology applied in this work is generalisable to other small area estimation applications and has been shown to perform well when the survey data are sparse.

**Supplementary Information:**

The online version contains supplementary material available at 10.1186/s12942-023-00352-5.

## Background

In 2020, an estimated 19.3 million people were diagnosed with cancer worldwide [1], causing a huge health burden. Moreover, incidence of cancer has been shown to exhibit strong spatial disparities, which due to improved models and better data accessibility are now communicated to the public via interactive Atlas platforms. In Australia, a notable Atlas is the Australian Cancer Atlas (ACA) [2], which provides interactive maps of small area level estimates of incidence and relative survival rates for a wide range of cancer types.

Whiteman et al. [3] suggest that at least one in every three cancers in Australia can be attributed to modifiable risk factors such as tobacco smoking, obesity, poor diet, insufficient physical activity, excessive sun exposure and alcohol consumption. Understanding the prevalence of cancer risk factors is pivotal to cancer prevention.

To better assess how cancer risk factors vary by location and target interventions, many countries have generated small area estimates for their prevalence including Australia [4], the US [5], Canada [6], Iran [7], and Luxembourg [8]. When generating small area estimates, practitioners must consider the *reach* and *resolution* of their results. Reach refers to the proportion of the small areas for which estimates are available, while resolution pertains to the small area population and geographical sizes. While the need for high resolution relates to minimizing outcome heterogeneity in larger areas and populations, the need for complete coverage (or high reach) ensures policy makers have complete spatial information. If small area estimates suffer from low reach or resolution the effectiveness of targeted interventions could be affected.

In Australia, the Social Health Atlases of Australia (SHAA) [4] is the major platform providing nationwide estimates for cancer risk factors at a small area level. The estimates were derived from the 2017–2018 National Health Survey (NHS). However the reach and resolution of the SHAA estimates could be improved. The larger areal units used in the SHAA combine heterogeneous sub-populations, resulting in estimates that are averages over different populations. The limitation regarding reach meant that no estimates are provided for very remote areas. Given that health disparities tend to widen with increasing remoteness [9–11], generating estimates for these areas is important for targeted public health initiatives in Australia. The modelled estimates provided by the SHAA use the best data source available, so the problem cannot be solved by using a different dataset or collecting better data; the solution is to use new methods of small area estimation (SAE) [12].

SAE is a well-established survey method that leverages auxiliary data, such as census data, to estimate parameters of interest for small geographic areas with limited or no survey data. Model-based SAE methods, which borrow strength across areas [13], can be applied at either the area [14] or individual level [15], with the latter requiring access to survey and census microdata.

Proportion area-level models are commonly used [16–19]; however, they become unsuitable when some of the input data (area-level proportion estimates) are unstable, i.e. exactly zero or one [20]. Sparse survey data and modelling rare or common population characteristics exacerbate this instability [21]. Solutions to instability include perturbing direct estimates prior to modelling [22] or excluding unstable areas [17]. Alternatively, modelling at the individual level, such as through multilevel regression and poststratification (MrP) [23], can be pursued. However, the use of individual level SAE models to derive proportion estimates are limited by the need for census microdata [24], which restricts the choice of covariates. Note that the modelling for the SHAA was conducted by the Australian Bureau of Statistics (ABS). Unfortunately, given that the published details of the ABS approach are modest [25], we can only infer the use of a individual level model.

While individual and area level models have limitations, recent work supports the utility of two-stage SAE approaches, which involve separate modelling at both levels [21, 26–28]. Two-stage approaches have many benefits that are particularly relevant for this application as they can alleviate unstable direct estimates by smoothing individual level outcomes, accommodate even severely sparse survey data thanks to multi-stage smoothing, and utilize more auxiliary data (e.g. survey-only covariates), permitting more flexible models and better predictions.

In this work, we generate small area level prevalence estimates for eight cancer risk factor measures using the Bayesian two-stage small area estimation methodology we developed for sparse survey data [21]. Our method considers a variety of data sources, including individual level survey data and area level auxiliary data such as census, remoteness and socioeconomic data. To assess the quality of our estimates, we used a dual validation approach whereby most SA2s are benchmarked to the sub-state level using fully Bayesian benchmarking [29], with the remaining SA2s (predominantly very remote areas) undergoing external validation. The results of this work will complement the current small area level estimates of cancer incidence and relative survival already available in the ACA [2].

## Data

### Geographical areas

Geographical location was defined according to the 2016 Australia Statistical Geography Standard (ASGS) [30]. We generated prevalence estimates at the statistical area level 2 (SA2) level, which is the lowest level of the ASGS hierarchy for which detailed census population characteristics are publicly available. SA2s are recognized as achieving the optimal balance between privacy and resolution [31]. Note that the SHAA provides estimates at a lower resolution, using population health areas (PHAs) which are constructed from single or multiple SA2s (40% and 39% of PHAs are constructed from one and two SA2s, respectively). In 2016 Australia had 1165 PHAs and 2310 SA2s [32] with median population sizes of 7500 and 15000 for SA2s and PHAs, respectively.

Throughout this analysis, we also used statistical area level 3 (SA3) and statistical area level 4 (SA4). By virtue of the hierarchical nature of the ASGS, SA2s are nested within SA3s, and SA3s are nested within SA4s. There is a median of 6 and 22 SA2s nested within each SA3 (n = 333) and SA4 (n = 88), respectively.

Of the 2310 SA2s covering Australia, SA2s with no physical location (comprising “Migratory-Offshore-Shipping” and “No usual address” codes for each State and Territory) (n = 18), very remote island SA2s (Christmas Island, Cocos Island, Norfolk Island and Lord Howe Island) (n = 4), and SA2s with annual average population $\le$ 10 (n = 67) were excluded. This left 2221 SA2s to use in the modelling. The remaining SA2s had a median (interquartile range (IQR)) population of 7859 (4483, 12753). Note that although Jervis Bay is classified as an “Other Territory” by the ABS, we included it as part of the state New South Wales.

### Data sources

#### Survey data

The individual level survey data and sampling weights were obtained from the 2017–18 National Health Survey (NHS), which is an Australia-wide population-level health survey conducted every 3–4 years by the ABS [33, 34]. This survey excluded very remote areas of Australia ($\approx 0.8$% of 2016 population), discrete Aboriginal and Torres Strait Islander communities ($\approx 0.5$% of 2006 population as per the ABS Community Housing and Infrastructure Needs Survey conducted only in 1999, 2001, and 2006 [35]), and non-private dwellings ($\approx 2$% of 2016 population [36]). Non-private dwellings include hotels and motels, hostels, boarding schools and boarding houses, hospitals, nursing and convalescent homes, prisons, reformatories and single quarters of military establishments and short-stay caravan parks. The ABS highlights that these exclusions should only have a minor effect on aggregate estimates for the states and territories of Australia.

**Fig. 1:**
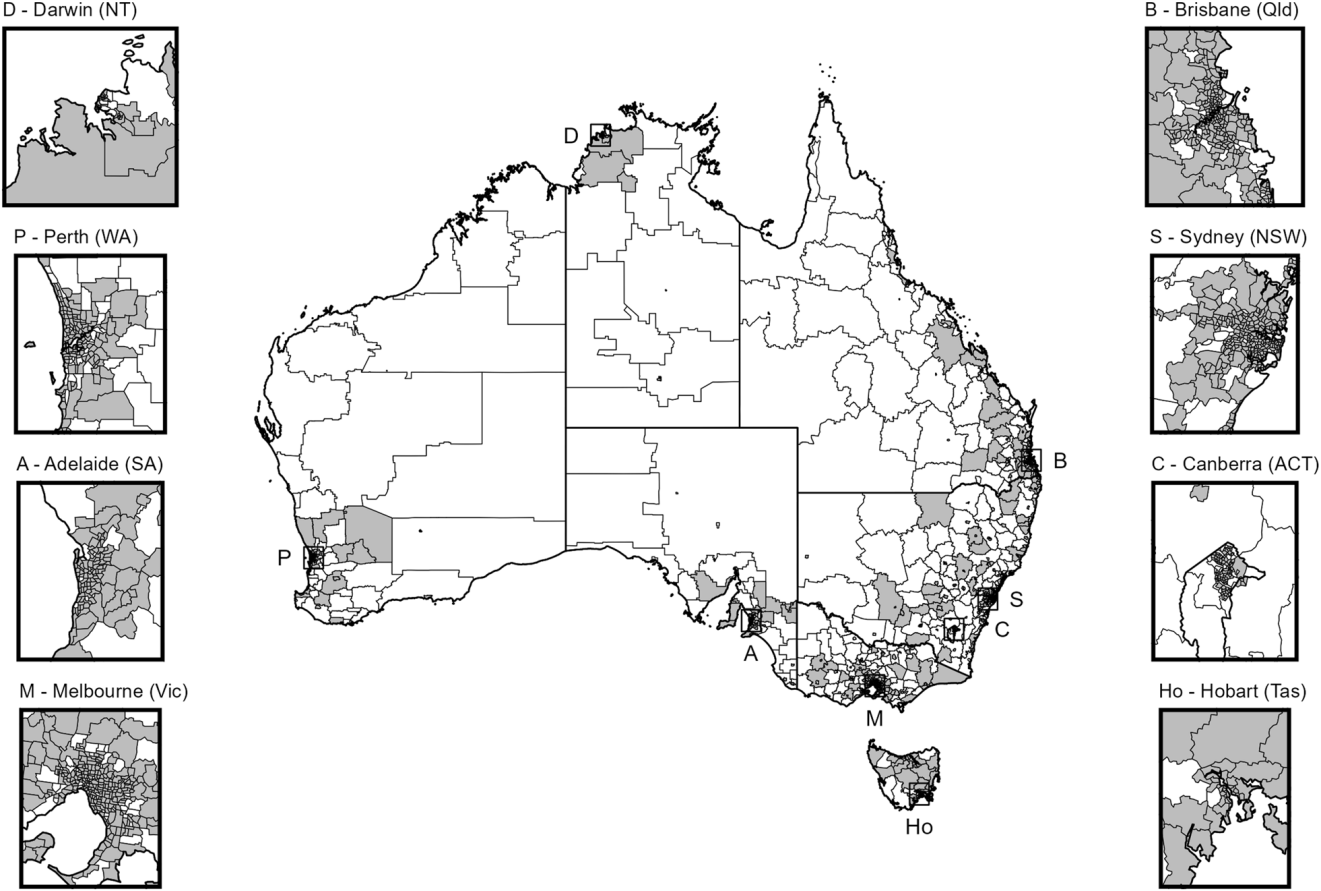
Map of 2221 SA2s in Australia with gray indicating an area with data from the 2017–2018 National Health Survey

The 2017–18 NHS data consist of 17248 sampled persons 15 years and older, with 878 persons under the age of 18. The data cover 1694 (76%) of the 2221 SA2s across Australia (see Fig. 1) and provide a median (IQR) SA2 level sample size of 8 (5, 13). The median SA3 and SA4 level sample sizes were 42 (25, 65) and 154 (101, 226), respectively. The NHS was also used to obtain daily smoking rates at the SA4 level [37]. Other sources of Australian health data are described in Section A of the Additional File 1.

#### Population data

Estimated Resident Population data stratified by 5-year age groups (15 years and above), sex and SA2, were obtained from the ABS for both 2017 and 2018 [38]. In this study the SA2 level population counts were derived by averaging across the two years. One of the risk factors (risky waist circumference) is only appropriate for ages 18+ and so modelling excluded persons under 18. Assuming that the single-year age distribution in this age group was uniform, we estimated that the population of 18–19-year olds was 40% of the 15–19-year old population.

For the SA2 level auxiliary data, we used data from the 2016 Australian census, represented as proportions (for categorical data) or averages (for continuous data) of individuals in each SA2. Census data for age, sex, non-school level education (higher education), highschool completion status, occupation, labour force status, personal weekly income, religious affiliation, registered marital status, First Nations Australian status, and household composition were obtained from the ABS [39]. These census factors made up 84 separate variables. Like Chidumwa et al. [40], to reduce the dimension of these socioeconomic and demographic data we used Principal Components (PC) Analysis, where we retained the first six principal components as they accounted for approximately 62% of the variation (see Section C.2 of the Additional File 1 for more details).

#### Other data

Australian research suggests that cancer burden [31, 41], and the prevalence of cancer risk factors varies strongly by remoteness and socioeconomic status (SES) [9, 42]. Data on SA1 level remoteness were provided by ABS and based on the Accessibility and Remoteness Index of Australia (ARIA+) [43], and converted to SA2 using population proportions. Remoteness is divided into five groups - major cities, inner regional, outer regional, remote, and very remote - based on a measure of relative geographic access to services. Given that very remote areas of Australia were intentionally excluded during data collection for the 2017–18 NHS, we followed the approach of Das et al. [37], and collapsed the outer regional, remote and very remote categories to a single remoteness group. Of the SA2s with sample data, 69% were major cities. The SA2 sample sizes tended to be larger for outer regional to very remote areas (median of 11 and IQR of 6 to 21) than for major city areas (median of 8 and IQR of 4 to 12).

SA2 level SES was sourced from the ABS Socio-Economic Indexes for Areas product [44]. Like other Australian health studies [37, 42, 45], we used the Index of Relative Socio-Economic Disadvantage (IRSD). The IRSD is a general SES index constructed using principal components analysis that summarises the economic and social conditions of individuals and households within a given area in order to determine the area’s overall relative disadvantage. A low IRSD score indicates a large proportion of relatively disadvantaged individuals in a given SA2 [45].

In this work, we used IRSD national deciles as a categorical variable with 10 groups, where 1 represents the most disadvantaged or lowest SES group and was used as the reference group. Although the IRSD can be used as a continuous variable, it is recommended to use deciles [44], and this also gave superior model performance. There were 44 of the 2221 SA2s without an IRSD value provided, so these had the closest IRSD decile assigned according to their corresponding PC1 (principal component 1) values.

We also obtained prevalence estimates and measures of uncertainty for risky alcohol consumption (more than 2 standard drinks a day on average), adequate fruit intake, obesity, overweight, current smokers and inadequate physical activity from the SHAA [4] at the Primary Health Network (PHN) and PHA level for adults. These data were downloaded as age-standardised rates per 100 people with 95% confidence intervals. Definitions and details are available in Section C and D of the Additional File 1 and the online SHAA platform [4].

### Risk factors

Broad risk factor groups were selected by consulting three sources: a wide range of experts in the fields of public health, epidemiology and oncology; literature, specifically evidence for casual associations [46] with, and population attributable fractions [3, 41, 47] for, cancer incidence; and the availability of data in the 2017–18 NHS. In this work we selected the following five broad risk factor groups: tobacco smoking, alcohol, diet, weight and physical activity. According to the 2015 Australian Burden of Disease study [41] these were attributable to 22.1%, 4.5%, 4.2%, 7.8% and 2.9% of the total cancer burden, respectively.

We explored a variety of possible measures and corresponding definitions for each of the five broad risk factor groups, placing priority on the definitions and recommendations used in the SHAA [4], the work by Whiteman et al. [3], Cancer Council Australia [48] and those provided by Australian government agencies such as Cancer Australia [45], the National Health and Medical Research Council (NHMRC), the Australian Institute of Health and Welfare (AIHW) [49] and the Australian Department of Health and Aged Care (DOH).

Table 1 summarizes the five broad risk factor groups and the eight corresponding measures and definitions. Table 2 gives direct estimates for these measures stratified by the eight states and territories of Australia. The risk factor measures proposed are designed to be cross-sectional and strike a natural balance between being specific to cancer while maintaining applicability to a variety of other health conditions [41]. Note that some risk factor groups, for example weight, required several differing measures.

**Table 1:** Descriptions and definitions of the five cancer risk factor groups and the measures within each. More details are given in Section B of the Additional File 1

Group	Measure	Measure definition
Smoking	Current smoking	Those who reported to be current smokers (including daily, weekly or less than weekly), and had smoked at least 100 cigarettes in their life.
Alcohol	Risky alcohol consumption	Those who exceeded the revised 2020 National Health and Medical Research Council (NHMRC) guidelines [50] of up to 10 standard drinks/week and no more than 4 standard drinks in any day. Compliance with the guidelines were assessed using self-reported alcohol consumption during the last three drinking days from the proceeding seven days.
Diet	Inadequate diet	Based on self-reported diet, those who did not meet both the fruit (2 serves/day) and vegetable (5 serves/day) 2013 NHMRC Australian Dietary guidelines [51].
Weight	Obese	Those with a measured BMI greater or equal to 30.
	Overweight/ obese	Those with a measured BMI greater or equal to 25.
	Risky waist circumference	Those with a measured waist circumference of $\ge$94cm (men) and $\ge$80cm (women). These cutoffs are only appropriate for adults, so we limited the survey dataset to all persons 18 years and older [4].
Physical activity	Inadequate activity (leisure)	Based on self-reported leisure physical activity, those who did not meet the 2014 Department of Health (DOH) Physical Activity guidelines [52], i.e. that each week adults (those between the ages of 18 and 64) should either do 2 1/2 to 5 hours of moderate-intensity physical activity or 1 1/4 to 2 1/2 hours of vigorous-intensity physical activity or an equivalent combination of both, plus muscle-strengthening activities at least 2 days each week. The DOH guidelines also provide specific recommendations for children (5 to 17 years), older persons (65 years and older) and pregnant women. In this work, the physical activity measures were derived from the ABS created variables that accommodated the guidelines across age groups.
	Inadequate activity (all)	Although similar to inadequate activity (leisure), this measures is based on workplace and leisure self-reported physical activity.

**Table 2:** Direct prevalence estimates for Australia and the eight states and territories for all eight cancer risk factor measures

	Current smoking	Risky alcohol consumption	Inadequate diet	Obese
Australia	15.1 (14.5, 15.7)	28.5 (27.8, 29.3)	46.9 (46.1, 47.7)	30.6 (29.9, 31.4)
New South Wales	14.4 (13.1, 15.7)	27.2 (25.5, 28.8)	45.4 (43.5, 47.2)	30.7 (29.0, 32.4)
Victoria	15.1 (13.6, 16.6)	26.0 (24.2, 27.8)	45.3 (43.3, 47.4)	30.9 (29.0, 32.8)
Queensland	15.8 (14.6, 17.1)	28.8 (27.2, 30.4)	47.0 (45.1, 48.8)	32.0 (30.4, 33.7)
South Australia	14.1 (12.3, 16.0)	28.0 (25.6, 30.3)	49.3 (46.8, 51.9)	31.6 (29.2, 33.9)
Western Australia	13.7 (11.9, 15.4)	31.3 (28.9, 33.7)	46.0 (43.4, 48.6)	28.0 (25.7, 30.3)
Tasmania	16.8 (14.9, 18.7)	29.2 (26.9, 31.6)	47.7 (45.1, 50.4)	33.7 (31.3, 36.2)
Northern Territory	20.5 (18.0, 23.0)	33.4 (30.4, 36.4)	48.4 (45.2, 51.5)	28.8 (26.0, 31.6)
Australian Capital Territory	11.2 (9.2, 13.2)	28.2 (25.3, 31.1)	49.6 (46.4, 52.7)	25.6 (22.9, 28.2)

	Overweight/obese	Risky waist circumference	Inadequate activity (leisure)	Inadequate activity (all)
Australia	65.7 (64.9, 66.4)	63.6 (62.8, 64.4)	85.2 (84.7, 85.8)	83.5 (82.9, 84.1)
New South Wales	64.9 (63.1, 66.6)	62.8 (61.0, 64.7)	84.8 (83.4, 86.1)	82.9 (81.5, 84.4)
Victoria	66.5 (64.6, 68.5)	64.1 (62.0, 66.1)	85.3 (83.8, 86.7)	83.4 (81.8, 84.9)
Queensland	65.0 (63.3, 66.7)	63.9 (62.1, 65.7)	87.0 (85.8, 88.3)	85.3 (84.1, 86.6)
South Australia	68.6 (66.1, 71.0)	66.0 (63.4, 68.6)	85.7 (83.8, 87.5)	84.3 (82.4, 86.2)
Western Australia	65.1 (62.6, 67.6)	62.0 (59.4, 64.7)	84.1 (82.2, 86.0)	82.2 (80.2, 84.2)
Tasmania	68.8 (66.4, 71.3)	68.5 (65.9, 71.0)	84.6 (82.6, 86.5)	83.1 (81.1, 85.1)
Northern Territory	63.1 (60.1, 66.2)	58.2 (55.0, 61.5)	86.2 (84.1, 88.4)	84.8 (82.5, 87.1)
Australian Capital Territory	62.5 (59.5, 65.6)	60.6 (57.4, 63.8)	82.1 (79.7, 84.6)	79.9 (77.4, 82.5)

Each cell of the table gives the direct prevalence estimate and corresponding 95% confidence interval as percentages

We defined the risk factor measures as binary where a survey individual received a value of one if they did not meet guidelines, or were in the unhealthy category. Unlike the SHAA which provides age-standardised rates by PHAs [4], we used proportions (prevalence) due to their common use in both the literature [12, 16, 53] and other digital Atlases [5]. Furthermore, deriving age-standardised rates requires prevalence estimates by area *and* age. This level of disaggregation is possible at the PHA level, but not feasible at the SA2 level.

We provide further details and the motivation for the selected risk factor measure definitions in Section B of the Additional File 1.

## Statistical models

### Bayesian model

Given the sparse nature of the available data for this SAE analysis, we used the Bayesian two-stage logistic normal (TSLN) approach we proposed recently [21]. Our previous study showed that the TSLN approach could outperform commonly used area [17, 18, 54] and individual level [55] models both in a simulation study focusing on sparse survey data and an application using the 2017–18 NHS data. The two-stage structure of the TSLN approach includes an individual level stage 1 model, followed by an area level stage 2 model.

The same TSLN approach, with very similar components, was chosen to be applied to all eight risk factor measures. The selection of fixed and random effect structures for the two models was guided by the goal of achieving a balance between parsimony across risk factor measures and predictive performance. We followed the advice by Goldstein [56] and initially used frequentist algorithms to select fixed and random effects, with fully Bayesian inference via Markov chain Monte Carlo (MCMC) for final model checking. Further details regarding model selection are given in Section E of the Additional File 1.

Let $y_{ij} \in \{0,1\}$ be the binary value from the NHS for sampled individual $j = 1, \dots , n_i$ in SA2 $i = 1, \dots , m$, where $n_i$ is the sample size in SA2 *i*. Further, let $m = 1694$ and $M = 2221$ be the number of sampled and total number of SA2s, respectively. The goal of this analysis is to generate estimates of the true proportions of each risk factor measure, $\varvec{\mu } = \left( \mu _1, \dots , \mu _M \right)$.

In this analysis, we used two versions of the survey weights, $w^{\text {raw}}_{ij}$, provided by the ABS [55, 57] to correct for sampling bias and promote design-consistency. The first, $w_{ij}$, was used for direct estimation and the second, $\tilde{w}_{ij}$, was used in the stage 1 model (see Section C.1 in the Additional File 1). Using the survey weights, small area proportion estimates can be computed using the Hajek [58] direct estimator,

1 $$\begin{aligned} \hat{\mu }^D_i = \frac{\sum _{j=1}^{n_i} w_{ij} y_{ij} }{n_i}, \end{aligned}$$

with an approximate sampling variance of [54, 59],

2 $$\begin{aligned}{} & {} \psi _i^D = \widehat{\text {v}} \left( \hat{\mu }_i^D \right) = \frac{1}{n_i} \left( 1 - \frac{n_i}{N_i} \right) \left( \frac{1}{n_i - 1} \right) \nonumber \\{} & {} \quad \sum _{j=1}^{n_i} \left( w_{ij}^2 \left( y_{ij} - \hat{\mu }_i^D \right) ^2 \right) . \end{aligned}$$

Direct estimators, such as Eqs. (1) and (2), have low variance and are design-unbiased for $\mu _i$ when $n_i$ is large, but have high variance when $n_i$ is small [13].

#### Stage 1: Individual level model

**Fig. 2:**
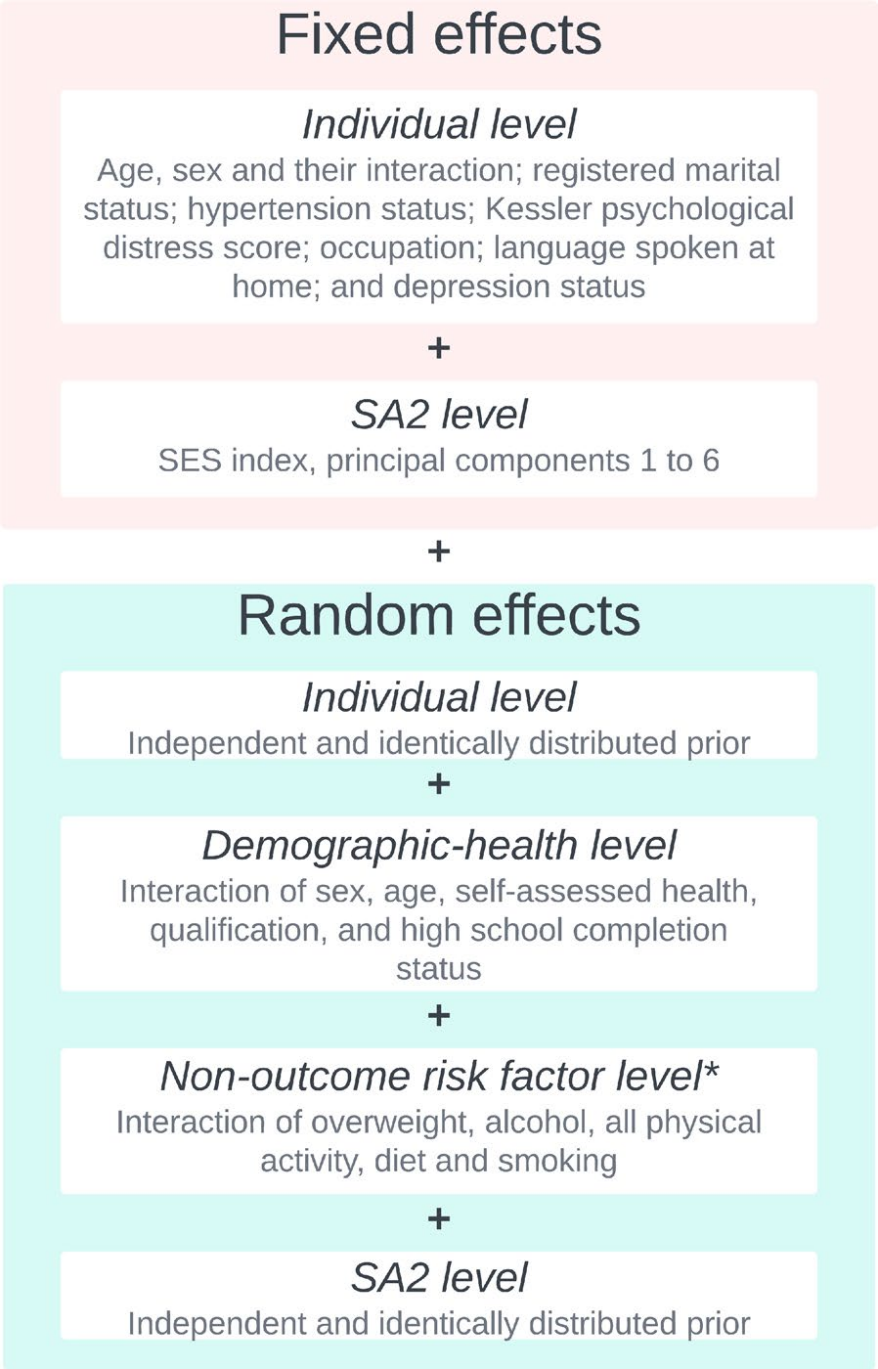
Schematic describing the components of the linear predictor for $\text {logit}\left( \pi _{ij} \right)$ in the stage 1 model. *The non-outcome risk factor categorical covariate was derived from the interaction of the binary risk factor outcomes not directly associated with the risk factor being modelled. For more details see Section C of the Additional File 1. *SA2* Statistical area level 2

The stage 1 model is a Bayesian pseudo-likelihood logistic mixed model. Let $\pi _{ij}$ be the probability of $y_{ij} = 1$ for sampled individual *j* in SA2 *i*. Following the notation of Parker et al. [55], we represent the pseudo-likelihood for a probability density, *p*(.), as $p\left( y_{ij} \right) ^{\tilde{w}_{ij}}$. Pseudo-likelihood is used to ensure the predictions from the logistic model are approximately unbiased under the sample design [60, 61]. Thus, the stage 1 model likelihood is given by,

3 $$\begin{aligned} y_{ij} \sim \text {Bernoulli}\left( \pi _{ij} \right) ^{\tilde{w}_{ij}}, \end{aligned}$$

where $\text {logit}\left( \pi _{ij} \right)$ is modelled using a generic linear predictor that is application-specific. In this work, we used several unique components summarised in Fig. 2. The linear predictor included eight individual level categorical covariates and seven area level covariates as fixed effects. Unstructured individual and SA2 level random effects were also applied. In addition to these, borrowing ideas from MrP [62], we included two hierarchical random effects based on categorical covariates that were themselves derived from the interaction of numerous individual level demographic and health covariates. A discussion of the priors used is given on the subsequent page. More details can be found in Section C of the Additional File 1.

#### Stage 2: Area level model

After fitting the stage 1 model, the individual level predictions are aggregated to the area level, producing stage 1 (S1) proportion estimates $\hat{\mu }^{\text {S1}, (t)}_i$ using Eq. (1), and sampling variances, $\psi ^{\text {S1}, (t)}_i = \widehat{\text {v}} \left( \hat{\mu }_i^D \right) + \widehat{\text {v}} \left( \hat{B}^{(t)}_i \right)$, for all posterior MCMC draws, $t=1, \dots , T$ [21], where the function to compute the sampling variance, $\widehat{\text {v}}(.)$, is given in Eq. (2) and $\hat{B}^{(t)}_i = n_i^{-1} \left( \sum _{j=1}^{n_i} w_{ij} \left( \pi ^{(t)}_{ij} - y_{ij} \right) \right)$ quantifies the level of smoothing achieved by using $\pi _{ij}$ instead of $y_{ij}$.

Using the common logistic transformation [18, 54], let

4 $$\begin{aligned} \hat{\theta }_i^{\text {S1}, (t)}= & {} \text {logit}\left( \hat{\mu }_i^{\text {S1}, (t)} \right) \end{aligned}$$

5 $$\begin{aligned} \tau _i^{\text {S1}, (t)}= & {} \psi _i^{\text {S1}, (t)} \left[ \hat{\mu }_i^{\text {S1}, (t)} \left( 1 - \hat{\mu }_i^{\text {S1}, (t)} \right) \right] ^{-2}, \end{aligned}$$

thereby permitting the use of a Gaussian likelihood in the second stage model. Let $\bar{\tau }_i^{\text {S1}}$ be the empirical posterior mean of $\tau _i^{\text {S1}}$ and $\widehat{\text {v}} \left( \hat{\theta }_i^{\text {S1}} \right)$ be the empirical posterior variance of $\hat{\theta }_i^{\text {S1}}$. Finally, by selecting a random subset of the posterior draws, say $\widetilde{T}$, let $\hat{\varvec{\theta }}^{\text {S1}}_i = \left( \hat{\theta }^{\text {S1}, (1)}_i, \dots , \hat{\theta }^{\text {S1}, (\widetilde{T})}_i \right)$.

The stage 2 model is a Bayesian spatial Fay-Herriot [14] model. Unlike previous two-stage approaches [26, 27], we accommodate some of the uncertainty inherent in fitting the stage 1 model by using the vector $\hat{\varvec{\theta }}^{\text {S1}}_i$ as input to the stage 2 model. The stage 2 model likelihood for the posterior draws from the stage 1 model is,

6 $$\begin{aligned} \hat{\varvec{\theta }}_i^{\text {S1}} \sim \text {N}\left( \theta _i , \bar{\tau }_i^{\text {S1}} + \widehat{\text {v}} \left( \hat{\theta }_i^{\text {S1}} \right) \right) \end{aligned}$$

where $\theta _i$ is modelled using a generic linear predictor that is problem specific. The final proportion/prevalence estimate for the *i*th SA2, denoted $\mu _i$, is given by the posterior distribution of $\text {logit}^{-1} \left( \theta _i \right)$. To ensure that posterior uncertainty remains unaffected by the choice of $\widetilde{T}$, we downscale the likelihood contribution by $1/\widetilde{T}$.

**Fig. 3:**
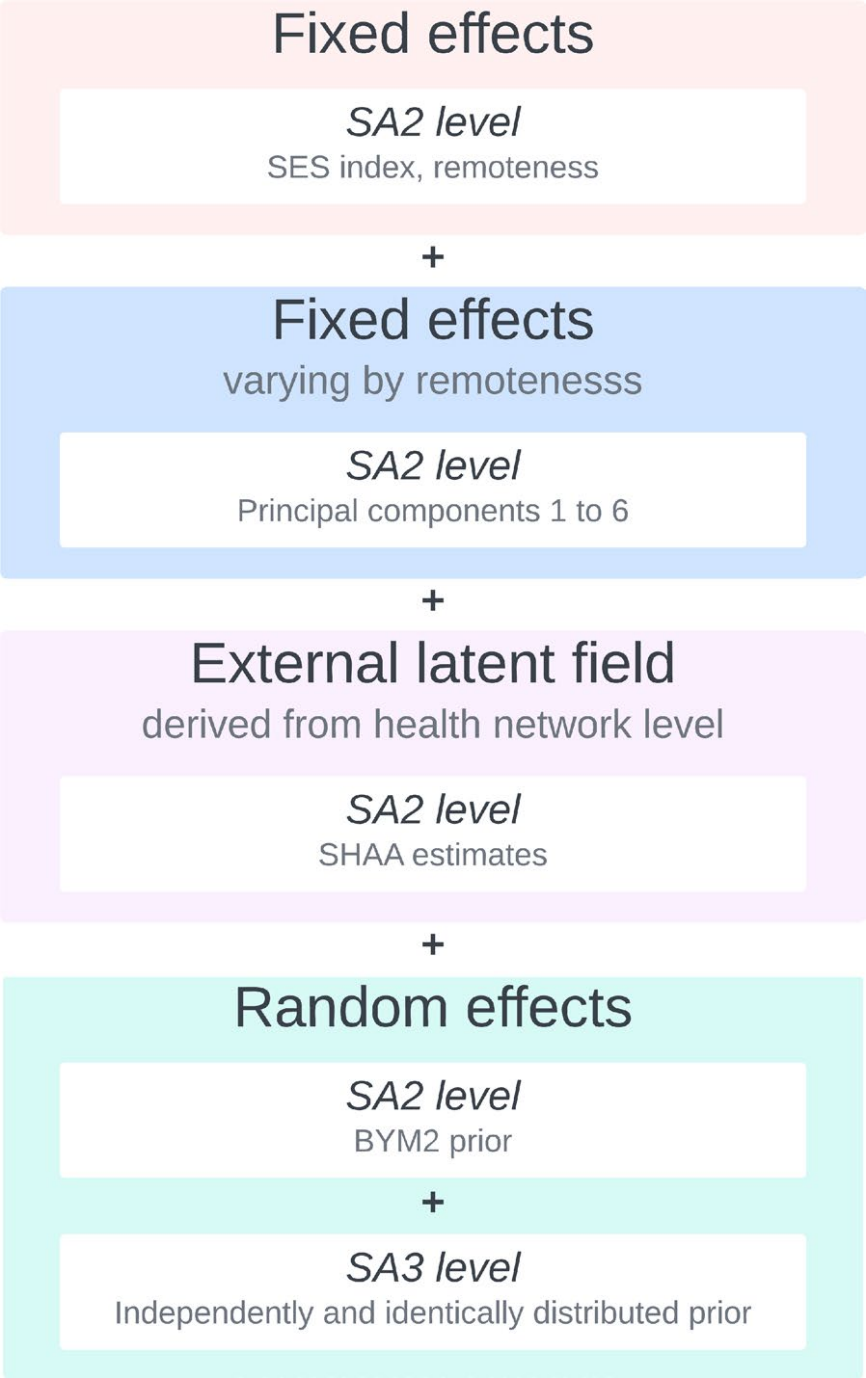
Schematic describing the components of the linear predictor for $\theta _i$ in the stage 2 model. For more details see Section C of the Additional File 1. SA2: Statistical area level 2; SA3: Statistical area level 3; SHAA: Social Health Atlases of Australia

In this work, we used several unique components for the linear predictor of $\theta _i$ which are summarised in Fig. 3. The linear predictor included the SES index deciles and remoteness as standard fixed effects. In addition, PC1 to PC6 were used as fixed effects with coefficients varying according to remoteness. The linear predictor also included an external latent field constructed from the SHAA’s estimates and a BYM2 spatial random effect [63] at the SA2 level. Given we did not include SA3 level census covariates, an unstructured random effect at the SA3 level was employed. To smooth unstable variances we used the generalized variance function [12, 64, 65] described in Section C.4.6 of the Additional File 1. More details can be found in Section C of the Additional File 1.

### Priors

The Bayesian models described above are completed by the specification of priors. Given the complexity of the two models, in this work generic weakly informative priors were adopted based on preliminary analysis of the data [66]. In both models, all fixed effect coefficients were given $\text {N}\left( 0, 2^2 \right)$ priors with intercepts given a student-$t\left( 0, 2^2, \text {df} = 3 \right)$. We used $\text {N}^{+}\left( 0, 1^2 \right)$ and $\text {N}^{+}\left( 0, 2^2 \right)$ priors for all standard deviation terms in the stage 1 and stage 2 models, respectively. The mixing parameter in the BYM2 [63] random effect was given a $\text {Uniform}\left( 0,1 \right)$ prior (see Section C of the Additional File 1).

We conducted sensitivity analysis by using more, $\text {N}\left( 0, 1^2 \right)$, and less, $\text {N}\left( 0, 100^2 \right)$, informative priors for the fixed effects in both models. We also experimented with using exponential priors with rates of 0.5 and 1 for standard deviation terms. Finally, we examined model fit when using an informative Beta prior for the mixing parameter. We found that the model fit and prevalence estimates were unaffected by these prior changes. The chosen priors gave superior sampling efficiency and convergence.

### Validation

For validation of the small area estimates, we adopted a dual approach, using both internal and external methods. See Section C.5 in the Additional File 1 for details.

#### Internal benchmarking

Internal validation involved a fully Bayesian benchmarking procedure [29] that adjusts the results obtained in the stage 2 model by penalizing discrepancies between modelled and direct estimates. Unlike previous benchmarking approaches that adjust the point estimates only [13, 67], Bayesian benchmarking adjusts the entire posterior — automatically accounting for benchmarking-induced uncertainty.

In this work we simultaneously enforced two benchmarks referred to as “state” and “major-by-state”. The state benchmark had seven groups which were composed of the states and territories of Australia (except the Northern Territory, which was not benchmarked due to ABS instruction [57]).

The major-by-state benchmark had 12 groups, composed of the interaction of the states and territories of Australia (except the Northern Territory) and dichotomous remoteness (major city vs non-major city). Thus, for each state, apart from Tasmania (where all areas were non-major city), and the Australian Capital Territory (where 96% of areas were major city), each SA2 was benchmarked differently depending on whether the area was in a major city or not.

#### External validation

External validation was performed by comparing the estimates to those from the SHAA at the PHA level and the overall trends observed in the modelled results with the general findings from other Australian health surveys conducted on specific sub-populations, such as states [68] or First Nations Australians [69]. Although this validation affirmed the validity and reliability of our estimates in general, it was particularly helpful in assessing the credibility of estimates for areas that could not be benchmarked.

### Computation

We used fully Bayesian inference using MCMC via the R package rstan Version 2.26.11 [70]. Where possible we used the non-mean centered parameterization for random effects and the QR decomposition for fixed effects [71]. The stan code for the stage 1 and stage 2 models can be found on GitHub [72].

For the stage 1 model we used 1000 warmup and 1000 post-warmup draws for each of the four chains, feeding a random subset of 500 posterior draws from the stage 1 to the stage 2 model. For the stage 2 model we used 3000 warmup and 3000 post-warmup draws for each of four chains. For storage reasons we thinned the final posterior draws from the stage 2 model by 2, resulting in 6000 useable posterior draws.

Convergence of the models was assessed using trace and autocorrelation plots, effective sample size and $\hat{R}$ [73]. While convergence ranged slightly between risk factors, all the proportion parameters, $\varvec{\mu } = \left( \mu _1, \dots , \mu _M \right)$, had $\hat{R} < 1.03$, with 96% having effective sample sizes $>1000$ and 99% having $\hat{R} < 1.01$.

### Summaries and visualisation

Estimates from the benchmarked stage 2 model were reported in a variety of forms, including absolute, relative and classification measures. For point estimates we used posterior medians and for uncertainty intervals we used 95% highest posterior density intervals (HPDIs). We used the modelled proportions as the absolute indicator and odds ratios (ORs) as the relative indicator. The ORs for the *t*th posterior draw were derived as,

7 $$\begin{aligned} \text {OR}^{(t)}_i=\, & {} \frac{\mu ^{(t)}_i/(1-\mu ^{(t)}_i)}{\hat{\mu }^D/(1-\hat{\mu }^D)} \end{aligned}$$

with $\hat{\mu }^D$ being the national prevalence estimate for the risk factor measure. An OR above one indicates that the SA2 has a prevalence higher than the national average.

In addition to using point estimates and credible intervals to summarize the ORs, we also used the exceedence probability (EP) [31, 53, 74].

8 $$\begin{aligned} EP_i = \frac{1}{T} \sum _t \mathbb {I} \left( \text {OR}^{(t)}_i > 1 \right) \end{aligned}$$

Generally an EP above 0.8 (or below 0.2) is considered to provide evidence that the proportion in the corresponding SA2 was substantially higher (or lower) than the national average, respectively [75]. Note that the exceedance probabilities calculated using either ORs or prevalence are identical.

To facilitate decision-making, we classified SA2s by assessing whether their individual and neighbor values (i.e. clusters [76, 77]) were significantly different to the national average. In this work, these classifications were called *evidence classifications*. Any area classified as HC, H, L, or LC has an exceedance probability suggesting that the modelled prevalence is significantly different to the national average; HC or H denotes higher, while L or LC denotes lower. The difference between HC and H (or LC and L) is that the former provides an indication of clustering of areas, while the later only indicates significance of the area itself. If an area is not classified according to the criteria above (defined as None (“N”)) the modelled estimate is not sufficiently different to the national average. See details in Section D.3 of the Additional File 1.

Code to produce subsequent plots is available on GitHub [72].

## Results

### Prevalence

**Fig. 4:**
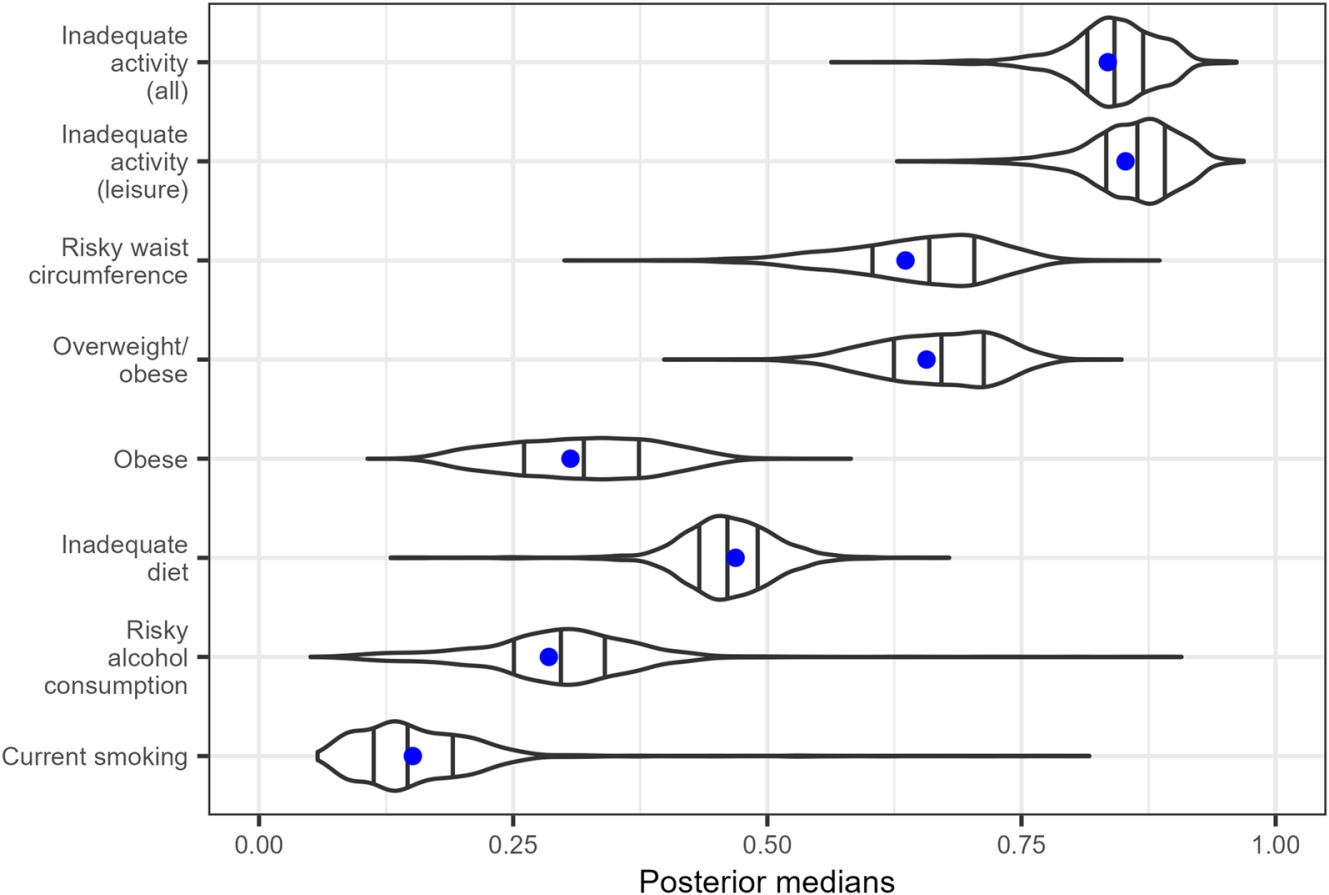
Violin plots describing the distribution of the posterior medians of the proportion estimates for each of the eight cancer risk factor measures. The width of each curve corresponds to the approximate frequency of the posterior medians similar to a density plot. The three vertical lines within the violins denotes the 25th, 50th and 75th quantiles of the posterior medians. The tails of each violin extend to the minimum and maximum values. The blue dots represent the nationwide direct estimates

**Fig. 5:**
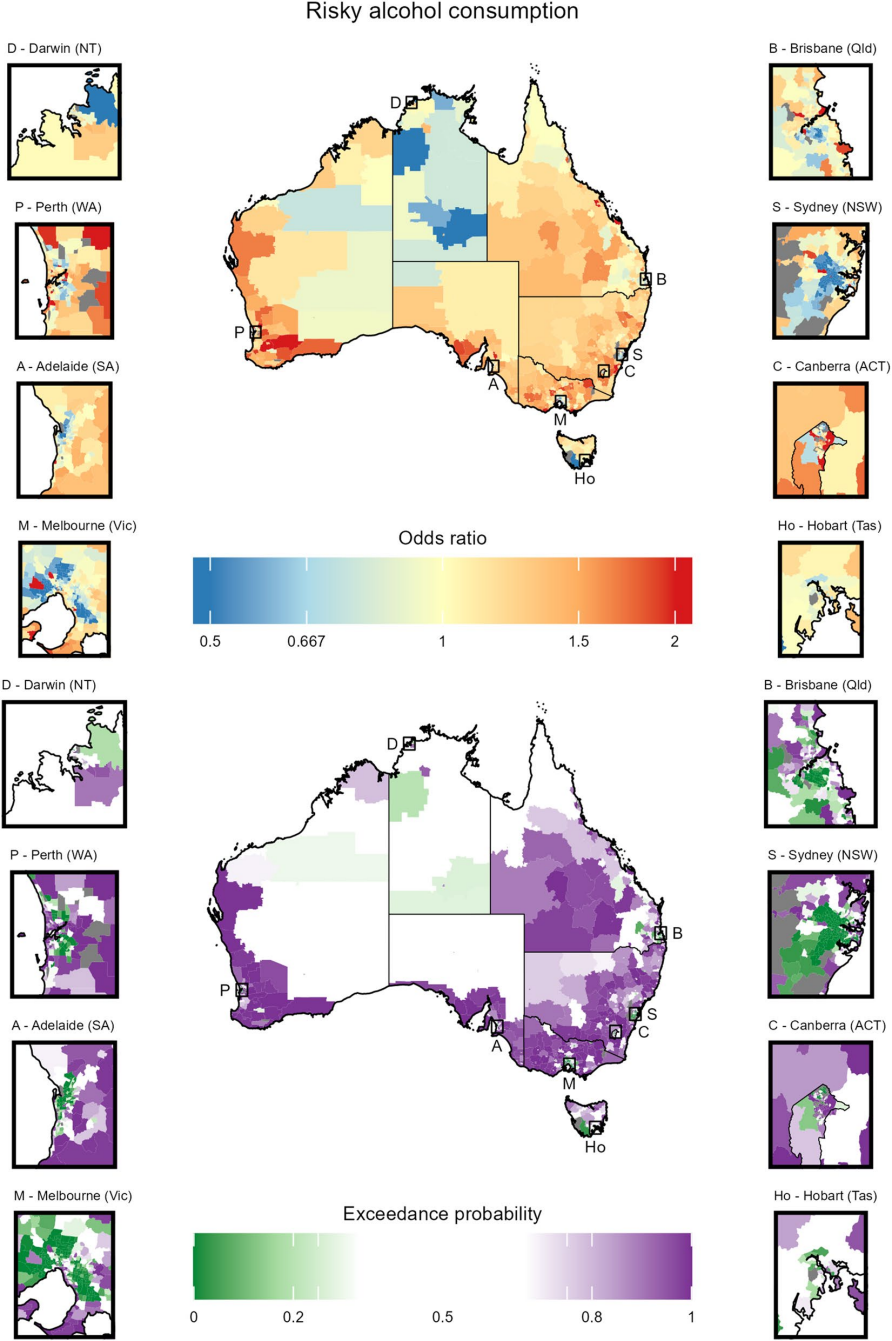
Choropleth maps displaying the results for risky alcohol consumption (see Table 1) for 2221 SA2s across Australia. The top plot gives the posterior median of the odds ratios (OR). ORs above 1 indicate that the prevalence is higher than the national average. The bottom plot gives the exceedance probabilities (EPs) for the ORs. The map includes insets for the eight capital cities for each state and territory, with black boxes on the main map indicating the location of the inset. Note that some values are lower (or higher) than the range of color scales shown; for these values, the lowest (or highest) color is shown. Grey areas were excluded from estimation due to the exclusion criteria. Black lines represent the boundaries of the eight states and territories of Australia

**Fig. 6:**
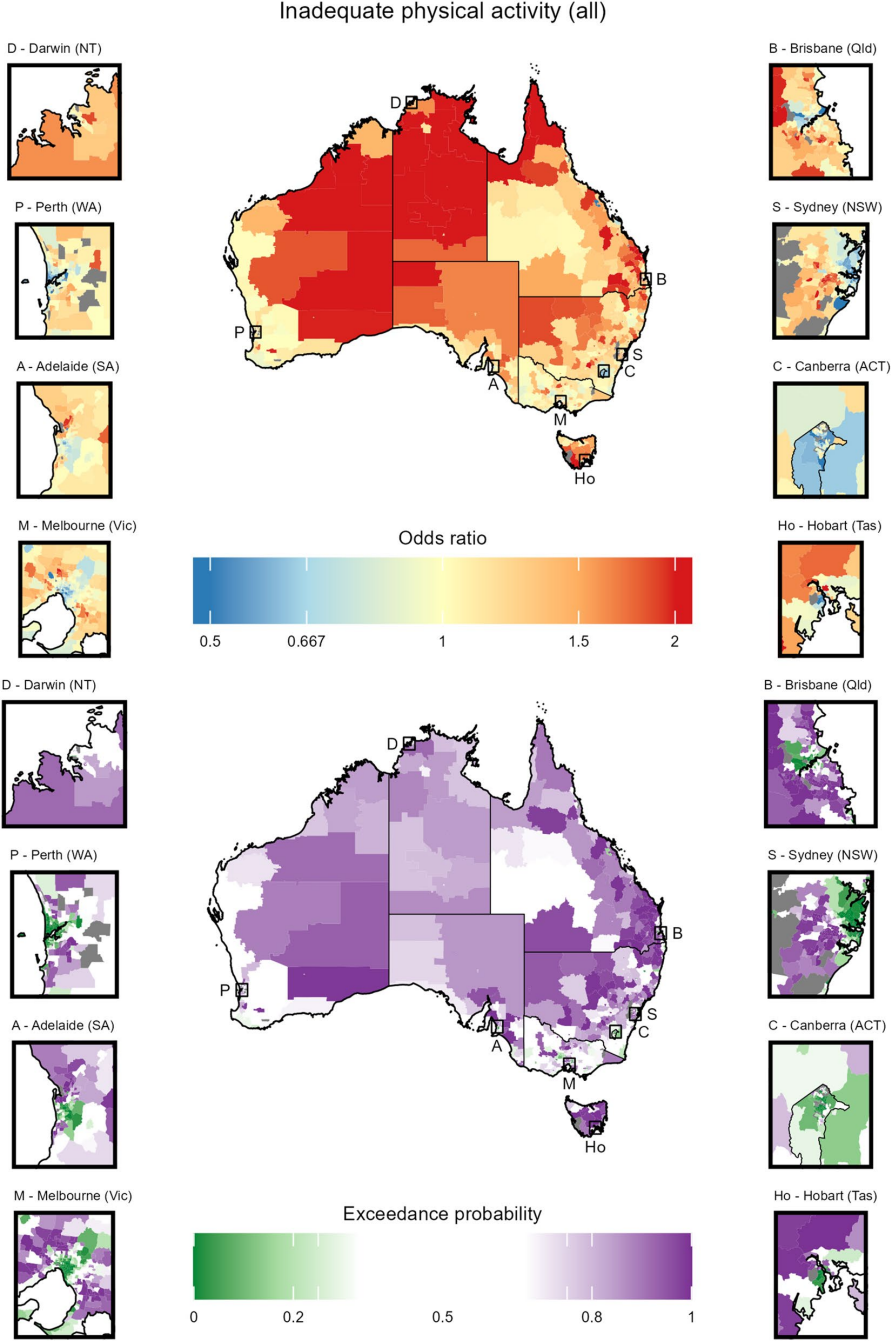
Choropleth maps displaying the results for inadequate physical activity (all) (see Table 1). For more details see the caption for Fig. 5

Large spatial variation in the proportion of cancer risk factors across Australia can be clearly observed in Figs. 4,5,6 and Section H of the Additional File 1. Slightly more heterogeneity of the point estimates was observed within major cities as a result of the much greater socioeconomic variation within these areas. For example, the range of principal component 1 (a proxy for SES that is unique to the SES index) was largest in major cities and inner regional areas, but 50% the size in remote and very remote areas.

**Fig. 7:**
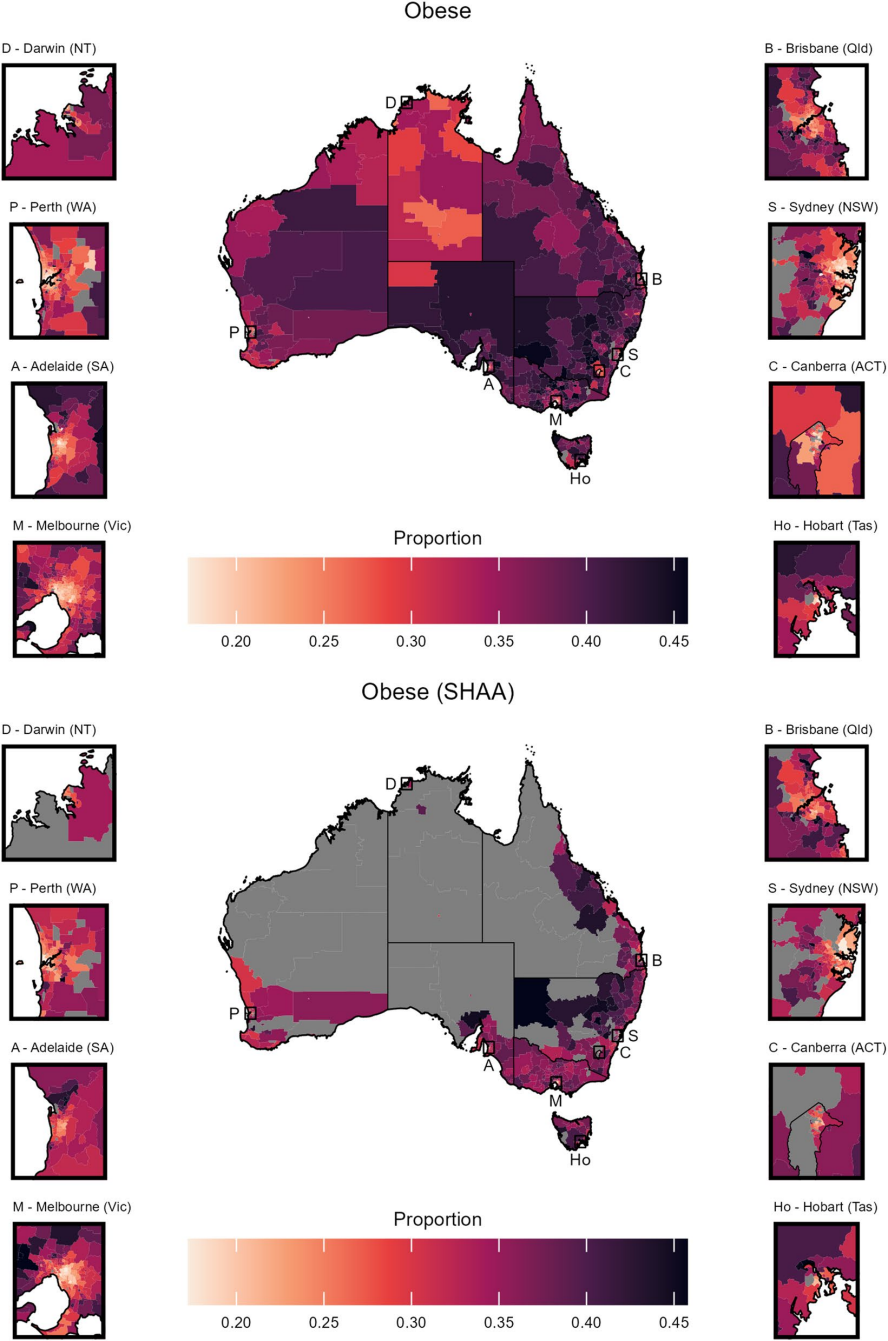
Choropleth maps of obesity prevalence at the (top) SA2 level from this work and (bottom) PHA level from the SHAA platform [4]. The maps include insets for the eight capital cities in each state and territory, with black boxes indicating their location. Note that some values are lower (or higher) than the range of color scales shown; for these values, the lowest (or highest) color is shown. Grey areas represent no estimates, and black lines denote state and territory boundaries. Our estimates and SHAA’s use similar but not identical definitions, with our values reported as proportions and SHAA’s as age-standardized rates converted to proportions for comparison

Stratifying by risk factor, the results highlight interesting patterns and trends. A more thorough discussion of the result is given in Section F of the Additional File 1.

- Current smoking (Section H.2 in the Additional File 1): Spatial patterns show lower prevalence in major cities and less disadvantaged areas. Although very high prevalence estimates are observed in the very remote regions in the middle of the country, these estimates come with substantial uncertainty.
- Risky alcohol consumption (Section H.3 in the Additional File 1): The spatial patterns were inconsistent with the other factors, particularly in terms of the relationship between (higher) socioeconomic status and healthy behaviours. The results suggest that less disadvantaged areas have higher prevalence, which generally manifests in higher prevalence in major cities. Unlike other risk factors where prevalence estimates exhibit relative homogeneity within the SES index deciles and remoteness groups (see Section G of the Additional File 1), for risky alcohol consumption the estimates exhibit far greater heterogeneity for more disadvantaged areas in major cities.
- Inadequate diet (Section H.4 in the Additional File 1): The spatial patterns suggest less dependence on the SES index and remoteness than the other risk factors. Inadequate diet exhibits the lowest heterogeneity of the risk factors considered in this work.
- Body weight (Sections H.5 to H.7 in the Additional File 1): Similar spatial patterns are observed for the three measures. The prevalence was very strongly tied to remoteness with substantially lower prevalence almost exclusively occurring in major cities. Furthermore, the most notable differences in patterns between the estimates for obese and overweight/obese are found in major cities.
- Physical activity (Sections H.8 to H.9 in the Additional File 1): Similar spatial patterns are observed for the two measures. Lower prevalence of inadequate activity is observed in major cities and less disadvantaged areas.

The estimates demonstrate reliability, as around 97% of them possess coefficients of variation (CV) below 25% — a widely accepted threshold for reliability [25]. Furthermore, the modelled estimates show considerable stability improvements over the SA2 direct estimates with a reduction in variability (measured by standard deviation) across Australia by an average factor of 3.3. The estimate uncertainty varied by risk factor, with current smoking having the highest median CV (17.4) and inadequate activity (leisure) having the lowest (2.6). The distribution of CV also varied by remoteness; the median CV for major cities (62% of the survey data) was, on average, 1.8 to 3.1 times smaller than that for very remote areas.

To investigate the impact of the finer resolution, we derived PHA level CVs by taking the population weighted mean of the SA2 level estimates. The CVs of point estimates at the SA2 level range from 5% to 34% larger than point estimates at the PHA level across the risk factors. Similarly, by calculating and summarising the heterogeneity of SA2s within each PHA, we find that across the risk factors, the median PHA CV is between 1.5% to 9.1%. Of the PHAs composed of multiple SA2s, 10% have CVs $>15$%. The large CVs indicate that the corresponding PHAs were highly heterogeneous, highlighting the benefits of using higher resolution estimates. Given the similar definitions for the obese risk factor measure, Fig. 7 compares the estimates used in this work and that of the SHAA, indicating strong agreement.

Section G of the Additional File 1 provides more plots describing the modelled results, including how they vary by the SES index and remoteness. An interactive exploration of the modelled results will be made available in the Australian Cancer Atlas 2.0 [2], planned for release in early 2024.

### Evidence classifications

**Fig. 8:**
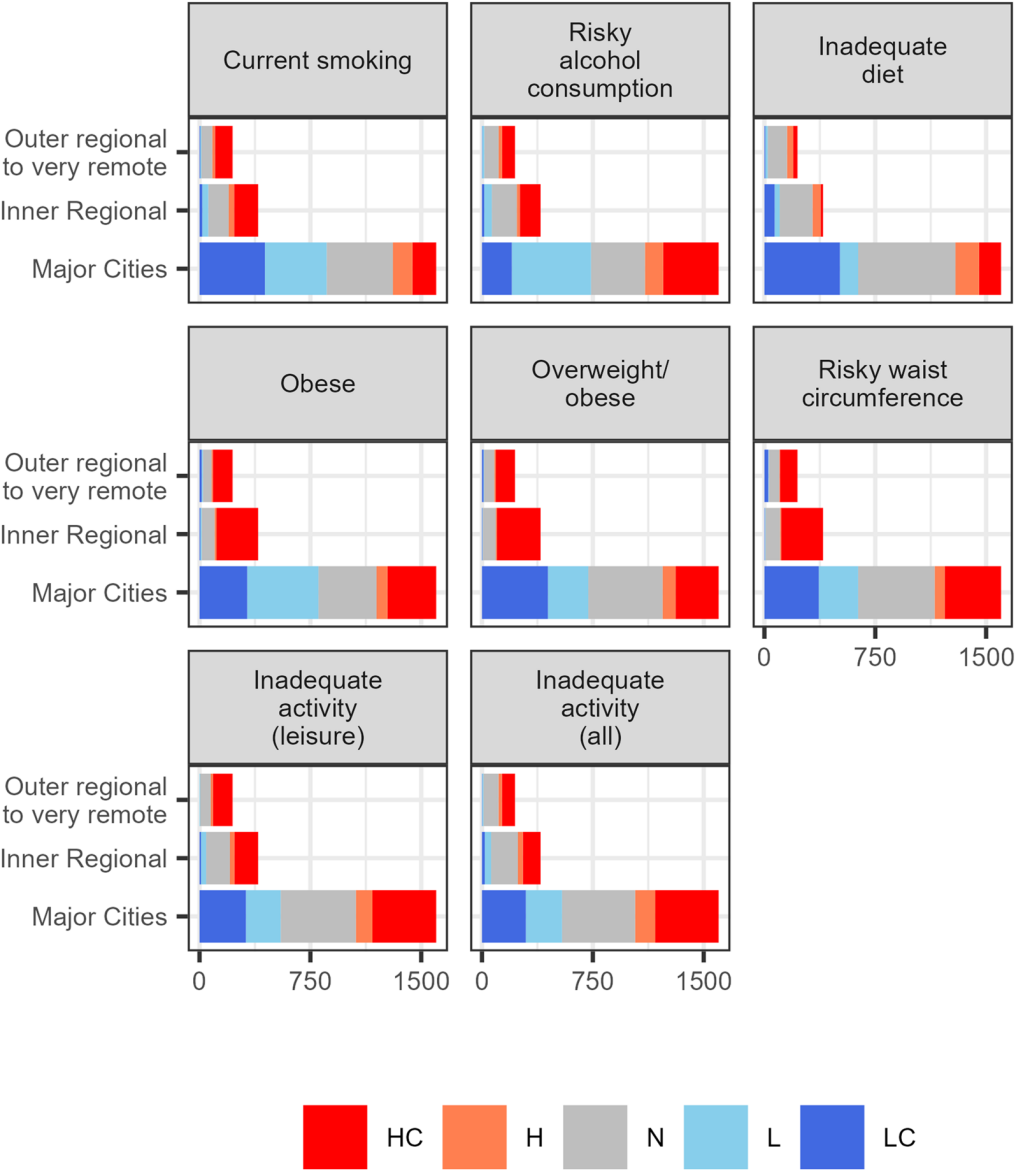
Distribution of the evidence classifications (HC, H, N, LC, and L) by remoteness and risk factor. The *x*-axis is the weighted number of SA2s using the 2017–2018 ERP as weights

Table 3 summarises the number of evidence classifications for each risk factor measure. Figure 8 stratifies these by remoteness. A similar stratified plot for the SES index is found in Section G of the Additional File 1.

**Table 3:** Distribution of evidence classifications by risk factor measure (excluding “N” category)

	Total	HC	H	L	LC
Current smoking	1469	442	154	408	466
Risky alcohol consumption	1482	603	137	523	219
Inadequate diet	1155	194	221	155	586
Obese	1663	745	89	487	342
Overweight/obese	1539	718	94	267	460
Risky waist circumference	1523	783	76	267	397
Inadequate activity (leisure)	1458	724	148	262	324
Inadequate activity (all)	1411	637	177	278	318

The values in the table are population weighted counts

Across most risk factors many more HC areas are identified than LC areas. For example, for risky waist circumference, around 783 SA2s are classed as HC, while only around 397 are classed as LC. We observed that HC or H evidence classifications are generally found in the most disadvantaged areas, while L or LC areas are more likely in the least disadvantaged areas in major cities.

The evidence classifications revealed several interesting trends. For the physical activity risk factor measures, a larger proportion of the areas in major cities were classified as HC or H as opposed to LC or L. For inadequate physical activity, the HC classifications favour the most disadvantaged areas. The weight risk factor measures exhibited different trends with a relatively even distribution of evidence classifications in major cities. Furthermore, almost all areas classified as LC or L occurred in less disadvantaged areas. As mirrored in the maps, the evidence classifications for smoking suggest a very strong correlation with remoteness and SES; almost all the LC or L classifications occur in major cities and less disadvantaged areas. Inadequate diet has the smallest number of evidence classifications (1155 out of 2221), with the largest proportion of them being LC areas in major cities and less disadvantaged areas. The results for risky alcohol consumption suggest that less disadvantaged areas have higher proportions of risky alcohol consumption; a trend unique to this risk factor measure.

## Discussion

This work improves the spatial resolution and reach of previously published cancer risk factor estimates in Australia. While the estimates highlight broadly similar findings as those from the SHAA, they provide greater resolution and reach allowing for more granular exploration of the spatial disparities (see Fig. 7). This is particularly pertinent due to the heterogeneity of the component SA2s within each PHA in terms of population size, socioeconomic status and remoteness.

By improving the reach of the previously published cancer risk factor estimates, the estimates in this work uniquely enable the exploration of spatial disparities in very remote areas of Australia. As expected, the very remote areas have far greater uncertainty than those in major cities (CVs greater than 3 times higher). Nevertheless, by utilising the estimates and their uncertainty measures policy makers will have the capability to more effectively allocate health interventions and resources to these disadvantaged areas and triage areas where more data should be collected in the future to improve the quality of small area estimates.

The cancer risk factor estimates generated in this work reveal substantial spatial disparities in cancer risk behaviours across Australia, with higher prevalence of high risk behaviours generally occurring in more remote areas. While the prevalence of most cancer risk factors is higher in areas of lower SES, the spatial patterns for risky alcohol consumption demonstrated the opposite effect. Point estimates for risky alcohol consumption and current smoking exhibited the most heterogeneity across Australia, while those from the physical activity measures exhibit the least. The distribution of the point estimates are mostly consistent across states and territories of Australia.

Although generating prevalence estimates and their uncertainty intervals are useful in a variety of applications, using them to visualize which areas are substantially different to the national average can be difficult as the two components must be considered jointly. By further classifying the estimates according to their posterior probabilities, we were able to streamline this process. Classifications, such as those used in this work, are pivotal in developing targeted interventions as they enable policymakers to quickly identify areas, or groups of areas, with substantially higher (or lower) prevalence.

Our Bayesian methodology, along with its associated exceedance probabilities and evidence classifications, provides insights that cannot easily be attained via the estimates from the SHAA. Although the spatial patterns of the evidence classifications vary by risk factor, a consistent pattern was that areas with lower than average prevalence of risk factors (classified as LC or L) were almost exclusively located in major cities. Although there were areas with higher than average prevalence (HC or H) in major cities these were often less common, except for the physical activity risk factors where about half were higher and lower than the national prevalence.

Although this applied work represents a significant step in the ongoing improvements in cancer prevention in Australia, it has some limitations. Firstly and most critically, like previous research [47, 78], most of the risk factor measures used were based on data derived from self-reports which are highly susceptible to various biases [79]. Furthermore, some 2017–18 NHS questions focused on behaviour from the previous week (e.g. alcohol, physical activity), while others on a usual week (e.g. fruit and vegetables consumption, smoking).

Given the nature of the survey questions, caution must be exercised in using the risk factor measures presented herein. While the estimates provide insights into the spatial variation, due to the ecological fallacy [80] and the often varying lag time between exposure (to a risk factor) and a cancer diagnosis [3], the estimates here cannot be used to establish individual-level associations between risk factors and cancer incidence. Moreover, as these estimates are derived from cross-sectional data, they do not enable inference into lifetime risky behaviour or causal relationships with cancer.

The second limitation, relevant to any spatial analysis of lattice data, is the modifiable areal unit problem (MAUP) [81]. The MAUP refers to the sensitivity of estimates to the specified definition of a *small area* (e.g. choice of partitioning and resolution). While we have presented our estimates for SA2s, which offers wide applicability, we acknowledge that this particular partitioning of Australia represents just one of countless possible configurations, each yielding unique results. Thus, the conclusions drawn from our estimates are inherently entwined with the choice of partitioning and resolution of the small areas we employed [82].

Thirdly, the accuracy of our estimates are conditional on the 2017–18 NHS exclusions (very remote areas, discrete Aboriginal and Torres Strait Islander communities and non-private dwellings [57]). Without data for these sub-populations, there is currently no way to assess the impact of these exclusions on modelled estimates from this survey.

Next, while the SHAA provides estimates by sex [4], our study, constrained by the sparsity of the survey data at the SA2 level, did not allow for a similar disaggregation. Given the evidence that health behaviours can depend on sex, the non sex-specific estimates generated in this work may suffer from inadvertent smoothing toward the mean.

The final limitation is that the quality, in terms of both bias and variance, of small area estimates can always be improved by using larger surveys. Although we used the best survey data available, in the future, linkage of multiple surveys could provide much larger sample sizes across Australia, enabling the production of higher resolution estimates.

In terms of future research directions, one approach could involve developing distinct models for each of the eight risk factor measures. That is the linear predictor for each risk factor measure could have different sets of covariates, random effect structures or even include non-linear relationships via splines. Alternatively, future work could model the numerous risk factors jointly by leveraging univariate stage 1 models, followed by a multivariate spatial stage 2 model [83].

## Conclusions

Using a Bayesian two-stage small area estimation model we have, for the first time, generated and validated point estimates of the prevalence of eight cancer risk factors, and measures of their uncertainty, at the SA2 level across Australia. By aggregating the estimates, we have shown that they are very similar to those given by the SHAA [4], external surveys [84–89] and previous research on how area level socioeconomic status and remoteness relate to healthy behaviours [42]. The new estimates provide improved spatial resolution and reach and will enable more targeted cancer prevention strategies at the small area level. Furthermore, by including the results in the next release of the Australian Cancer Atlas [2], this work promises to provide a more comprehensive picture of cancer in Australia. Since the health factors used in this study are also common risk factors for other diseases, the prevalence estimates generated here may be useful in other disease modelling applications both in Australia and internationally.

### Supplementary Information

**Additional file 1.** Additional material containing further details of the data and model, and more plots, maps, and results.

## Data Availability

The 2017–18 National Health Survey microdata cannot be shared publicly due to the ABS privacy policy. An application can be made to the ABS directly to gain access to the data. The statistical analysis has been conducted in the secure ABS DataLab online computing environment. The modelled estimates are available on GitHub [72].

## Abbreviations

ABS: Australian Bureau of Statistics
ACA: Australian Cancer Atlas
ACT: Australian Capital Territory
AIHW: Australian Institute of Health and Welfare
ASGS: Australian Statistical Geography Standard
BMI: Body Mass Index
DOH: Department of Health
CCQ: Cancer Council Queensland
EP: Exceedance probability
HPDI: Highest posterior density interval
IQR: Interquartile range
IRSD: Index of Relative Socio-Economic Disadvantage
MCMC: Markov Chain monte carlo
MrP: Multilevel regression and poststratification
NHMRC: National Health and Medical Research Council
NHS: National Health Survey
NSW: New South Wales
NT: Northern Territory
PC: Principal component
PHN: Primary health network
QLD: Queensland
SA: South Australia
SA2: Statistical area level 2
SA3: Statistical area level 3
SA4: Statistical area level 4
SEIFA: Socio-Economic Indexes for Areas
SES: Socioeconomic status
SHAA: Social Health Atlases of Australia
TAS: Tasmania
TSLN: Two-stage logistic-normal
VIC: Victoria
WA: Western Australia
