# Supplementary material for: Mapping the prevalence of cancer risk factors at the small area level in Australia
Source: Int J Health Geogr. 2023 Dec 19;22:37. doi: 10.1186/s12942-023-00352-5 (PMC10729400; doi:10.1186/s12942-023-00352-5)
Supplement: Supplementary file 1 — Additional file 1. Additional material containing further details of the data and model, and more plots, maps, and results. [file 12942_2023_352_MOESM1_ESM.pdf]

Additional File for "Mapping the prevalence of cancer risk factors at the small area level in Australia"

James Hogg[1] **Jessica Cameron**[1,2] **Susanna Cramb**[1,3] **Peter Baade**[1,3] **Kerrie Mengersen**[1]

[1]Centre for Data Science, Queensland University of Technology
[2]Viertel Cancer Research Centre, Cancer Council Queensland

[3]Australian Centre for Health Services Innovation, School of Public Health and Social Work, Queensland University of Technology

Contents

A Other sources of Australian health data 3

A.1 State surveys 3

A.2 Other surveys 4

B Risk factor details 6

B.1 Smoking 6

B.2 Alcohol 6

B.3 Diet 6

B.4 Weight 6

B.5 Physical activity 7

C Model details 8

C.1 Survey weights 8

C.2 Principal components analysis 8

C.3 Stage 1: Individual level model 8

C.4 Stage 2: Area level model 9

C.4.1 Fixed effects 9

C.4.2 Fixed effects with varying coefficients 9

C.4.3 Estimates from the SHAA 10

C.4.4 Random effects 10

C.4.5 Stage 2 model 11

C.4.6 Generalized variance functions 11

C.5 Validation 11

C.5.1 Bayesian benchmarking . . . 11
C.5.2 Validation for very remote regions and the Northern Territory . . . 12
C.5.3 External validation . . . 12
C.5.4 Results . . . 12

D Additional data details **14**
D.1 Population Health Areas (PHA) . . . 14
D.2 Estimates from the SHAA . . . 14
D.3 Evidence classifications . . . 16

E Model building **17**
E.1 Stage 1 model . . . 17
E.2 Stage 2 model . . . 17

F Additional results **23**
F.1 Smoking . . . 23
F.2 Alcohol . . . 23
F.3 Diet . . . 23
F.4 Weight . . . 23
F.5 Physical activity . . . 23

G Additional plots **24**

H Additional maps **33**
H.1 Socioeconomic status . . . 33
H.2 Current smoking . . . 34
H.3 Risky alcohol consumption . . . 37
H.4 Inadequate diet . . . 40
H.5 Obese . . . 43
H.6 Overweight . . . 46
H.7 Risky waist circumference . . . 49
H.8 Inadequate activity (leisure) . . . 52
H.9 Inadequate activity (all) . . . 55

I Abbreviations **58**

A Other sources of Australian health data

By evaluating the extent to which our estimates at the SA2 level aligned with those from the SHAA and the broader socioeconomic status (SES), and remoteness trends observed in external surveys, we were able to validate the credibility and generalizability of our results. In this section, we provide details about these external surveys and their appropriateness with respect to the validation procedure carried out in this work. Note that our analyses did not involve any form of record linkage.

A.1 State surveys

Although the National Health Survey is the optimal survey for this study given its national coverage and breadth of health data collected, each state and territory of Australia also independently conduct surveys for health surveillance purposes.

New South Wales (NSW) NSW, the state with the largest 2016 adult population in Australia, conducts annual population health surveys by telephone. These surveys generally aim for a sample size of 13,000 people from the state [1]. The NSW Ministry of Health provides data on cancer risk factors stratified by year, remoteness, and SES status via their interactive dashboard, HealthStats NSW [2]. As shown in Fig. 1, trends in cancer risk factors from NSW contribute a large proportion of the data we used to externally validate our modelled estimates.

Victoria (VIC) VIC, the second largest state in terms of population, also conducts annual health surveys, which are administered using computer assisted telephone interviews [3]. In 2017, the survey collected data on 33,654 adults. Unlike other states, the Victorian Agency for Health Information provides cancer risk factor prevalence estimates for small areas called Local Government Areas (LGAs) [4], which are non-ASGS boundaries that continue to be used by the Australian government. Unfortunately data are not reported by remoteness or SES.

Queensland (QLD) QLD, the second largest state in terms of population, conducts annual health surveys, which are also administered by telephone. Around 12,500 adults participate each year [5]. Although not as flexible as the NSW data system, Queensland Health provide an online tool called the Queensland survey analytic system that provides prevalence estimates for cancer risk factor by LGAs, PHNs, remoteness and SES. Although LGA estimates are available, for the regions we would be interested in — far north Queensland for example — data are not available or not releasable.

Western Australia (WA) WA also conducts annual health surveys administered using computer assisted telephone interviews. In 2018, the sample included 5,750 adults aged 16 years and over and boasted an average participation rate of approximately 90% [6]. These data would have been important for validation as 5% of the states SA2s are very remote. Unfortunately there is no publicly available dashboard summarising the Western Australian survey results. In published reports, cancer risk factor data are not stratified by small area, remoteness or SES, which limits their usefulness for validation purposes.

South Australia (SA) SA collects health data monthly via telephone interviews. In one year, around 7,000 South Australians are interviewed for the survey [7]. Unfortunately there is no publicly available dashboard summarising the SA survey results. In published reports prevalence of cancer risk factors are sporadically stratified by remoteness, and SES.

Northern Territory (NT) The first state-wide health survey in the NT was due to occur in 2022 with the goal of sampling 2000 residents [8]. At the time of writing, the most relevant health data for the state is available in the NHS [9] (6.7% of participants were from the NT) or National Aboriginal and Torres Strait Islander Health Survey [10] (18% of participants were from the NT).

Tasmania (TAS) TAS has been collecting health data triennially since 2009 via computer assisted telephone interviews. The 2019 Tasmanian Population Health Survey sampled 6300 adults. The published report presented detailed prevalence estimates by remoteness, and SES [11].

Australian Capital Territory (ACT) The ACT has been conducting an annual General Health Survey since 2007 via computer assisted telephone interviewing [12]. In 2019, the survey collected a wide range of health data from 2002 adults. Given its population (only 1.7% of the 2016 adult population) and geographical size (less than 0.03% of Australia's landmass), data from the ACT were not informative for external validation in this research. The ACT is also relatively homogeneous in terms of remoteness and SES. Of its 114 SA2s, 110 are major cities and 102 have SES index deciles above 5 (denoting lower levels of disadvantage).

A.2 Other surveys

Other nationwide surveys have health data for either a subset of the population (like the National Aboriginal and Torres Strait Islander Health Survey (NATSIHS) [10]) or a subset of variables (such as the National Drug Strategy Household Survey (NDSHS) [13]).

The NATSIHS is a hexennial survey which collects social and health data from First Nations Australians with the goal of reporting statistics at the national and remoteness level [10]. The 2015 survey collected data on 11,178 First Nations Australians living in private dwellings across Australia. These surveys collect information on all the risk factors considered in this research.

The NDSHS is the leading survey of licit and illicit drug use in Australia. In 2019, the household-based survey collected data from 22,274 people aged 14 and over [13]. Relevant to this research, the NDSHS provides data on alcohol consumption and smoking.

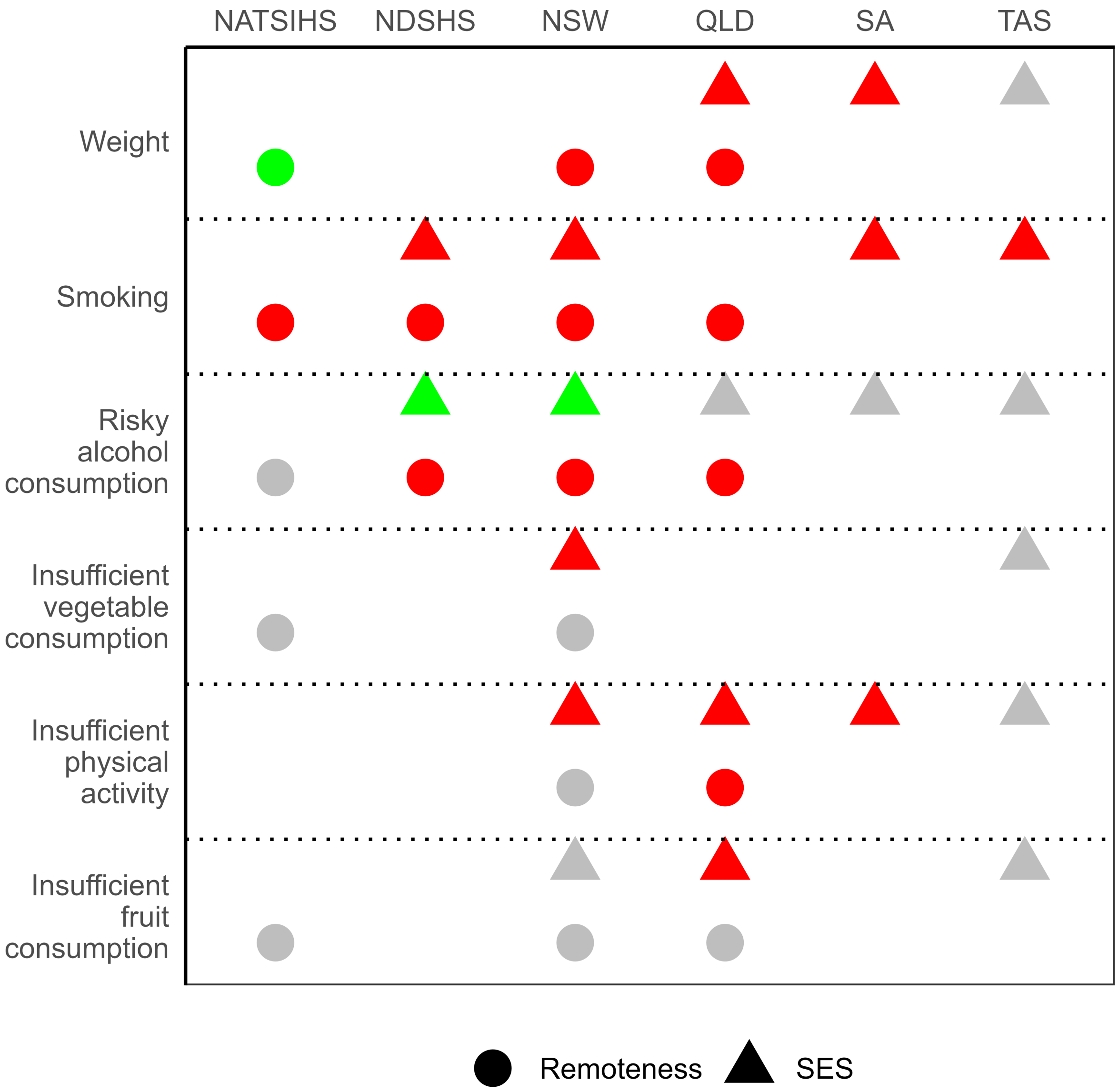

Figure 1: Schematic summarizing the key findings from external health surveys conducted by state governments (New South Wales (NSW) [2], Queensland (QLD) [5], South Australia (SA) [7] and Tasmania (TAS) [11]) and the Australian Bureau of Statistics (The National Aboriginal and Torres Strait Islander Health Survey (NATSIHS) [14] and National Drug Strategy Household Survey (NDSHS) [13]). More details can be found in Section A and D. Each column corresponds to a specific survey, while rows represent different cancer risk factors, each with varying definitions across the surveys. Within each risk factor, we provide a summary of trends based on remoteness and socioeconomic status (SES), represented by distinct shapes. The colors of the shapes indicate the direction of the trends: red indicates a higher prevalence for remote areas compared to urban areas, and for socioeconomic disadvantage compared to socioeconomic advantage. For instance, a red triangle signifies a higher prevalence of the risk factor in more remote areas, while gray indicates insignificant or trivial differences between remoteness categories. Conversely, green denotes a lower prevalence of the risk factor in more remote areas. The schematic only includes trends supported by available data from digital platforms or publicly available reports.

B Risk factor details

This section provides further details for the eight risk factor measure definitions used in this work. A summary is provided in Table 1 in the main paper.

B.1 Smoking

According to Whiteman *et al.* [15] and the 2015 Australian Burden of Disease study [16] smoking contributes the highest proportion of total cancer burden in Australia. Similar to the definition used in the SHAA [17], *current smoking* was defined as those who reported to be daily, weekly or less than weekly current smokers, and had smoked at least 100 cigarettes in their life. Even though ex-smokers experience greater risk than non-smokers for some cancers [18], including ex-smokers in the definition would create a measure of lifetime smoking which deviates from the cross-sectional nature of the other risk factor measures.

B.2 Alcohol

The revised 2020 guidelines by the NHMRC stipulate that persons should drink no more than 10 standard drinks a week and no more than 4 standard drinks on any one day [19]. The previous 2009 NHMRC guidelines stipulated that adults should drink no more than 2 standard drinks on any day [15]. Both versions of the guidelines emphasize that the recommendations do not represent a “safe” or “no risk” level of alcohol consumption [20].

Although Cancer Australia [21] and overseas governments, such as the US [22], recommends alcohol consumption in line with the 2009 NHMRC guidelines, Cancer Council Australia [23] recommends the more recent 2020 guidelines. In this work, we defined *risky alcohol consumption* as persons who did not meet the 2020 guidelines.

In the 2017-18 NHS, detailed alcohol consumption data were based on self-reports. Participants were asked about their alcohol consumption for the most recent three days (from the preceding 7 days) that they drank alcohol. Results cannot indicate lifetime alcohol behaviour.

B.3 Diet

Although a variety of diet-related factors, such as fruit, vegetables, meat, fibre and wholegrains, have been found to contribute to the risk of developing cancer [24, 16, 15], the 2017-18 NHS collected diet information for fruit and vegetables only [25]. Participants of the survey were asked to report the number of serves of fruit and serves of vegetables they usually ate each day.

Both Whiteman *et al.* [15], the SHAA [17] and Cancer Australia [21] use the 2013 NHMRC guidelines [26] for fruit and vegetables, which stipulates two serves (equivalent to 300g) of fruit and five serves (equivalent to 375g) of vegetables per day.

While we acknowledge the benefits in producing separate maps for fruit and vegetable consumption, we found that only 7.5% of Australians met the guidelines for vegetable intake [27]. Modelling these sparse data could give very unstable prevalence estimates [28], a possible reason why the SHAA does not provide estimates for vegetable consumption alone. To address this issue, we jointly modelled whether participants met the guidelines for fruit or vegetables. Specifically, we assigned a value of one, indicating *inadequate diet*, to persons who did not meet either guideline.

B.4 Weight

According to Whiteman *et al.* [15], 3.4% of cancer diagnoses in Australia are attributable to being overweight or obese. Following the SHAA, we used both weight-based risk factor measures according to the conventional Body Mass Index (BMI) [29, 30], and a measure related to waist circumference [31].

BMI is calculated using a participant’s height and weight. While self-reported height and weight are commonplace [32, 33] and useful to approximate BMI [34, 35], the reported values can be subject to bias [36]. Measurement of weight, height and waist circumference was a voluntary section of the NHS interview. To increase the applicability (reduce the number of missing values) of these weight-based measurements, any participant who refused to be measured had their weight, height and waist circumference measurements imputed using a hot-decking method where they received weight, height and waist circumference measurements from another very similar participant based on demographic characteristics, such as age, sex, state, self-perceived body mass, exercise, cholesterol and self-reported BMI [25]. Although approximately 40% of the measured data were imputed, measured values can have significantly less bias than self-reported measures [32]. In this work, we used the measured data.

Overweight/obese and *obese* was defined as persons with a BMI greater than or equal to 25 and 30, respectively.

Following Cancer Australia [21] and the SHAA [17], we defined *risky waist circumference* as measurements of 94cm and 80cm or more for men and women, respectively [20]. Note that these waist circumference cutoffs are only appropriate for adults, so for this risk factor we limit the dataset to all persons 18 years and older. Assuming that the single-year age distribution in any of the age group was uniform, we estimated that the population of 18-19-year olds was 40% of the 15-19-year old population.

B.5 Physical activity

The national Department of Health (DOH) guidelines for physical activity, published in 2014 [37], closely mirror those given by the World Health Organization [38]. The DOH guidelines stipulate that each week adults (those between the ages of 18 and 64) should either do 2 $1/2$ to 5 hours of moderate-intensity physical activity or 1 $1/4$ to 2 $1/2$ hours of vigorous-intensity physical activity or an equivalent combination of both. In addition, the guidelines recommend muscle-strengthening activities at least 2 days each week. The DOH guidelines also provide specific recommendations for children (5 to 17 years), older persons (65 years and older) and pregnant women.

The benefits of physical activity on overall health, including cancer, are best described by considering the total volume of activity, which is calculated by considering the frequency, duration and intensity of exercise [39]. In a systematic review of physical activity and cancer outcomes, McTiernan *et al.* [40] found that most studies related leisure physical activity to cancer, whereas non-leisure (or work-related) physical activity was not classed as physical activity. Elsewhere [41], physical activity has been defined as any movement that results in energy expenditure, which includes deliberate exercise or sport, incidental movement or work-related activity.

To capture both leisure only and all activity, we opt to allow workplace physical activity (if of sufficient intensity) to count toward meeting the 2014 DOH guidelines. *Inadequate activity (leisure)* was defined as those who did not meet the guidelines based on their reported leisure physical activity alone in the week before the survey interview. *inadequate activity (all)* was defined as those who did not meet the guidelines based on their reported leisure and workplace physical activity in the week before the survey interview. In this work, the physical activity measures were derived from the ABS created variables that accommodated the guidelines across age groups.

C Model details

This section provides details of the two-stage model described in Section 3 of the main paper. Figures 2 and 3 (in the main paper) provide graphically summaries of the components of the two-stage model. Initially we describe the principal components analysis that was carried out before modelling.

C.1 Survey weights

To help correct for sampling bias and promote design-consistency, survey practitioners provide survey weights. In this analysis, we used two versions of the survey weights, w_{ij}^{raw}, provided by the ABS [25, 42].

$$w_{ij} = n_i w_{ij}^{\text{raw}} \left(\sum_{j=1}^{n_i} w_{ij}^{\text{raw}} \right)^{-1}$$

was used for direct estimation, and

$$\tilde{w}_{ij} = n w_{ij}^{\text{raw}} \left(\sum_{i=1}^{m} \sum_{j=1}^{n_i} w_{ij}^{\text{raw}} \right)^{-1}$$

was used in the stage 1 model.

C.2 Principal components analysis

As mentioned in Section 2.2.2 in the main paper, principal components analysis was conducted on 84 continuous census covariates represented as proportions or averages. The input data were scaled and centered prior to the analysis. Principal component 1 (PC1) to PC6 were retained as they accounted for approximately 62% of the variation. PC1 to PC6 captured approximately 24%, 11%, 11%, 7%, 5% and 4% of the variation, respectively. All remaining PCs contributed less than 3% of the total variation each with 83% of these contributing less than 1%.

PC1 was very strongly correlated (Pearson correlation of 0.86) with the SES index deciles (see Fig. 4), obtained from the ABS Socio-Economic Indexes for Areas product. Despite the correlation, model performance (assessed using metrics described in Section E) was improved when both were included. Including both was pragmatic as the SES index is constructed from 16 purposely-selected census measures of relative disadvantage, while PC1 to PC6 captured a wider range of census data. Moreover, the SES index is included as a categorical variable with ten groups, whilst the PC1 to PC6 variables are included as continuous quantities. A further benefit of including the SES index (which captured 43% of the variation) is that it accommodates some information not captured by the principal components analysis including characteristics of dwellings (internet connection, number of cars, etc), disability and equivalised income [43].

C.3 Stage 1: Individual level model

In this section we describe the components of the stage 1 linear predictor,

$$\text{logit}\left(\pi_{ij}\right) = \mathbf{X}_{ij}\boldsymbol{\beta} + e_i + \delta_{r[ij]} + \xi_{d[ij]} + \epsilon_{ij},$$

for the individual level probability of $y_{ij} = 1$ for sampled individual $j = 1, \ldots, n_i$ in SA2 $i = 1, \ldots, m$. Details on how the model was selected are given in Section E.1.

Fixed effects The fixed effects were included via the individual level design matrix, $\mathbf{X}_{ij}$, and corresponding coefficients, $\boldsymbol{\beta}$. We used the following individual level categorical covariates in the stage 1 models: age, sex and their interaction; registered marital status; hypertension status; Kessler psychological distress score; occupation; language spoken at home; and depression status. See Table 2 for the categories of the individual level covariates and the reference groups used. Along with the individual covariates, we also included seven SA2 level fixed effects; SES index deciles (IRSD) as a categorical covariate with 10 groups and the first six principal components (PC) (derived from the 2016 census data, see Section C.2).

Random effects Unstructured individual level random effects (ϵ_{ij}) and area (SA2) level random effects (e_i) were applied. In addition to these, by borrowing ideas from MrP [44], we included two hierarchical random effects based on categorical covariates that were themselves derived from the interaction of numerous individual level demographic and health covariates. We derived a demographic-health (DH) categorical covariate from the interaction of sex, age, self-assessed health, qualification, and high school completion status. See Table 2 for the categories of these covariates. The median (IQR) samples size in each DH group was 7 (3, 20). The random effect for DH group $d = 1, \ldots, 1050$ is denoted by $\xi_{d[ij]}$, where $d[ij] \in \{1, \ldots, 1050\}$ indexes the DH group for sampled individual j in SA2 i.

We also derived a categorical covariate ($\delta_{r[ij]}$ with $r = 1, \ldots, 16$ levels) from the interaction of the binary risk factor outcomes not directly associated with the risk factor being modelled. See Table 3 for more details on this covariate, termed the *non-outcome risk factor (NORF)* covariate. For example, when modelling waist circumference, $\boldsymbol{\delta} = (\delta_1, \ldots, \delta_{16})$ was constructed using risky alcohol consumption, inadequate activity (all), inadequate diet and current smoking. This variable was identical for risky waist circumference, obese and overweight/obese, while for other outcomes, overweight was one of the binary risk factors included.

The probability that $y_{ij} = 1$ for sampled individual j in SA2 i is denoted by π_{ij}. Pseudo-likelihood was used to ensure the predictions from the logistic model were approximately unbiased under the sample design [45, 46]. The remaining notation is described in Section 3.1 of the main paper.

Stage 1 model The stage 1 model used for all risk factor measures is,

$$\begin{aligned} y_{ij} &\sim \text{Bernoulli}\left(\pi_{ij}\right)^{\tilde{w}_{ij}} \\ \text{logit}\left(\pi_{ij}\right) &= \mathbf{X}_{ij}\boldsymbol{\beta} + e_i + \delta_{r[ij]} + \xi_{d[ij]} + \epsilon_{ij} \\ e_i &\sim \text{N}\left(0, \sigma_e^2\right) \\ \delta_{r[ij]} &\sim \text{N}\left(0, \sigma_\delta^2\right) \\ \xi_{d[ij]} &\sim \text{N}\left(0, \sigma_\xi^2\right) \\ \epsilon_{ij} &\sim \text{N}\left(0, 2^2\right), \end{aligned} \qquad \text{Eq. C.3.1}$$

where we represent the pseudo-likelihood for a probability density, $p(.)$, as $p\left(y_{ij}\right)^{\tilde{w}_{ij}}$ [42]. Note the fixed variance for ϵ_{ij}; an explanation is given in Section E. Details on the priors used are given in Section 3.2 in the main paper.

C.4 Stage 2: Area level model

In this section we describe the components of the stage 2 linear predictor,

$$\theta_i = \mathbf{Z}_i\boldsymbol{\Lambda} + \alpha\gamma_i + \mathbf{G}_i\boldsymbol{\Gamma}_{r[i]} + \zeta_i + \eta_{h[i]},$$

for SA2 $i = \ldots, M$, where $M = 2221$ is the total number of areas. Details on how the model was selected are given in Section E.2.

C.4.1 Fixed effects

The design matrix of fixed effects ($\mathbf{Z}_i$) for the second stage model with corresponding coefficients ($\boldsymbol{\Lambda}$) included two categorical covariates: the SES index deciles (IRSD) and remoteness. Apart from the six principal components (introduced below), no other specific census variables were found to improve model fit.

C.4.2 Fixed effects with varying coefficients

The second component of the linear predictor for θ_i was the six continuous covariates, PC1 to PC6. Australia is highly decentralised, meaning that area level statistical relationships may be very different in major cities as opposed to very remote areas. To incorporate this, we allowed the fixed effect regression coefficients for PC1 to PC6 to vary according to remoteness (major cities, inner regional and outer regional to very remote).

The principal components values for SA2 i are denoted by $\mathbf{G}_i$, a row-vector of length six. The regression coefficients specific to the rth remoteness category for the ith SA2 are denoted by $\boldsymbol{\Gamma}_{r[i]}$, a column-vector of length six. Although we explored partial pooling for the regression coefficients, because we only used three remoteness groups we found better performance and convergence when using independent priors; treating them as fixed effects. As described in Section 3.2 in the main paper, fixed effects were given generic weakly informative priors. Thus, $\boldsymbol{\Gamma}_{r[i]} \sim \text{N}\left(0, 2^2\right)$.

C.4.3 Estimates from the SHAA

The third component of the stage 2 linear predictor was modelled prevalence estimates from the SHAA at the PHN level (see Section D). The data from the SHAA is provided as age-standardised rates per 100 people with 95% confidence intervals. We used these data to derive proportion estimates and their associated standard errors, before transforming the estimates to the unconstrained scale.

The logistic transformed SHAA estimate and variance for SA2 i are denoted by $\hat{\gamma}_i$ and $\widehat{\mathrm{v}}\left(\hat{\gamma}_i\right)$, respectively. Note that $\hat{\gamma}_i$ and $\hat{\gamma}_k$ (and corresponding variance) were identical if SA2 i and k were within the same PHN.

To accommodate the variance of the estimates from the SHAA, we assumed classical measurement error [47], whereby the model variance (or error) is independent of the true value. Thus,

$$\begin{aligned}\hat{\gamma}_i &\sim \mathrm{N}\left(\gamma_i, \widehat{\mathrm{v}}\left(\hat{\gamma}_i\right)\right)\\ \gamma_i &\sim \mathrm{N}\left(0, 2^2\right)\end{aligned}$$

where γ_i is assumed to be the true logistic transformed SHAA estimate. Note that other work models the true values using spatial priors [48, 49, 50]. However, because there are few PHNs compared to SA2s, we used an independent weakly informative prior, $\mathrm{N}\left(0, 2^2\right)$.

The true SHAA estimate, γ_i, was included as an external latent field in the linear predictor for θ_i, with α (the coefficient for γ_i) controlling the influence of the external latent field on the modelled estimates. Table 5 shows which of the variables from the SHAA was used as the external latent field for each of our risk factor measures.

C.4.4 Random effects

The final components of the stage 2 model were random effects. Many studies have illustrated the benefits of accommodating the spatial structure of the small areas in SAE models [51, 52, 48, 53, 54]. Although others have used conditional autoregressive (CAR) [55] or simultaneous autoregressive (SAR) priors only, Gomez-Rubio *et al.* [56] argues that including a structured and unstructured random effect provides a useful compromise between accurate small area estimates and their variances.

We used the BYM2 spatial prior [57] (ζ_i) with mixing parameter, $\rho \in [0, 1]$, scaling factor, κ, variance parameter, σ^2_ζ, and $M \times M$ adjacency matrix, $\mathbf{W}$. The BYM2 prior is a linear combination of a unit-scale intrinsic conditional autoregressive (ICAR) prior [55] and a standard normal. The BYM2 prior, denoted as $\mathrm{BYM2}\left(\mathbf{W}, \kappa, \rho, \sigma^2_\zeta\right)$, is

$$\begin{aligned}\zeta_i &= \sigma_\zeta\left(s_i\sqrt{\rho/\kappa} + v_i\sqrt{1-\rho}\right) \qquad \text{Eq. C.4.1}\\ s_i &\sim N\left(\frac{\sum_{k=1}^M W_{ik}s_k}{\sum_{k=1}^M W_{ik}}, \frac{1}{\sum_{k=1}^M W_{ik}}\right)\\ v_i &\sim N(0,1).\end{aligned}$$

As is common in disease mapping [58], we used the binary contiguous specification for $\mathbf{W}$ where $W_{ik} = 1$ if SA2 i and SA2 k are neighbors and zero otherwise. To reduce the complexity of our model we made several manual changes to the weight matrix.

- We ensured the neighborhood structure was fully connected [57] by treating the eastern and western SA2s at the top of Tasmania as neighbors of the furthest south SA2s in Victoria.
- Donut SA2s, or areas with only one neighbor (n = 87), were also assigned the neighbors of their neighbor. The final weight matrix assigned 93% of SA2s more than 2 neighbors.
- Although Jervis Bay is classified as an "Other Territory" by the ABS, we altered its SA2 code to include it as part of NSW.

Following the recommendations by Gomez-Rubio *et al.* [56], Mohadjer *et al.* [59] and Banerjee *et al.* [60], the ICAR prior for $\mathrm{s} = (s_1, \ldots, s_M)$ was declared for all areas and thus the s_i's for non-sampled areas are implicitly imputed during MCMC. We used the Stan implementation of the ICAR prior given by Morris *et al.* [61].

Given that we did not export, explore or include SA3 level census covariates, we employed a random effect at the SA3 level as well [56]. Let $\eta_{h[i]}$ be the random effect for SA3 h. We found no discernible improvement in model fit when using spatial priors at the SA3 level, so we reverted to a standard normal prior $\text{N}\left(0, \sigma^2_\eta\right)$ instead.

C.4.5 Stage 2 model

The stage 2 model used for all risk factor measures was,

$$\begin{aligned}
\hat{\boldsymbol{\theta}}_i^{\text{S1}} &\sim \text{N}\left(\theta_i, \bar{\tau}_i^{\text{S1}} + \widehat{\text{v}}\left(\hat{\theta}_i^{\text{S1}}\right)\right)^{1/\tilde{T}} \\
\theta_i &= \mathbf{Z}_i\boldsymbol{\Lambda} + \alpha\gamma_i + \mathbf{G}_i\boldsymbol{\Gamma}_{r[i]} + \zeta_i + \eta_{h[i]} \\
\hat{\gamma}_i &\sim \text{N}\left(\gamma_i, \widehat{\text{v}}\left(\hat{\gamma}_i\right)\right) \\
\zeta_i &\sim \text{BYM2}\left(\mathbf{W}, \kappa, \rho, \sigma^2_\zeta\right) \\
\eta_{h[i]} &\sim \text{N}\left(0, \sigma^2_\eta\right),
\end{aligned} \qquad \text{Eq. C.4.2}$$

where the prevalence estimate for the ith SA2 was given by the posterior distribution of $\mu_i = \text{logit}^{-1}\left(\theta_i\right)$. The remaining notation and details on the priors used are given in Section 3.2 in the main paper.

C.4.6 Generalized variance functions

As discussed in our previous work [28], the stage 1 sampling variances, ψ_i^{S1}, can be unrealistically low for unstable areas. To correct for this we used generalized variance functions (GVF), which are commonly used in area level SAE modelling to smooth or impute unstable sampling variances [62, 63, 64]. The GVF used in this work is a Bayesian linear model, fitted to the stable areas, and is a generalisation of the GVF used by Das *et al.* [65] to a fully Bayesian framework [66].

Let $\mathbf{L}$ be the design matrix and $\boldsymbol{\omega}$ the corresponding regression coefficients for the linear model. We used the log of the SA2 sample size, the log of the SA2 population, PC1 and the posterior median of $\hat{\theta}_i^{\text{S1}}$ as covariates. The GVF is,

$$\log\left(\sqrt{\psi_i^{\text{S1}}}\right) \sim \text{N}\left(\mathbf{L}_i\boldsymbol{\omega}, \sigma^2_{\text{gvf}}\right). \qquad \text{Eq. C.4.3}$$

During MCMC we impute values for the unstable S1 sampling variances via $\left(\exp\left(\mathbf{L}_i\boldsymbol{\omega} + 0.5\sigma^2_{\text{gvf}}\right)\right)^2$ [65].

C.5 Validation

C.5.1 Bayesian benchmarking

Let $\widehat{C}_k^D$ and $\widehat{\text{v}}\left(\widehat{C}_k^D\right)$ be the direct Hajek [67] estimate and sampling variance for benchmark $k = 1, \ldots, K$. The goal of internal benchmarking in this work was to ensure that the population-weighted modelled estimate,

$$\widetilde{C}_k = \frac{\sum_{i \in S_k} \mu_i N_i}{\sum_{i \in S_k} N_i} \qquad \text{Eq. C.5.1}$$

was in approximate agreement with $\widehat{C}_k^D$. Note that S_k denotes the set of SA2s in benchmark group k. Fully Bayesian benchmarking takes the form,

$$\widetilde{C}_k \sim N\left(\widehat{C}_k^D, \left(p \times \sqrt{\widehat{\text{v}}\left(\widehat{C}_k^D\right)}\right)^2\right) \qquad \text{Eq. C.5.2}$$

where $p > 0$ was a discrepancy measure used to assert the desired level of concordance. We set $p = 0.5$ for both the state and major-by-state benchmarks. Note that benchmarking was not used during model selection and was only applied once a final model had been selected.

C.5.2 Validation for very remote regions and the Northern Territory

The ABS warns that direct estimates for the Northern Territory (NT) could be inaccurate. This is because 20% of the population of the NT live in very remote or discrete First Nations Australian communities which were purposely excluded from the sampling frame [25]. According to data from 2006, the NT had the highest proportion of First Nations Australian people residing in discrete communities, 41,681 (45%) [68]. Of the 40 SA2s across Australia with populations that were composed of over 25% First Nations Australian people, 30 (75%) were very remote and 18 (45%) were in the NT, respectively.

Furthermore, given the following warning from the ABS [25],

> *. . . the estimates from the survey, do not (and are not intended to) match estimates of the total Australian estimated resident population (which include persons living in Very Remote areas of Australia and persons in non-private dwellings, such as hotels) obtained from other sources*

we could not use internal validation (benchmarking) for very remote SA2s.

C.5.3 External validation

External validation was performed by comparing the estimates to those from the SHAA at the PHA level and the overall trends observed in the modelled results with the general findings from other Australian health survey. It was not possible to match our definitions and age subset to those from the external data. Thus, comparisons were general in nature, assuming that general trends in risk factor prevalence would be evident.

The graphical summary presented in Section A of the Additional File provides an overview of the broader associations between the prevalence of cancer risk factors, the SES index, and remoteness. The figure summarises the trends available from published reports and/or digital platforms ranging across six selected Australian health surveys.

Due to the scope of the 2017-18 National Health Survey (NHS) [25], internal validation would not have been valid for very remote SA2s and those in the Northern Territory (n = 103) (see Section C.5.2 above). Although these SA2s only accounted for approximately 1.5% of the 2017-2018 adult population, they were of particular interest in this work since no estimates currently exist for many of these very remote areas.

C.5.4 Results

Our SA2 level modelled estimates corroborated well with the trends reported from the external surveys and SHAA estimates (Section A). The strongest evidence of agreement was for current smoking and obese as the risk factor measure definitions from this work and those from the SHAA were similar (see Fig. 2). Moreover, the trends by socioeconomic status and remoteness from the external surveys agreed strongly with the modelled estimates.

The level of agreement with external data was generally similar for benchmarked and non-benchmarked SA2s, with non-benchmarked SA2s having considerably higher uncertainty on average as expected (see Section G). This was particularly true when deriving a single estimate for the non-benchmarked SA2s (see Fig. 3). Our model-based estimate of this quantity was generally very different (often higher) and had far greater uncertainty than the direct estimate which, due to the scope of the survey, is advertised as non-robust [25]. This disparity was expected as the direct estimate did not capture any very remote areas, with most data coming from the capital of the Northern Territory, Darwin. In contrast, our model-based estimate captured SA2 estimates from Darwin *and* very remote Australia where population health is generally poorer [69].

The fully Bayesian benchmarking approach performed well, with minimal changes in point estimates. We also observed a relatively even spread of increased and decreased posterior uncertainty, ranging from about a 13% increase to a 6% decrease in the width of HPDIs when the benchmarks were accommodated (see Table 1).

Table 1: Descriptive statistics comparing the SA2 level prevalence estimates from the benchmarked and non-benchmarked models. The first column summarises the mean absolute relative difference (MARD) between the posterior median proportions from the benchmarked and non-benchmarked models. The second column of the table compares the width of the 95% highest posterior density intervals (HPDI) by dividing the non-benchmarked intervals sizes by the benchmarked interval sizes. Thus, a value greater than 1 indicates that the benchmarked model provides narrower HPDIs. We derived the ratio for all SA2 areas, but only display the median and interquartile range in the table.

	MARD $\times 10$	Median (IQR) of relative width of HPDIs
Inadequate activity (leisure)	0.13	1.01 (0.95, 1.07)
Inadequate activity (all)	0.15	1.06 (1.01, 1.12)
Risky alcohol consumption	0.60	1.00 (0.96, 1.05)
Inadequate diet	0.38	1.05 (1.02, 1.09)
Obese	0.51	0.95 (0.90, 1.01)
Overweight/ obese	0.23	0.96 (0.92, 1.02)
Current smoking	0.70	0.95 (0.90, 1.01)
Risky waist circumference	0.38	0.87 (0.81, 0.94)

D Additional data details

D.1 Population Health Areas (PHA)

There is substantial heterogeneity of the component SA2s within each PHA in terms of population size, socioeconomic status and remoteness. For example, 71 (6%) and 491 (42%) PHAs are composed of SA2s with different remoteness categories and different SES index deciles, respectively. The average standard deviation of SA2 population sizes within each PHA is 3254. The affect of aggregating these heterogeneous SA2s into PHAs is a loss of information, where the extent to the loss is somewhat dependent on the number of SA2s within each PHA. In 2016, there were 451 (39%) and 242 (21%) PHAs with 2 and more than 2 component SA2s, respectively.

D.2 Estimates from the SHAA

As described in Section 2.2.3 of the main paper, we also obtained prevalence estimates and measures of uncertainty for six risk factors from the SHAA [17] at the Primary Health Network (PHN) and PHA level for adults. There are 31 PHNs across Australia, with each comprising multiple SA2s (median of 72 and IQR of 53 to 96) and varying population sizes (median 505000 and IQR of 401000 to 808000, averaged over the 2017-2018 adult population). The SHAA's estimates and their respective uncertainties were included in the models and thus each SA2 was assigned to a PHN using ABS concordance files. To obtain a single value for any SA2 that overlapped several PHNs, we took the weighted mean of the PHN estimates using the given concordance ratios. Note that the SHAA does not provide estimates for the Western Queensland PHN. Based on its similar remoteness characteristics, estimates for this PHN were copied from those from the Northern Territory PHN.

Table 2: Categories for the individual level covariates used in this research (see Section C.3). Bolded categories denote the reference group. Most of the categories for these covariates were derived by the Australian Bureau of Statistics [9]. For details of the definitions, we refer the reader to publicly available data dictionaries [70].

	#	Categories
Sex	2	**Male**, Female
Age	9	**15-19**, 20-24, 25-34, 35-44, 45-54, 55-64, 65-74, 75-84, 85+
Registered marital status	5	**Married**, Never married, Widowed, Divorced, Separated
Hypertension	3	Has hypertension, **Does not have hypertension**<br>Not applicable
Language spoken at home	2	**English**, Other
Depression status	4	All or most of the time, Some or a little of the time<br>**None of the time**, Not available
Kessler score	3	High/very high, **Low/moderate**, Not available
Occupation	10	Community and personal service workers<br>**Unemployed or not in labourforce**<br>Clerical and administrative workers<br>Professsionals, Technicians and trades workers<br>Labourers, Sales workers<br>Machinery operators and drivers<br>Managers, Other
Self-assessed health	5	Poor, Fair, Good, Very good, **Excellent**
Qualification	4	Certificate, **No non-school qualification or not determined**<br>Bachelor/Diploma, Postgraduate
High school completion status	4	**Year 12 or equivalent**, Year 11 or equivalent<br>Year 10 or equivalent, At most year 9 or equivalent

Table 3: Description of the cancer risk factor measures used to construct the non-outcome risk factor (NORF) categorical variable, which was uniquely defined for each cancer risk factor measure. The NORF variable was used in the stage 1 model described in Section C.3.

		Measures included in NORF categorical covariate				
		Current smoking	Risky alcohol consumption	Inadequate diet	Overweight	Inadequate activity (all)
Risk factor measures	Current smoking		✓	✓	✓	✓
	Risky alcohol consumption	✓		✓	✓	✓
	Inadequate diet	✓	✓		✓	✓
	Obese	✓	✓	✓		✓
	Overweight/ obese	✓	✓	✓		✓
	Risky waist circumference	✓	✓	✓		✓
	Inadequate activity (leisure)	✓	✓	✓	✓	
	Inadequate activity (all)	✓	✓	✓	✓	

Table 4: Definitions for the six cancer risk factor measures used from the SHAA [17].

SHAA variable	Definition
Current smokers	Those who were classed as current smokers.
Risky alcohol consumption	Those who consumed more than two standard alcoholic drinks per day on average.
Adequate fruit intake	Those who consumed at least 2 serves of fruit per day on average.
Obese	Those with a BMI greater or equal to 30.
Overweight	Those with a BMI that was between 25 and less than 30.
Inadequate physical activity	Those who undertook low, very low or no exercise in the week prior to the survey.

Table 5: Table summarising which of the variables from the SHAA was used as fixed effects in the stage 2 model (Section C.3) for each of the cancer risk factor measures.

		SHAA variables					
		Smoking	Alcohol	Fruit	Obese	Overweight	Exercise
Risk factor measures	Current smoking	✓					
	Risky alcohol consumption		✓				
	Inadequate diet			✓			
	Obese				✓		
	Overweight/ obese					✓	
	Risky waist circumference					✓	
	Inadequate activity (leisure)						✓
	Inadequate activity (all)						✓

D.3 Evidence classifications

We followed the work by Gramatica *et al.* [71] and Congdon *et al.* [72], who used an adaption of the LISA (Local Indicator of Spatial Association) clustering approach [73] to identify clusters of areas with significantly high (or low) prevalence. The LISA clusters were computed from the posterior draws by first deriving the deviation of the prevalence from the national average, $z_i^{(t)} = \mu_i^{(t)} - \hat{\mu}^D$, and then calculating the spatial lag of the deviation, $\mathbf{L}^{(t)} = \mathbf{z}^{(t)} \left(\mathbf{W}^*\right)^T$, where $\mathbf{z}^{(t)} = \left(z_1^{(t)}, \ldots, z_M^{(t)}\right)$ was a M-dimensional row vector and $\mathbf{W}^*$ the row-standardized version of the adjacency neighborhood matrix described in Section C.4.4.

To derive the evidence classification measure, SA2s were classified into one of four exclusive groups [72]:

- High-cluster ("HC") if both $\frac{1}{T}\sum_t \mathbb{I}\left(z_i^{(t)} > 0\right) > 0.8$ and $\frac{1}{T}\sum_t \mathbb{I}\left(L_i^{(t)} > 0\right) > 0.8$,
- High ("H") if $\frac{1}{T}\sum_t \mathbb{I}\left(z_i^{(t)} > 0\right) > 0.8$ and $\frac{1}{T}\sum_t \mathbb{I}\left(L_i^{(t)} > 0\right) \leq 0.8$
- Low ("L") if both $\frac{1}{T}\sum_t \mathbb{I}\left(z_i^{(t)} > 0\right) < 0.2$ and $\frac{1}{T}\sum_t \mathbb{I}\left(L_i^{(t)} > 0\right) \geq 0.2$,
- Low-cluster ("LC") if both $\frac{1}{T}\sum_t \mathbb{I}\left(z_i^{(t)} > 0\right) < 0.2$ and $\frac{1}{T}\sum_t \mathbb{I}\left(L_i^{(t)} > 0\right) < 0.2$.

E Model building

As described in Section 3.1 in the main paper, a single model specification was chosen to be applied to all eight risk factor measures.

E.1 Stage 1 model

To guide model selection for the stage 1 model, we used the smoothing ratio (SR) and area linear comparison (ALC) metrics, both of which indicate the level of concordance between the observed and smoothed data from the stage 1 model [28]. The SR and the ALC smoothing metrics achieve this by comparing the observed individual level data to the predicted probabilities and the area level direct estimates to the stage 1 estimates, respectively. Higher values of both are preferred. We used the posterior median of the SR, which for posterior draw t is given by,

$$SR^{(t)} = 1 - \frac{\sum_{i=1}^{m} \left| \frac{\sum_{j=1}^{n_i} w_{ij} \left(y_{ij} - \pi_{ij}^{(t)} \right)}{n_i} \right|}{\sum_{i=1}^{m} \left| \frac{\sum_{j=1}^{n_i} w_{ij} (y_{ij} - \hat{\mu}^D)}{n_i} \right|}, \quad \text{Eq. E.1.1}$$

where $\hat{\mu}^D$ is the overall prevalence. The ALC is equal to the regression coefficient when we regress the posterior median of $\hat{\mu}_i^{S1}$ on $\hat{\mu}_i^D$ with weights $1/\psi_i^D$.

For survey-only and census fixed effects, a frequentist weighted logistic model with no random effects was used for variable selection. The primary focus in selecting these was to maximize both the ALC and the SR, with AIC and BIC considered as secondary criteria. When validating frequentist decisions with Bayesian inference we used leave-one-out cross-validation (LOOCV) via Pareto-importance sampling [74]. In general the selected variables were consistently good predictors across all the risk factor measures. See Table 6 and 7 for model metrics for the stage 1 model for all risk factor measures.

To select the variables to include in the demographic-health categorical covariate, we explored a large range of possible interactions by fitting the resulting categorical variable using frequentist weighted logistic mixed models. The selected set of covariates showed persistent benefits, in terms of the ALC and the SR, across all risk factor measures. Similar to our previous work [28], we found that fitting the NORF categorical variable as a random effect improved model fit across the board (see Section D).

The fixed standard deviation of the residual error (σ_e) was utilized to address stage 1 models in cases where the SR or the ALC was too low. In previous work we found that optimal performance of the area level MRRMSE and MARB generally occurred when the $0.55 < ALC < 0.75$ and the $0.4 < SR < 0.7$, with performance atrophy outside these bounds. We found that values in the lower end of these bounds generally provided narrower and more reliable credible intervals.

As discussed in our previous work [28], although the stage 1 model is designed to smooth the observed data, over-smoothing (e.g. when the SR or the ALC are close to zero) can significantly affect the model performance. Thus, the residual error scale was used to purposely overfit the first-stage model in order to improve the predictions from the second-stage model. In this work, we set $\sigma_e = 2$, which provided a range of the SR from 0.37 to 0.45 and the ALC from 0.55 to 0.70 across the risk factor measures.

E.2 Stage 2 model

Unlike other SAE applications where predictive accuracy can be reasonably assessed via scoring rules [54] or leave-one-out cross validation [74], given the unstable nature of the direct SA2 level estimates, we assessed performance of our stage 2 model by comparing direct and modelled estimates at two aggregated levels; the SA4 and major-by-state benchmark level. At these levels of aggregation, the direct estimates were plausibly treated as ground truth. For example, approximately 73% and 100% of the direct smoking estimates at the SA4 and major-by-state benchmark level had coefficients of variation below 25%. Direct estimates for other risk factors exhibited similar or superior certainty.

Under the assumption that the SA4 and major-by-state benchmark level direct estimates were the truth, we used the Bayesian analogue of mean absolute relative bias (MARB) and mean relative root mean square error (MRRMSE) to assess model performance. Below we give details at the major-by-state benchmark level using the notation given in Section 3.3.1 of the main paper.

$$\text{MARB} = \frac{1}{K}\sum_{k=1}^{K}\left|\frac{\frac{1}{T}\sum_{t=1}^{T}\left(\widehat{C}_k^D - \widetilde{C}_k^{(t)}\right)}{\widehat{C}_k^D}\right| \quad \text{Eq. E.2.1}$$

$$\text{MRRMSE} = \frac{1}{K}\sum_{k=1}^{K}\frac{\sqrt{\frac{1}{T}\sum_{t=1}^{T}\left(\widehat{C}_k^D - \widetilde{C}_k^{(t)}\right)^2}}{\widehat{C}_k^D} \quad \text{Eq. E.2.2}$$

In addition to MARB and MRRMSE, we also derived the interval overlap probability (IOP) for each SA4 and major-by-state benchmark estimate. An IOP of 1 indicated that the modelled 95% highest posterior density interval (HPDI) was entirely contained within the direct estimate 95% confidence interval; the optimal situation. To summarizethe IOPs, we used the mean IOP (MIOP). Given that SA4 level direct estimates were still relatively unstable, we placed greater priority on the MIOP than the MARB and MRRMSE during model selection. See Table 8 and 9 for model metrics for the stage 2 model for all risk factor measures.

Table 6: The progression of the performance metrics (smoothing ratio (SR), area linear comparison (ALC) and leave-one-out cross validation (LOOCV)) as components of the linear predictor for the stage 1 model are included. For each risk factor, the first row gives the performance metrics when only the intercept was included, the second the performance metrics when *both* the intercept and fixed effects are added and so forth. The final row gives the performance metrics for the final stage 1 model. NORF: Non-outcome risk factor, DH: Demographic health, SA2: Statistical area level 2

		ALC	SR	LOOCV
Risky alcohol consumption	Intercept only	0.00	0.00	-10218.90
	Fixed effects (FE)	0.25	0.11	-9343.83
	NORF random effects (RE)	0.27	0.12	-9168.88
	DH RE	0.28	0.13	-9253.30
	SA2 RE	0.59	0.30	-9458.02
	Residual error (sd = 1)	0.62	0.35	-9891.93
	Residual error (sd = 2)	0.69	0.45	-12373.01
Inadequate diet	Intercept only	0.00	0.00	-11898.00
	FE	0.09	0.04	-11583.33
	NORF RE	0.10	0.05	-11413.50
	DH RE	0.14	0.07	-11463.79
	SA2 RE	0.52	0.23	-11670.57
	Residual error (sd = 1)	0.57	0.30	-12253.55
	Residual error (sd = 2)	0.66	0.41	-15639.11
Obese	Intercept only	0.00	0.00	-10758.03
	FE	0.26	0.10	-9914.78
	NORF RE	0.26	0.10	-9850.31
	DH RE	0.31	0.13	-9883.91
	SA2 RE	0.60	0.27	-10126.73
	Residual error (sd = 1)	0.63	0.33	-10677.55
	Residual error (sd = 2)	0.70	0.43	-13525.59
Current smoking	Intercept only	0.00	0.01	-7440.70
	FE	0.25	0.12	-6564.64
	NORF RE	0.27	0.14	-6314.59
	DH RE	0.31	0.17	-6381.14
	SA2 RE	0.56	0.32	-6626.02
	Residual error (sd = 1)	0.57	0.36	-6893.80
	Residual error (sd = 2)	0.62	0.45	-8467.00

Table 7: See description in Table 6

		ALC	SR	LOOCV
Inadequate activity (leisure)	Intercept only	0.00	0.00	-7153.47
	Fixed effects (FE)	0.16	0.06	-6799.33
	NORF random effects (RE)	0.18	0.07	-6657.59
	DH RE	0.21	0.09	-6670.94
	SA2 RE	0.47	0.22	-6864.23
	Residual error (sd = 1)	0.48	0.28	-7050.93
	Residual error (sd = 2)	0.56	0.39	-8433.78
Inadequate activity (all)	Intercept only	0.00	0.00	-7622.71
	FE	0.15	0.06	-7238.44
	NORF RE	0.17	0.06	-7101.44
	DH RE	0.20	0.08	-7114.28
	SA2 RE	0.45	0.21	-7293.53
	Residual error (sd = 1)	0.47	0.27	-7487.59
	Residual error (sd = 2)	0.55	0.37	-8983.52
Overweight/ obese	Intercept only	0.00	0.00	-11030.30
	FE	0.21	0.12	-9929.64
	NORF RE	0.21	0.13	-9893.96
	DH RE	0.24	0.15	-9931.15
	SA2 RE	0.49	0.26	-10128.58
	Residual error (sd = 1)	0.52	0.32	-10521.22
	Residual error (sd = 2)	0.61	0.42	-13075.34
Risky waist circumference	Intercept only	0.00	0.00	-10503.49
	FE	0.26	0.13	-9400.15
	NORF RE	0.26	0.14	-9354.67
	DH RE	0.29	0.16	-9388.27
	SA2 RE	0.60	0.30	-9617.15
	Residual error (sd = 1)	0.62	0.36	-9983.51
	Residual error (sd = 2)	0.70	0.46	-12439.92

Table 8: The progression of the performance metrics (given in Section E.2) as components of the linear predictor for the stage 2 model are included. For each risk factor, the first row gives the performance metrics when only the intercept was included, the second the performance metrics when *both* the intercept and fixed effects are added and so forth. The final row gives the performance metrics for the stage 2 model used to derive the results given in the main paper. Both the mean absolute relative bias (MARB) and mean relative root mean squared error (MRRMSE) are given as $\times 100$. SA2: Statistical area level 2, SA3: Statistical area level 3, SA4: Statistical area level 4

		SA4			Major-by-state		
		MARB	MRRMSE	MIOP	MARB	MRRMSE	MIOP
Risky alcohol consumption	Intercept only	24.95	25.17	0.64	8.81	9.31	0.60
	Fixed effects (FE) (non-varying)	22.62	23.37	0.64	6.76	7.62	0.53
	FE (varying)	13.98	16.10	0.74	5.53	7.06	0.65
	External latent field	13.24	15.50	0.76	4.66	6.51	0.73
	SA2 random effect (RE)	13.22	15.54	0.76	4.60	6.54	0.72
	SA3 RE	13.19	15.61	0.76	4.59	6.55	0.72
	Benchmarking	12.88	14.86	0.79	1.25	2.37	1.00
Inadequate diet	Intercept only	10.02	10.18	0.81	3.38	3.73	0.75
	FE (non-varying)	9.50	10.03	0.81	3.56	4.38	0.71
	FE (varying)	8.92	10.31	0.76	2.74	4.06	0.79
	External latent field	8.92	10.40	0.76	2.76	4.14	0.82
	SA2 RE	8.92	10.44	0.76	2.74	4.20	0.82
	SA3 RE	8.89	10.46	0.77	2.72	4.18	0.83
	Benchmarking	8.82	9.94	0.81	1.14	1.77	1.00
Obese	Intercept only	23.63	23.76	0.55	12.16	12.44	0.23
	FE (non-varying)	16.45	17.20	0.67	5.71	6.47	0.65
	FE (varying)	13.59	15.48	0.78	5.75	6.93	0.58
	External latent field	13.60	15.68	0.78	5.64	6.89	0.59
	SA2 RE	13.51	15.67	0.78	5.52	6.86	0.61
	SA3 RE	13.45	15.69	0.79	5.47	6.82	0.62
	Benchmarking	12.42	14.58	0.80	1.17	2.16	1.00
Current smoking	Intercept only	42.22	42.61	0.65	18.06	18.41	0.36
	FE (non-varying)	26.23	27.86	0.78	8.19	9.71	0.68
	FE (varying)	25.70	29.12	0.75	7.37	9.81	0.67
	External latent field	25.55	29.14	0.75	7.22	9.82	0.69
	SA2 RE	25.49	29.35	0.75	7.03	9.85	0.71
	SA3 RE	25.43	29.41	0.75	7.00	9.81	0.71
	Benchmarking	25.45	28.64	0.78	1.91	3.59	1.00

Table 9: See description in Table 9

		SA4			Major-by-state		
		MARB	MRRMSE	MIOP	MARB	MRRMSE	MIOP
Inadequate activity (leisure)	Intercept only	4.32	4.41	0.71	1.72	1.85	0.66
	Fixed effects (FE) (non-varying)	3.72	3.95	0.77	1.01	1.38	0.83
	FE (varying)	3.23	3.85	0.79	1.23	1.68	0.75
	External latent field	3.21	3.87	0.78	1.24	1.71	0.73
	SA2 random effect (RE)	3.20	3.88	0.79	1.23	1.72	0.71
	SA3 RE	3.19	3.89	0.77	1.22	1.72	0.73
	Benchmarking	3.10	3.62	0.81	0.27	0.58	1.00
Inadequate activity (all)	Intercept only	4.19	4.27	0.78	1.52	1.71	0.73
	FE (non-varying)	3.76	4.01	0.80	1.12	1.49	0.83
	FE (varying)	3.33	3.99	0.80	1.09	1.64	0.76
	External latent field	3.34	4.03	0.81	1.08	1.66	0.78
	SA2 RE	3.34	4.04	0.80	1.08	1.68	0.78
	SA3 RE	3.33	4.05	0.80	1.06	1.68	0.78
	Benchmarking	3.17	3.71	0.84	0.27	0.62	1.00
Overweight/ obese	Intercept only	8.63	8.72	0.73	5.23	5.34	0.34
	FE (non-varying)	6.15	6.54	0.76	2.45	2.88	0.62
	FE (varying)	5.13	6.12	0.83	2.49	3.12	0.60
	External latent field	5.00	6.08	0.84	2.21	3.00	0.70
	SA2 RE	4.99	6.09	0.84	2.16	3.00	0.72
	SA3 RE	4.97	6.12	0.84	2.15	3.00	0.72
	Benchmarking	4.83	5.81	0.88	0.65	1.13	1.00
Risky waist circumference	Intercept only	11.48	11.57	0.58	6.24	6.36	0.29
	FE (non-varying)	9.29	9.62	0.70	4.00	4.34	0.44
	FE (varying)	7.80	8.72	0.70	4.35	4.83	0.43
	External latent field	7.68	8.70	0.71	4.33	4.90	0.52
	SA2 RE	7.61	8.69	0.72	4.25	4.85	0.53
	SA3 RE	7.58	8.71	0.70	4.27	4.88	0.53
	Benchmarking	7.32	8.55	0.68	1.20	1.61	1.00

F Additional results

F.1 Smoking

Similar to previous work in Australia [75], the prevalence of smoking shows strong spatial patterns (Figs. 13 to 15), with generally lower rates in major cities and less disadvantaged areas. That said, there are several instances where an area classed as a major city shows substantively high rates of smoking. These are predominately most disadvantaged areas in WA, ACT and NSW; some of which are industrial areas with small populations. The median of the point estimates for all very remote areas (0.33) is around 126% higher than that for the non-very remote areas (0.14), with 75% higher median CVs (30.4% compared to 17.3%). By improving the reach, we garner insights about these disadvantaged areas, albeit with higher entropy.

F.2 Alcohol

The prevalence of risky alcohol consumption shows strong spatial patterns (Figs. 16 to 18). The results suggest that less disadvantaged areas have higher proportions of risky alcohol consumption, which generally manifests in higher prevalence in major cities. This is supported by other Australian surveys [1, 13] and previous research [76, 17]. The effect of the inverse relationship between higher prevalence of risky alcohol consumption and lower socioeconomic disadvantage is mirrored in Figure 10 in the main paper, where the modelled estimate for the non-benchmarked areas is lower than the direct estimate.

Although the figures suggests lower than average prevalence in more disadvantaged and remote areas (e.g. the middle of Australia), the estimates for these very remote regions (such as the Northern Territory) have greater uncertainty and thus these are not substantially different to the national average (see the Additional File). Furthermore, unlike other risk factors where prevalence estimates exhibit relative homogeneity within SES and remoteness groups (see Fig. 7), for risky alcohol consumption the estimates exhibit far greater heterogeneity for more disadvantaged areas in major cities.

F.3 Diet

Unlike the other risk factors, inadequate diet (Figs. 19 to 21) tends to exhibit less dependence with SES and remoteness, which agrees with the broad findings from other Australian surveys [1, 11] (see Section A). As a result, inadequate diet was one of the only risk factors for which the modelled estimate for the non-benchmarked areas was lower than the direct estimate. Unlike most of the other risk factor measures where healthy behaviour was generally reserved for major cities, areas with low prevalence of inadequate diet can be found outside major cities. For example some areas in Northern Queensland, Western New South Wales and Western Australia exhibit lower prevalence than the national average. This is supported by the new estimates for very remote areas. The median point estimates for these areas are the same as that for non-very remote areas, albeit with over three times the uncertainty (using CV).

F.4 Weight

The three weight measures (overweight/obese, obese and risky waist circumference) exhibit significant spatial variation with lower proportions in less disadvantaged and urban areas (Figs. 22 to 30). Across all three measures, the prevalence was very strongly tied to remoteness with substantially lower prevalence almost exclusively occurring in major cities. Furthermore, the most notable differences in patterns between the estimates for obese and overweight/obese are found in major cities.

Some low proportions for both obese and overweight/obese are found in the Northern Territory, which is largely composed of very remote and more disadvantaged areas. However, these areas have a high level of uncertainty and thus provide insufficient evidence of a meaningful difference. Across the three weight measures, the uncertainty (using CV) of estimates for very remote areas are, on average, over 2.5 times those for non-very remote areas.

F.5 Physical activity

Similar to our other modelled risk factors and other Australian research [77], both activity variables (Figs. 31 to 36) exhibit high spatial variation, with lower prevalence of inadequate activity in major cities and least disadvantaged areas. One novelty of this work is estimates for inadequate activity (all) as well as inadequate activity (leisure). The most notable difference between the two was in non-remote areas.

By improving the reach of the SHAA, the modelled risk factors provide insights into the spatial disparities of physical activity in very remote areas of Australia. These areas have, on average, 7% higher prevalence but also over 2.5 times the uncertainty (using CV), compared with non-very remote areas.

G Additional plots

In this section, we provide supplemental plots and maps to those provided in the main paper.

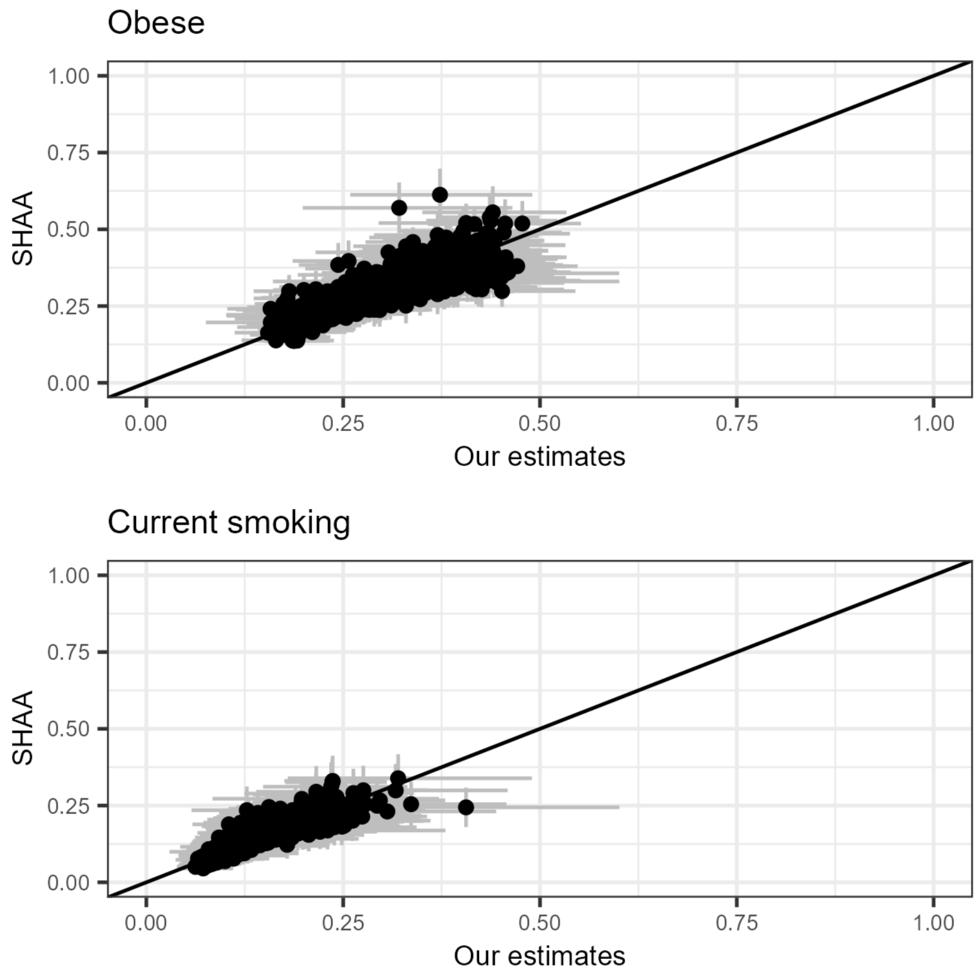

Figure 2: Scatter plots for obese and current smoking, where the posterior median PHA-level prevalence estimates from this work (x-axis) are compared to the corresponding PHA-level estimates from the SHAA (y-axis). Each point also has a 95% highest density interval and 95% confidence interval from this work and the SHAA, respectively. The black diagonal line represents when the two axis are equal. Note that the definitions used for our estimates and those from the SHAA are similar but not identical. Furthermore, we report proportions, while the SHAA reports age-standardised rates which have been converted to proportions for comparison.

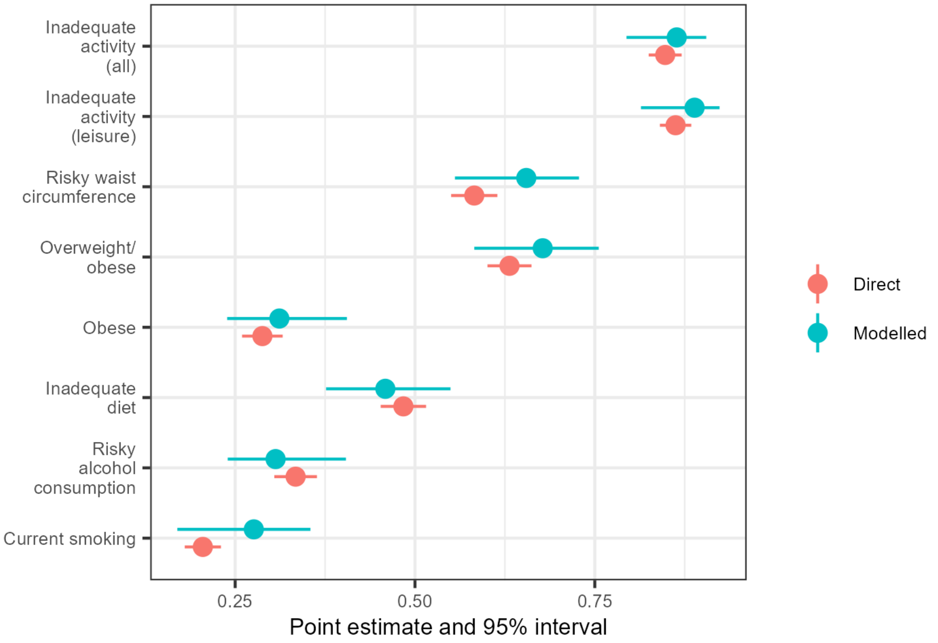

Figure 3: Comparison of the aggregated direct and modelled estimate for the non-benchmarked areas. For the modelled points, the posterior median and 95% highest posterior density interval (HPDI) is used, while for the direct estimates the direct estimate and 95% confidence interval is used.

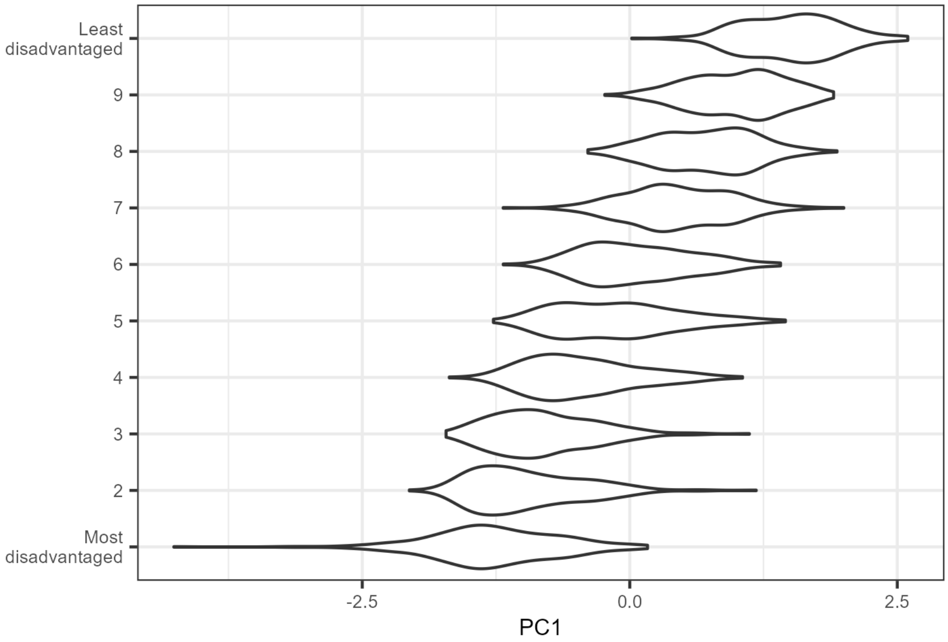

Figure 4: Distribution of principal component 1 (PC1) by SES index quintiles (IRSD).

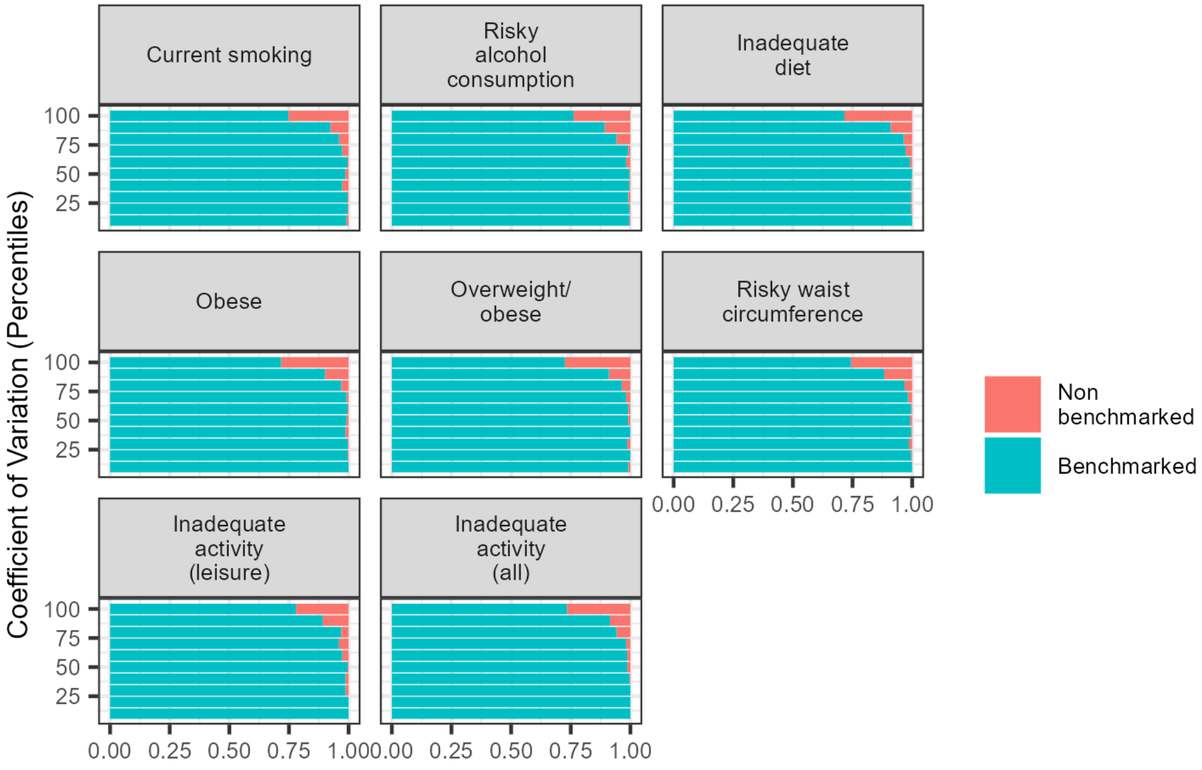

Figure 5: Distribution of coefficients of variation (CV) of the posterior prevalence estimates by risk factor and whether the area was benchmarked. The CVs are represented in percentiles with higher values denoting higher CVs and thus more uncertainty. The x-axis represents the proportion of the areas in each percentile that were benchmarked.

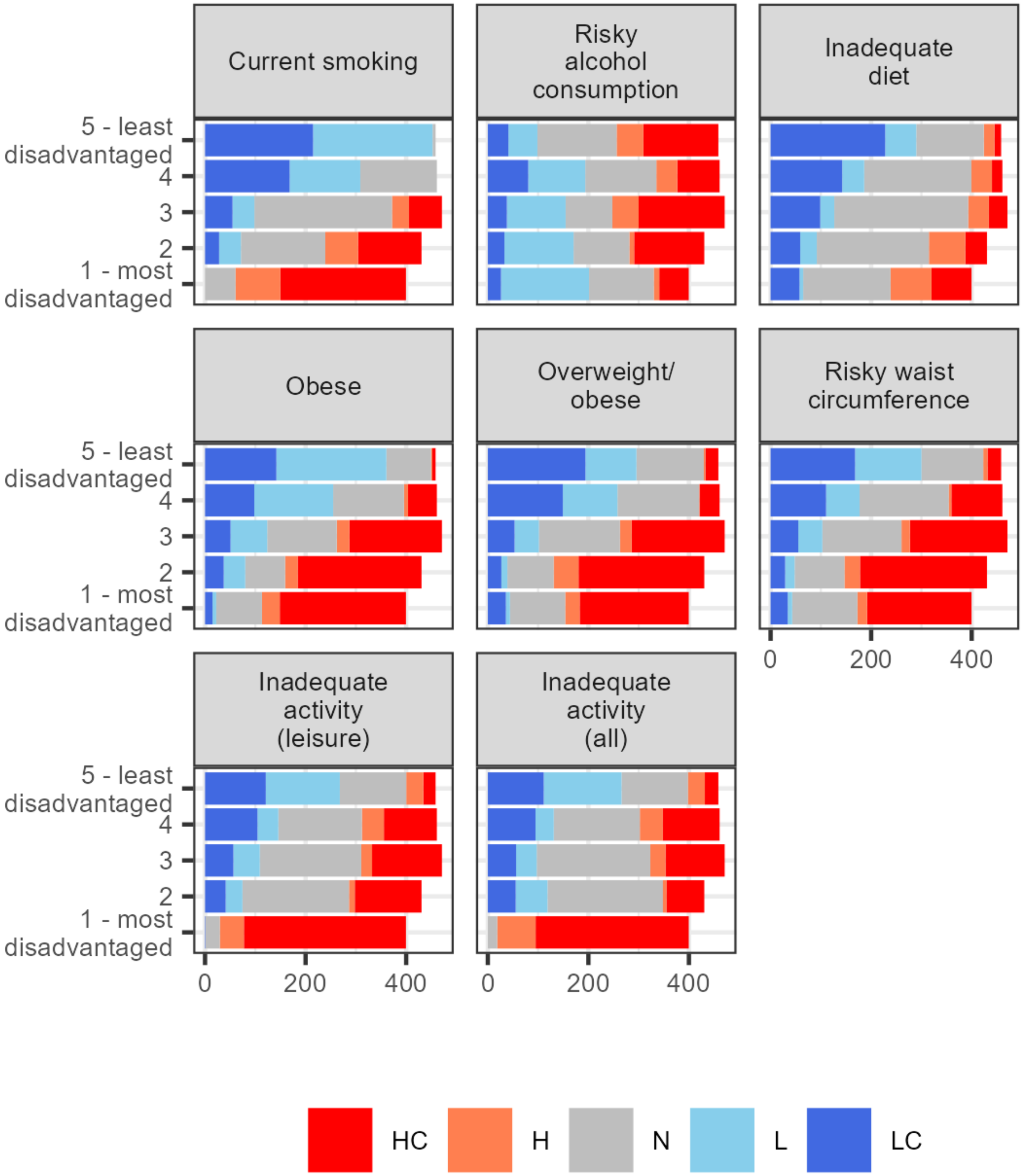

Figure 6: Distribution of the evidence classifications (HC, H, L, and LC) by SES index quintile (IRSD) and risk factor. The x-axis is the weighted number of SA2s using the 2017-18 ERP as weights.

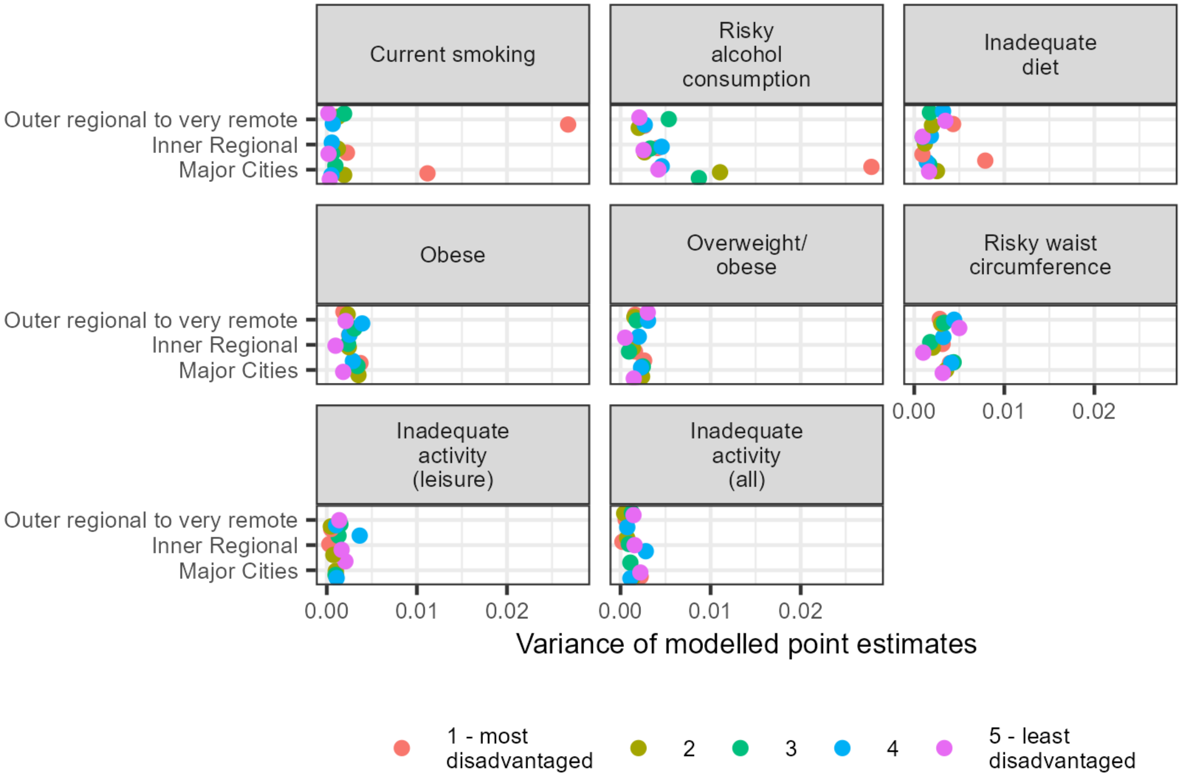

Figure 7: Summary of the variance of modelled point estimates (posterior medians) by SES index quintiles (IRSD), remoteness and risk factor. Points have been randomly jittered along the y-axis to avoid overlap. Each point represents the variance of all the point estimates for the specific risk factor, SES index quintile and remoteness category.

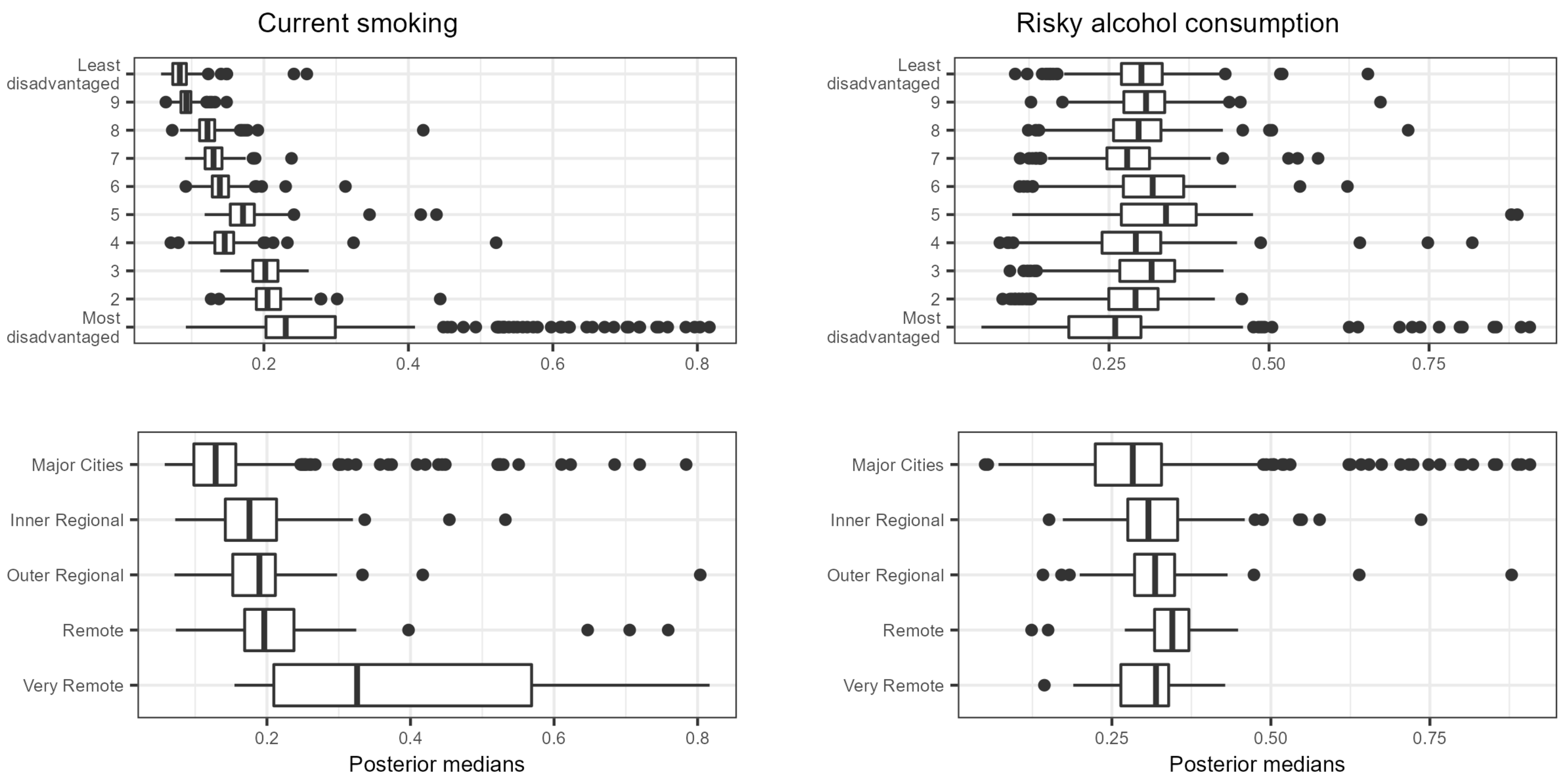

(a) **(b)**

Figure 8: Boxplots summarising the distribution of the modelled point estimates (posterior medians) by the SES index and remoteness.

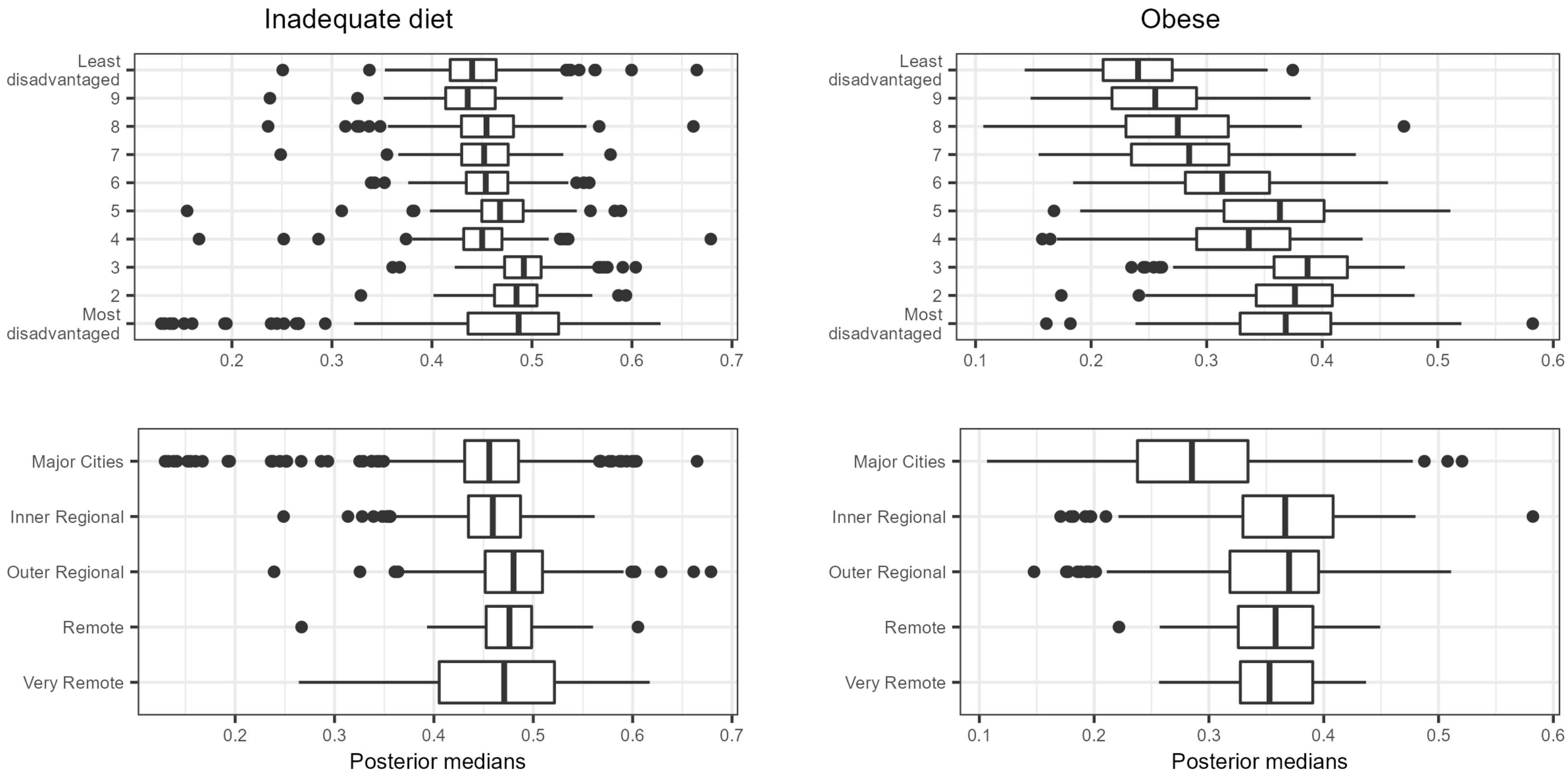

(a) **(b)**

Figure 9: Boxplots summarising the distribution of the modelled point estimates (posterior medians) by the SES index and remoteness.

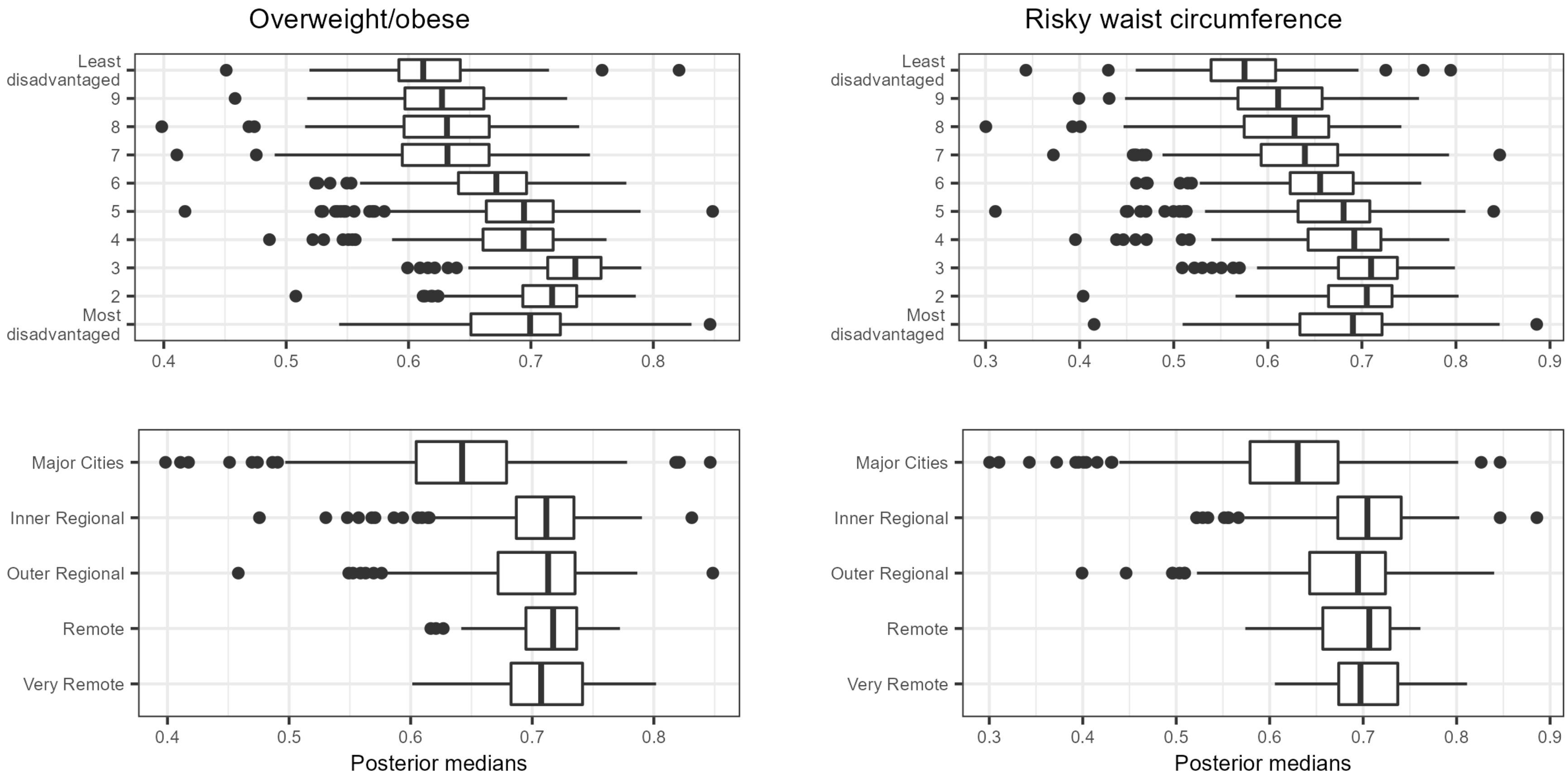

(a)

(b)

Figure 10: Boxplots summarising the distribution of the modelled point estimates (posterior medians) by the SES index and remoteness.

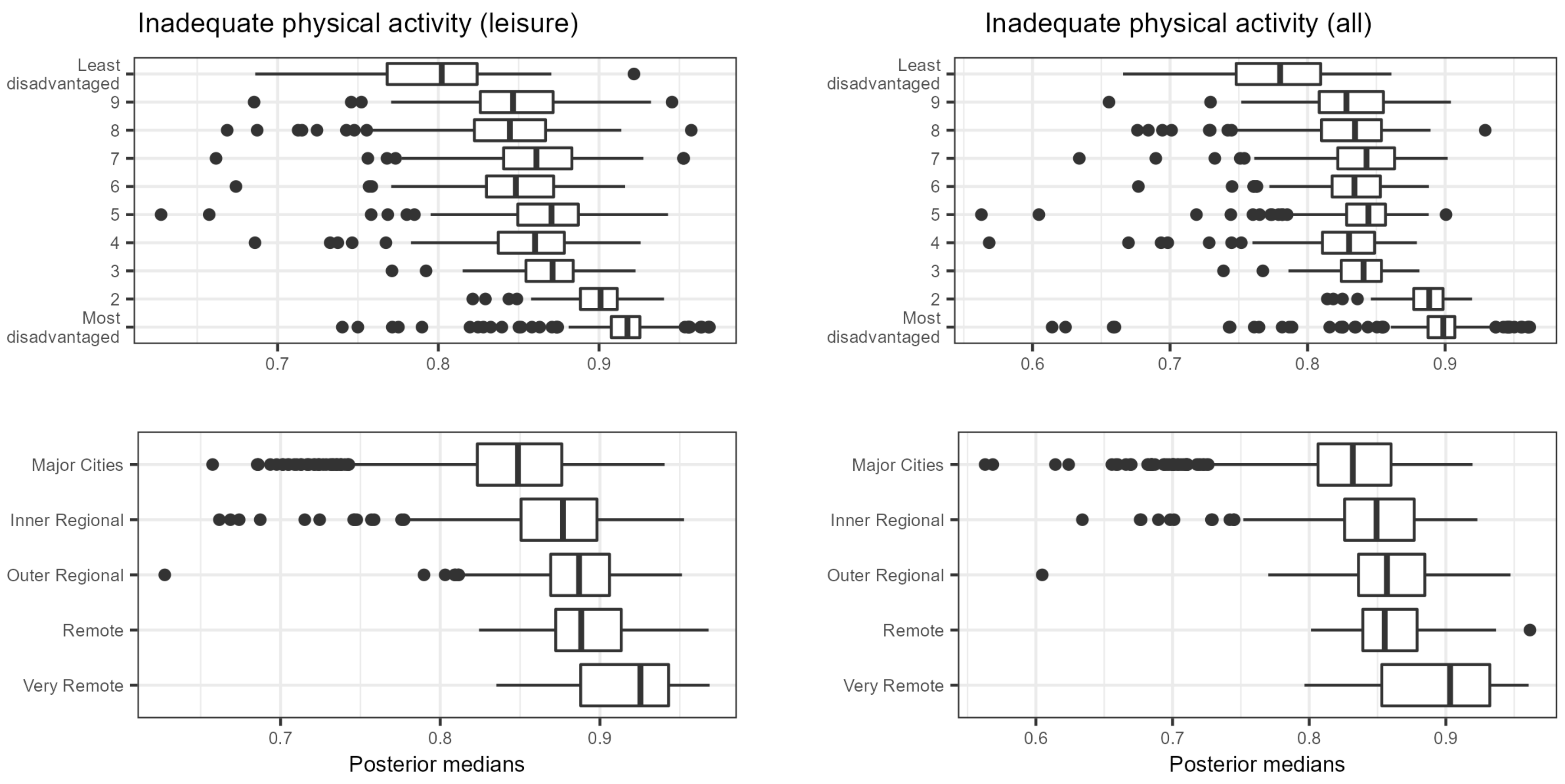

(a) **(b)**

Figure 11: Boxplots summarising the distribution of the modelled point estimates (posterior medians) by the SES index and remoteness.

H Additional maps

H.1 Socioeconomic status

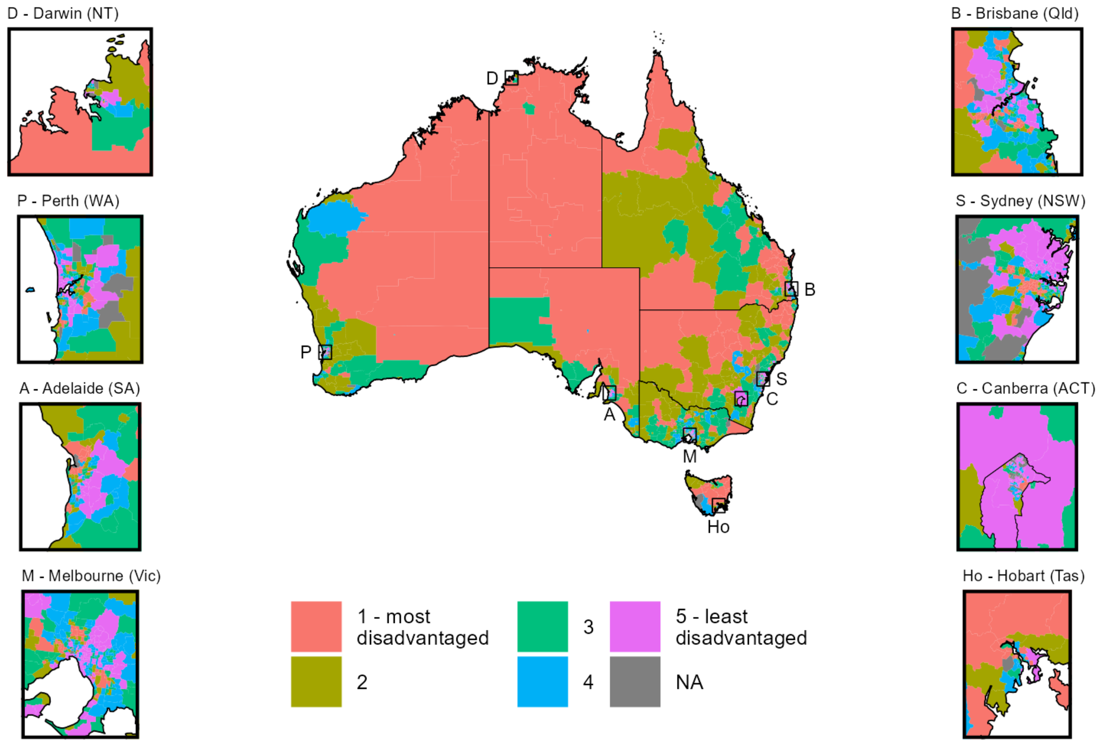

Figure 12: Choropleth maps displaying quintiles of the SES index for 2221 SA2s across Australia. The map includes insets for the eight capital cities for each state and territory, with black boxes on the main map indicating each insets' respective location. Gray areas were excluded from estimation due to the exclusion criteria described in Section 2.1 of the main paper. Black lines represent the boundaries of the eight states and territories of Australia.

H.2 Current smoking

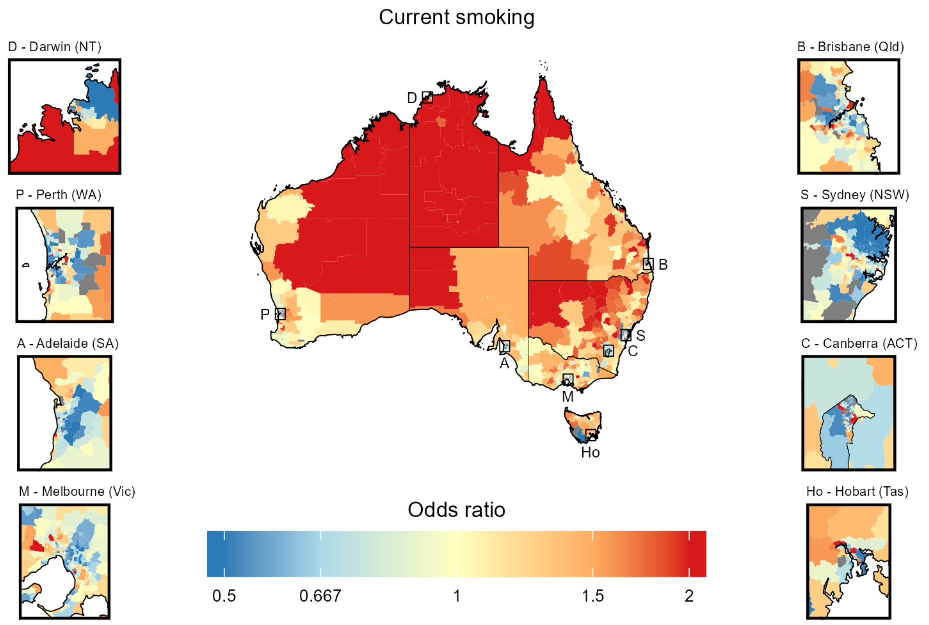

Figure 13: Choropleth maps displaying the modelled odds ratios (OR) for 2221 SA2s in Australia. ORs above 1 indicate that the prevalence is higher than the national average. The map includes insets for the eight capital cities for each state and territory, with black boxes on the main map indicating each insets' respective location. Note that some values are lower (or higher) than the range of color scales shown; for these values, the lowest (or highest) color is shown. White areas were excluded from estimation due to the exclusion criteria described in Section 2.1 of the main paper. Black lines represent the boundaries of the eight states and territories of Australia.

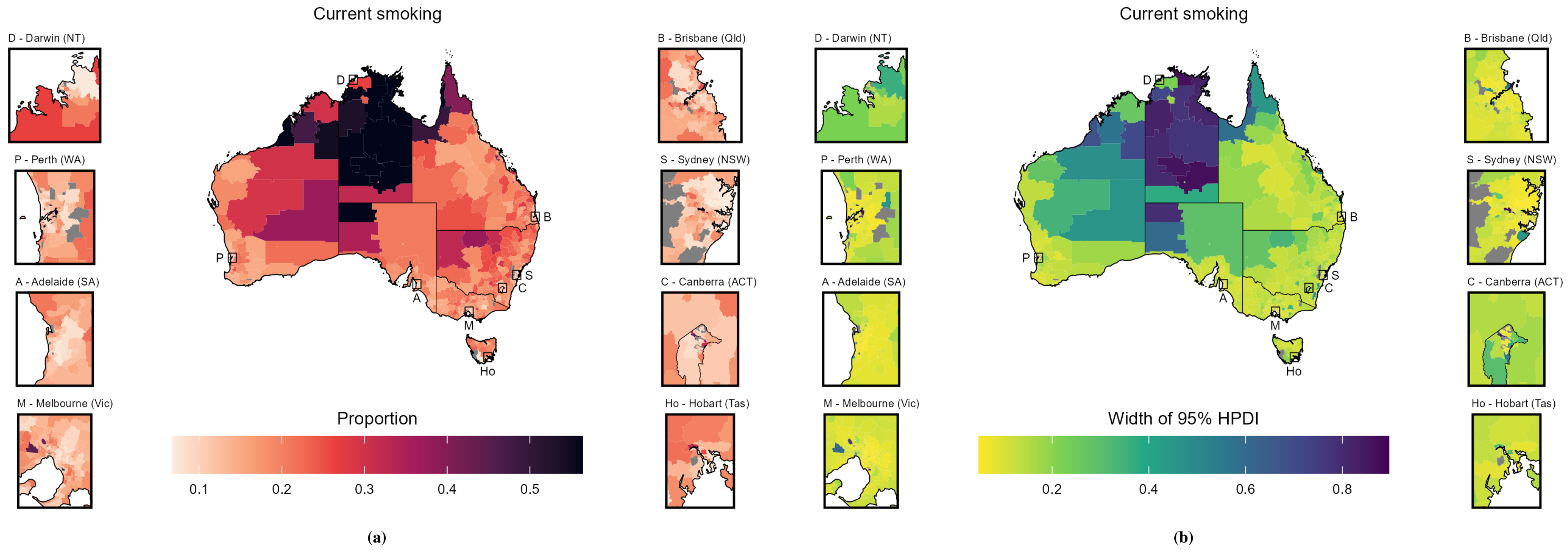

Figure 14: Choropleth maps displaying the modelled proportion (a) and width of the 95% HPDIs (b) for 2221 SA2s across Australia. The map includes insets for the eight capital cities for each state and territory, with black boxes on the main map indicating each insets' respective location. Note that prevalence values above the 99th and below the 1th percentile were assigned to the highest and lowest color, respectively. Gray areas were excluded from estimation due to the exclusion criteria described in the main paper. Black lines represent the boundaries of the eight states and territories of Australia.

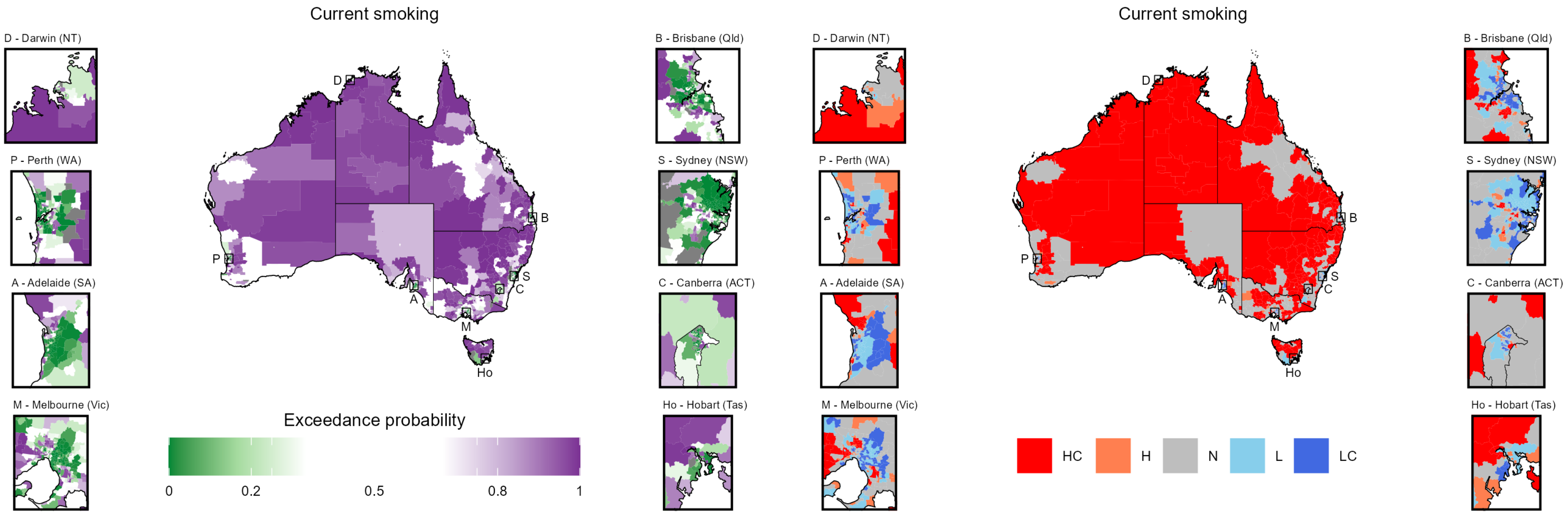

(a) (b)

Figure 15: Choropleth maps displaying the exceedance probabilities (a) and evidence classifications (b) for 2221 SA2s across Australia. The map includes insets for the eight capital cities for each state and territory, with black boxes on the main map indicating each insets' respective location. Gray areas were either excluded from estimation due to the exclusion criteria described in the main paper or were not classified according to one of the four categories. Black lines represent the boundaries of the eight states and territories of Australia.

H.3 Risky alcohol consumption

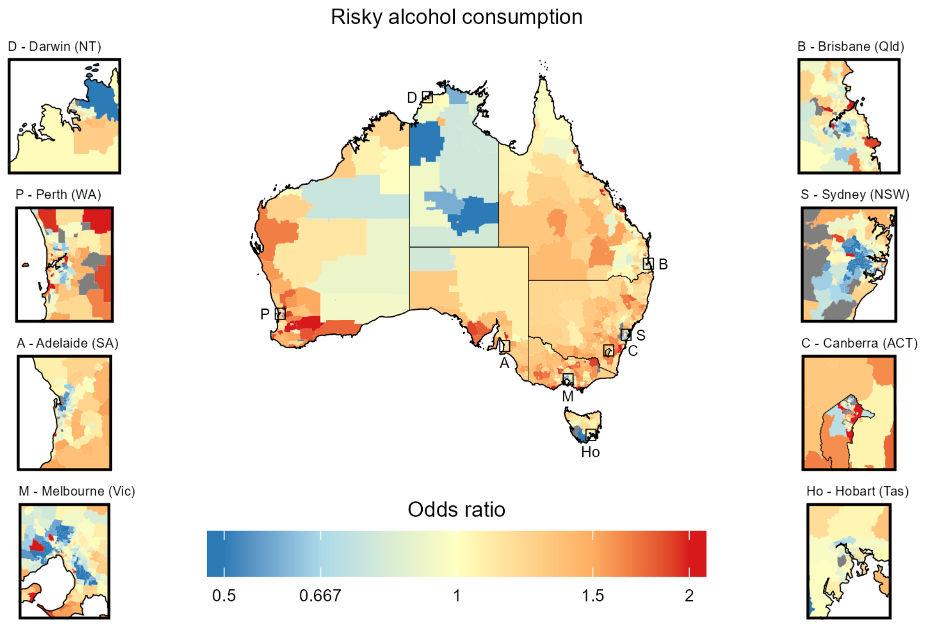

Figure 16: See caption for Fig. 13

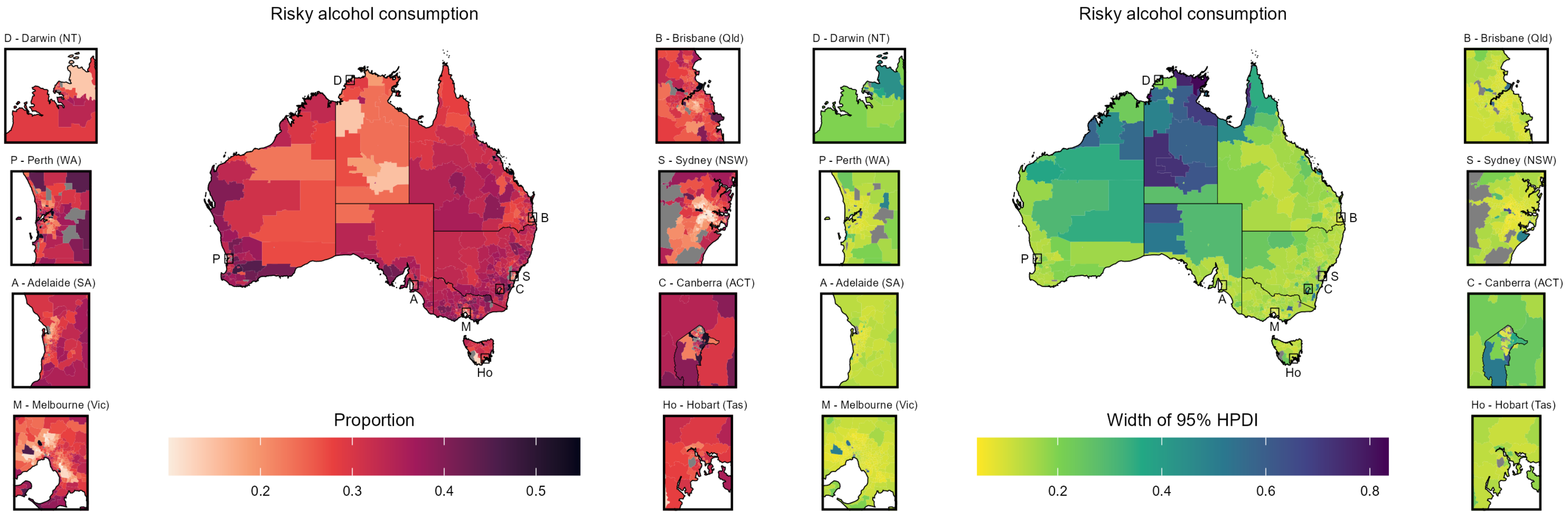

(a) (b)

Figure 17: See caption for Fig. 14

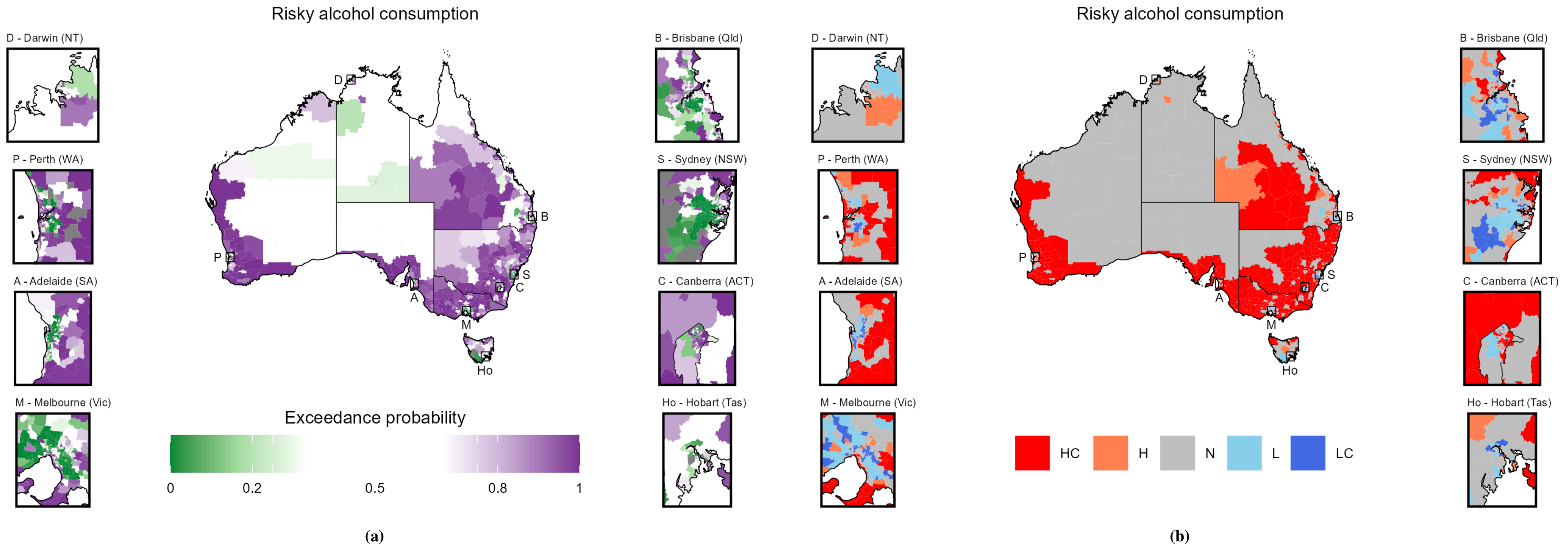

Figure 18: See caption for Fig. 15

H.4 Inadequate diet

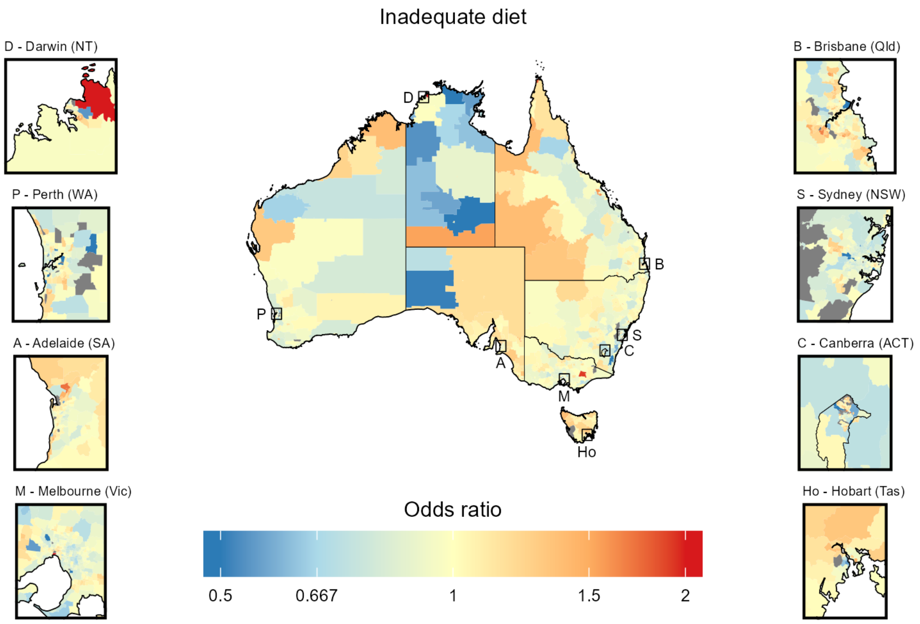

Figure 19: See caption for Fig. 13

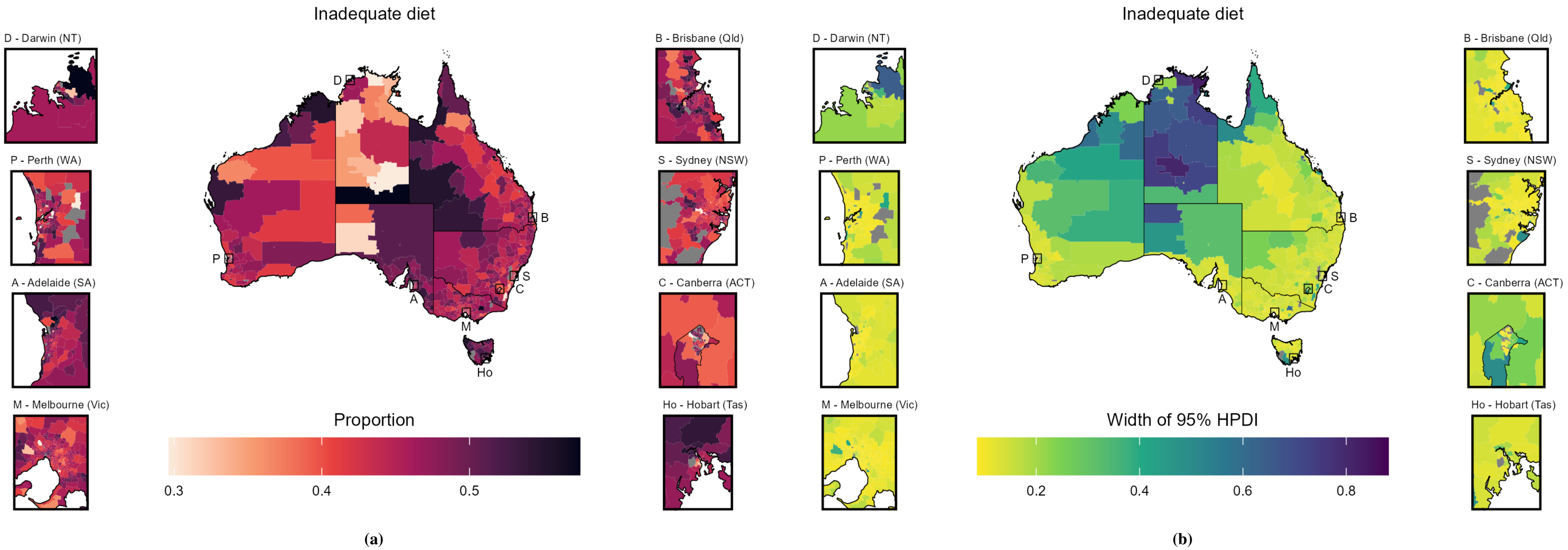

Figure 20: See caption for Fig. 14.

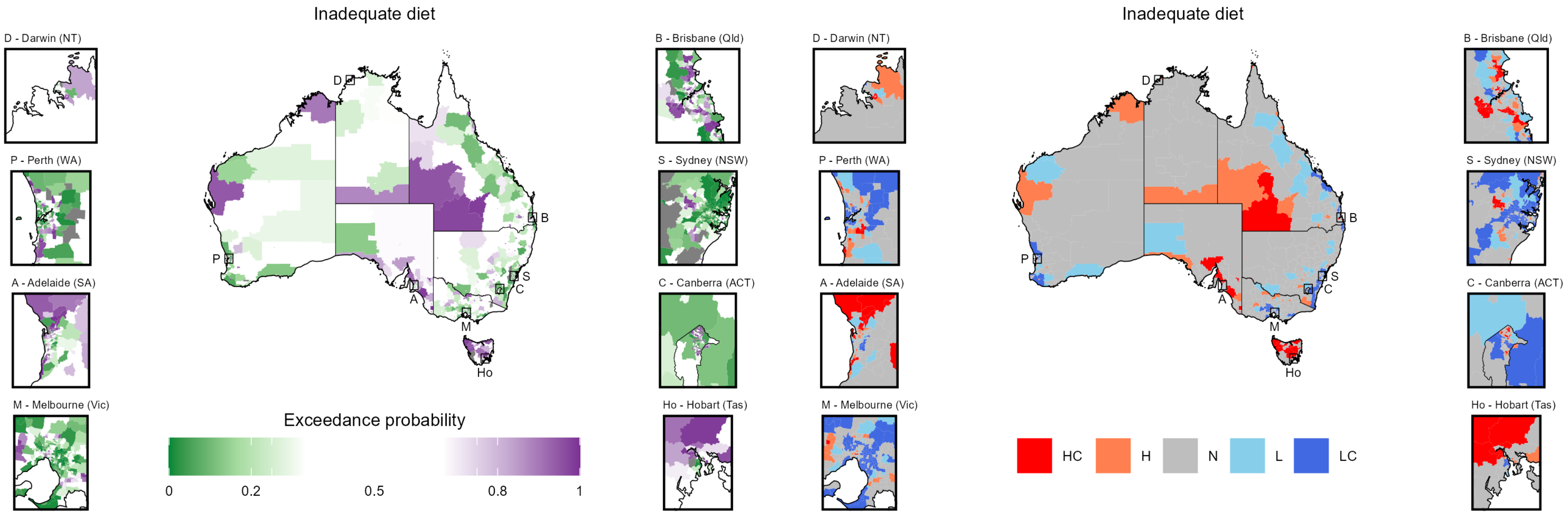

(a)

(b)

Figure 21: See caption for Fig. 15.

H.5 Obese

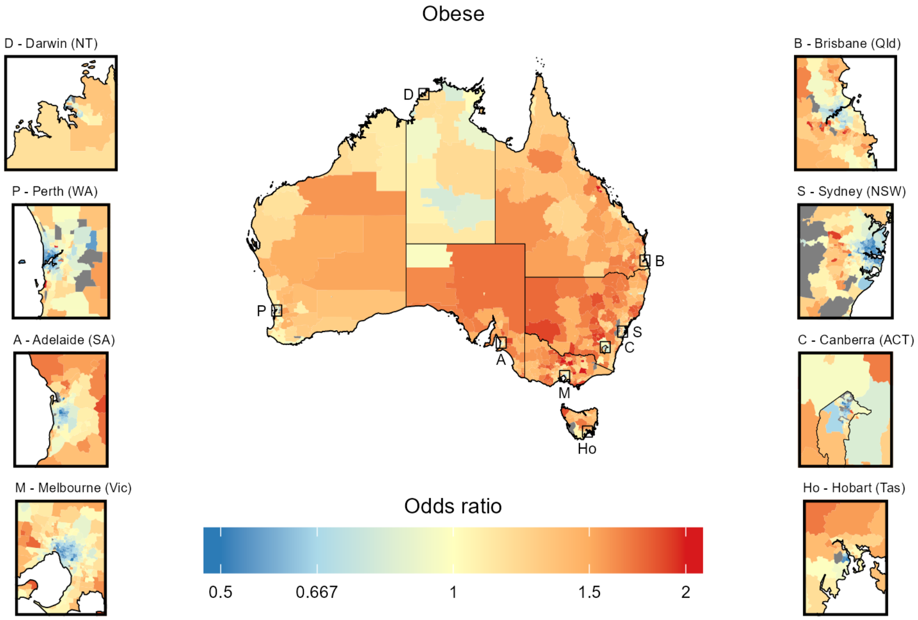

Figure 22: See caption for Fig. 13

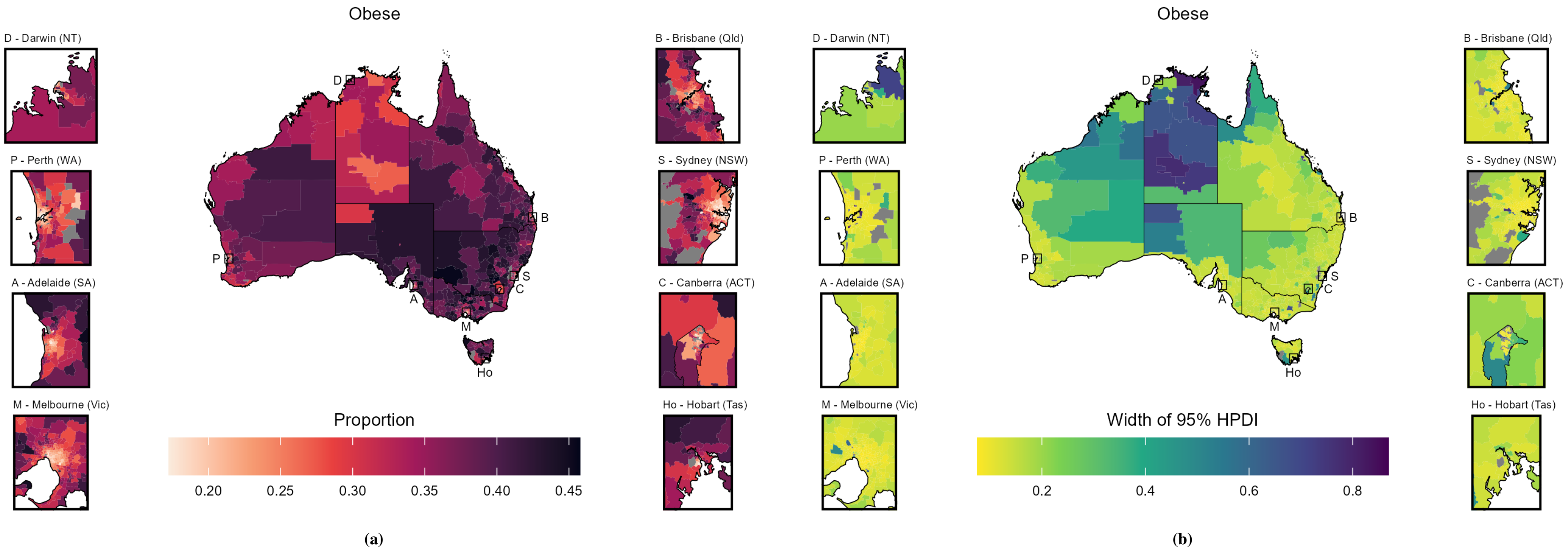

Figure 23: See caption for Fig. 14.

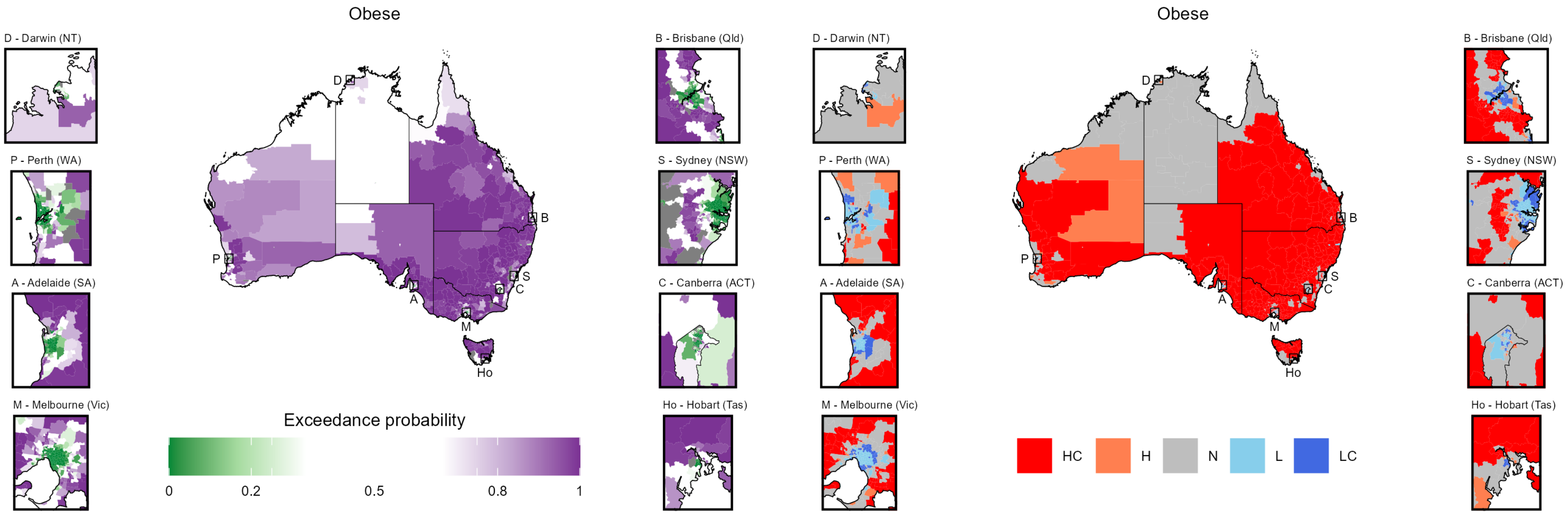

(a) (b)

Figure 24: See caption for Fig. 15.

H.6 Overweight

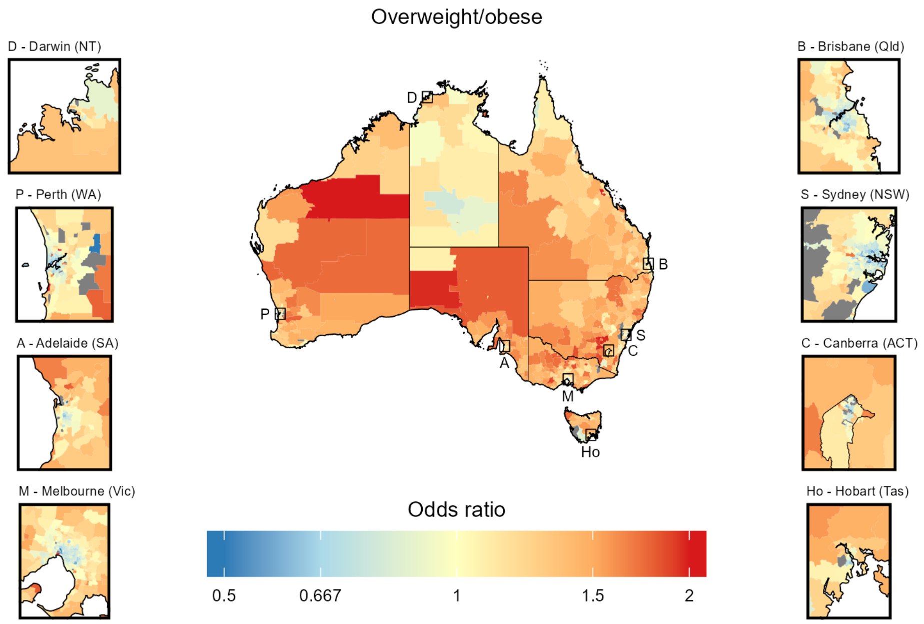

Figure 25: See caption for Fig. 13

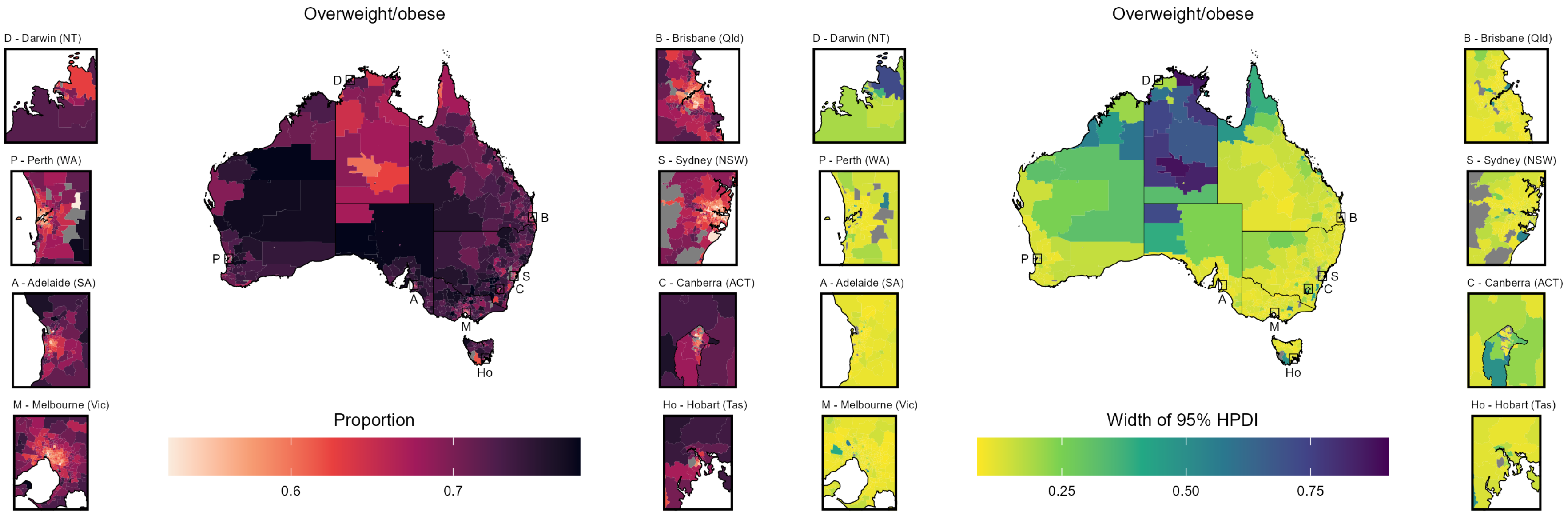

(a) (b)

Figure 26: See caption for Fig. 14.

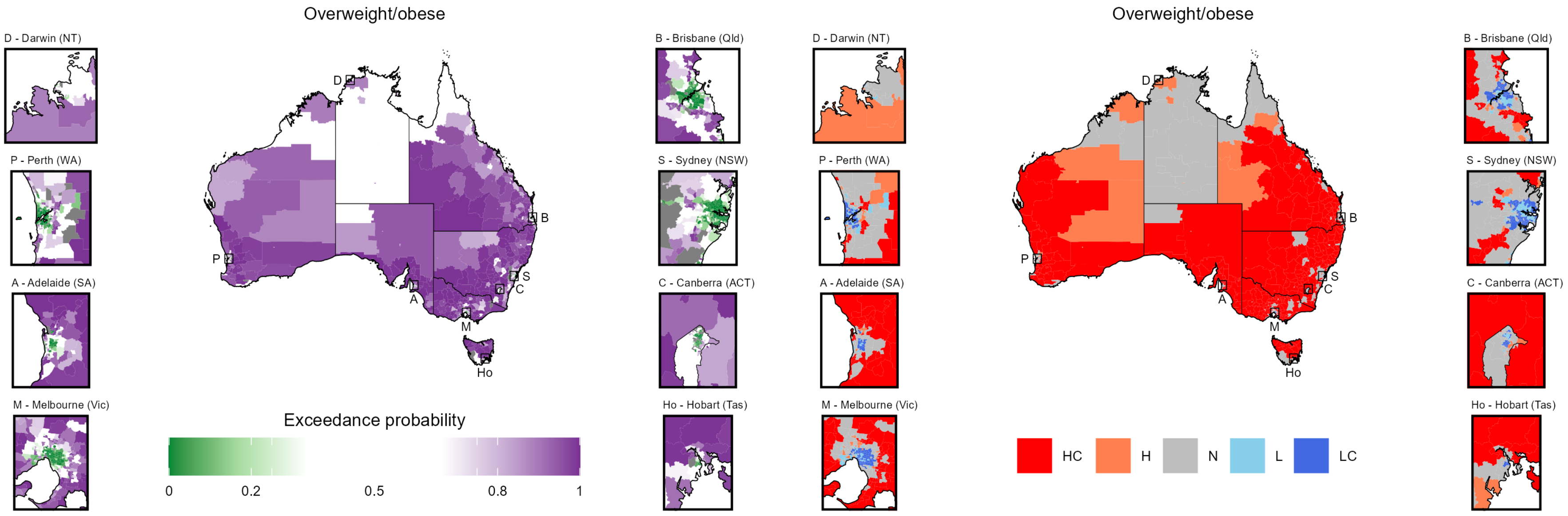

(a) (b)

Figure 27: See caption for Fig. 15.

H.7 Risky waist circumference

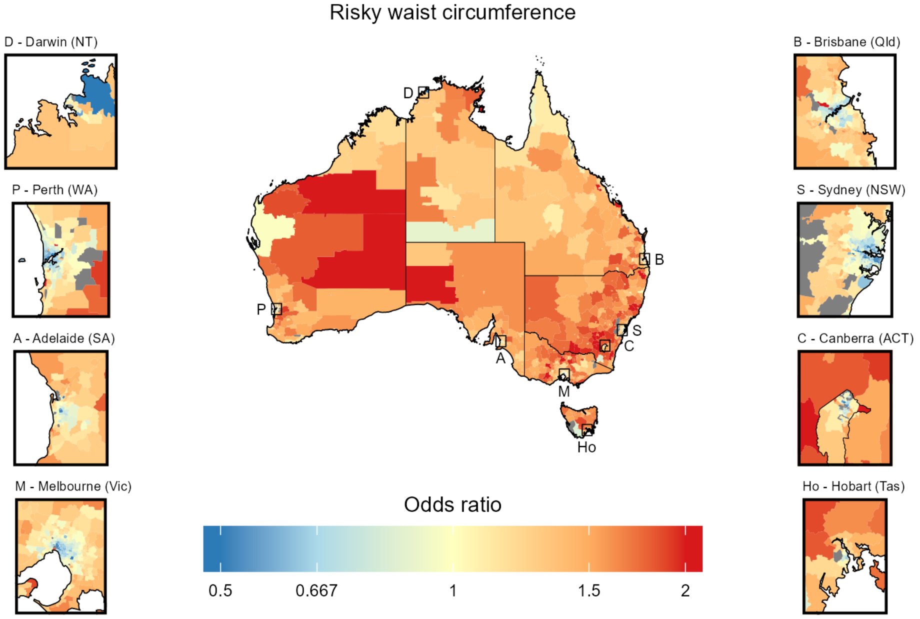

Figure 28: See caption for Fig. 13

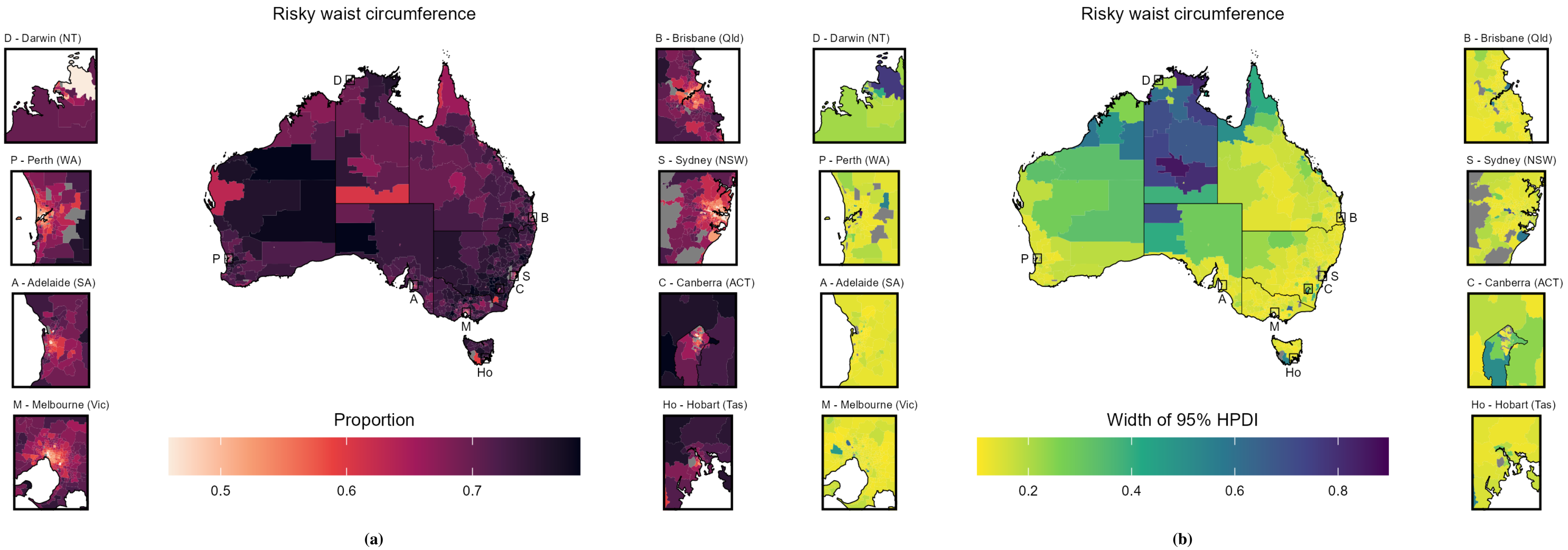

Figure 29: See caption for Fig. 14.

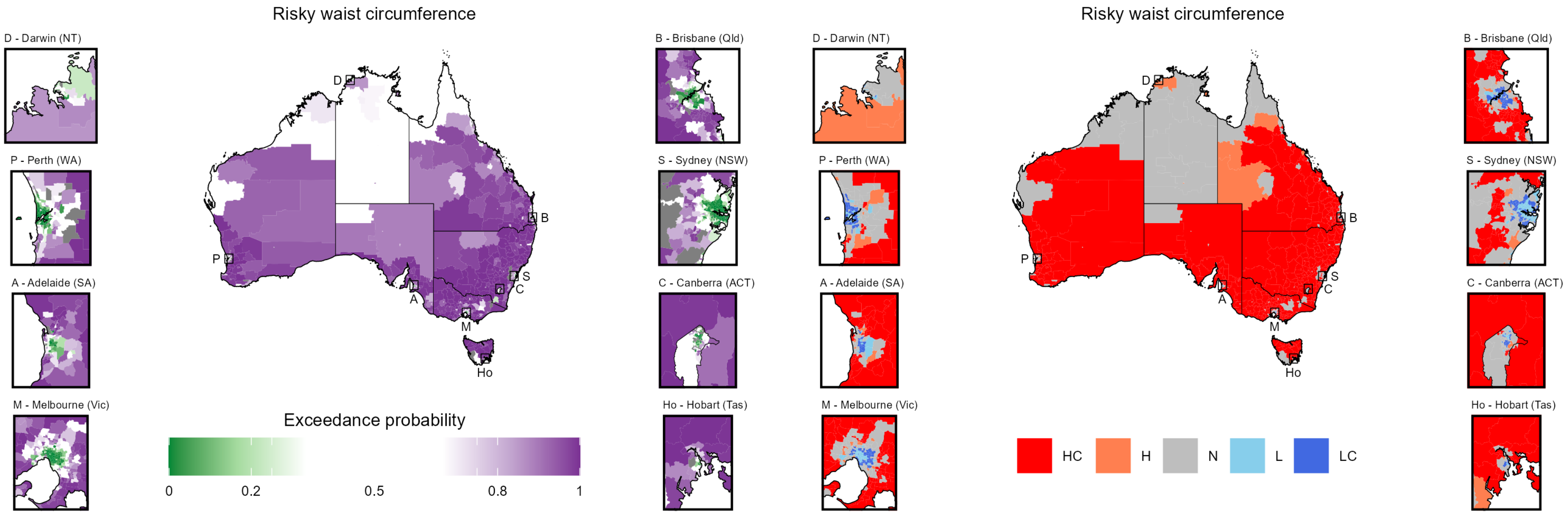

(a) (b)

Figure 30: See caption for Fig. 15.

H.8 Inadequate activity (leisure)

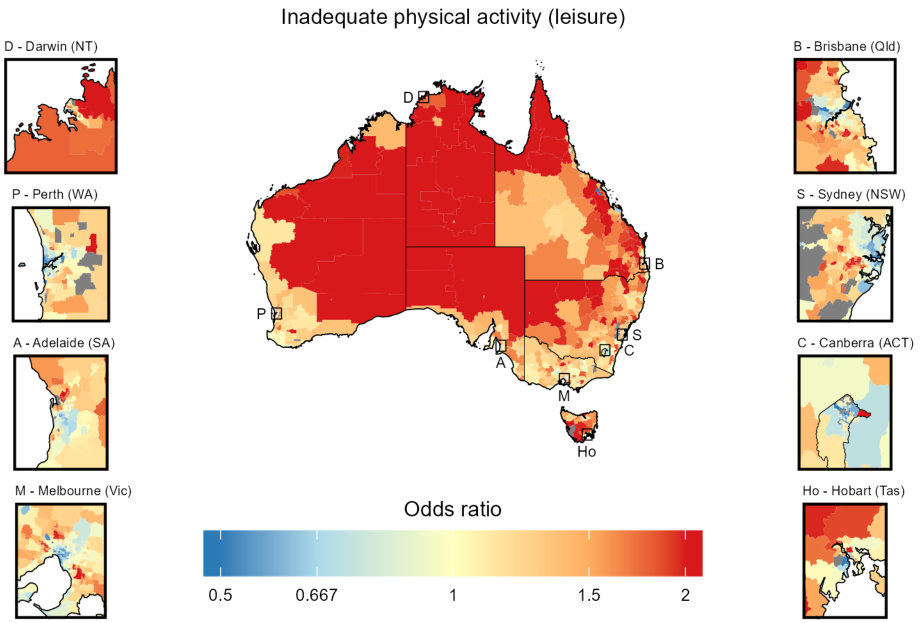

Figure 31: See caption for Fig. 13

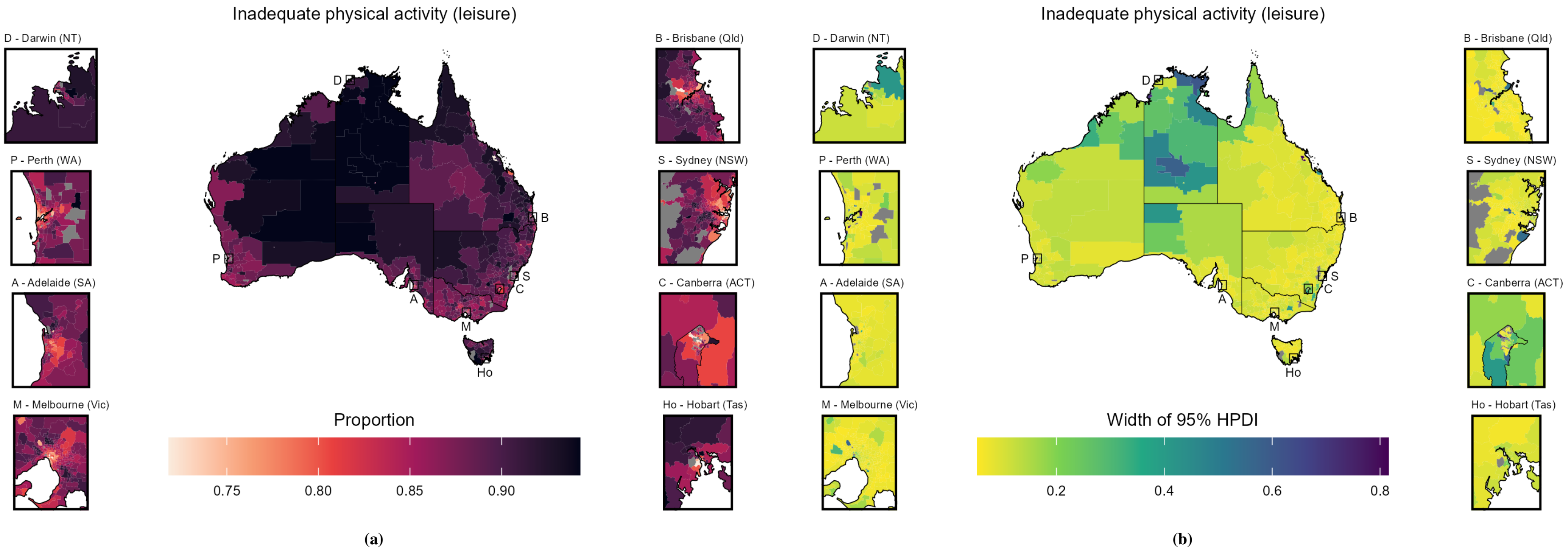

Figure 32: See caption for Fig. 14.

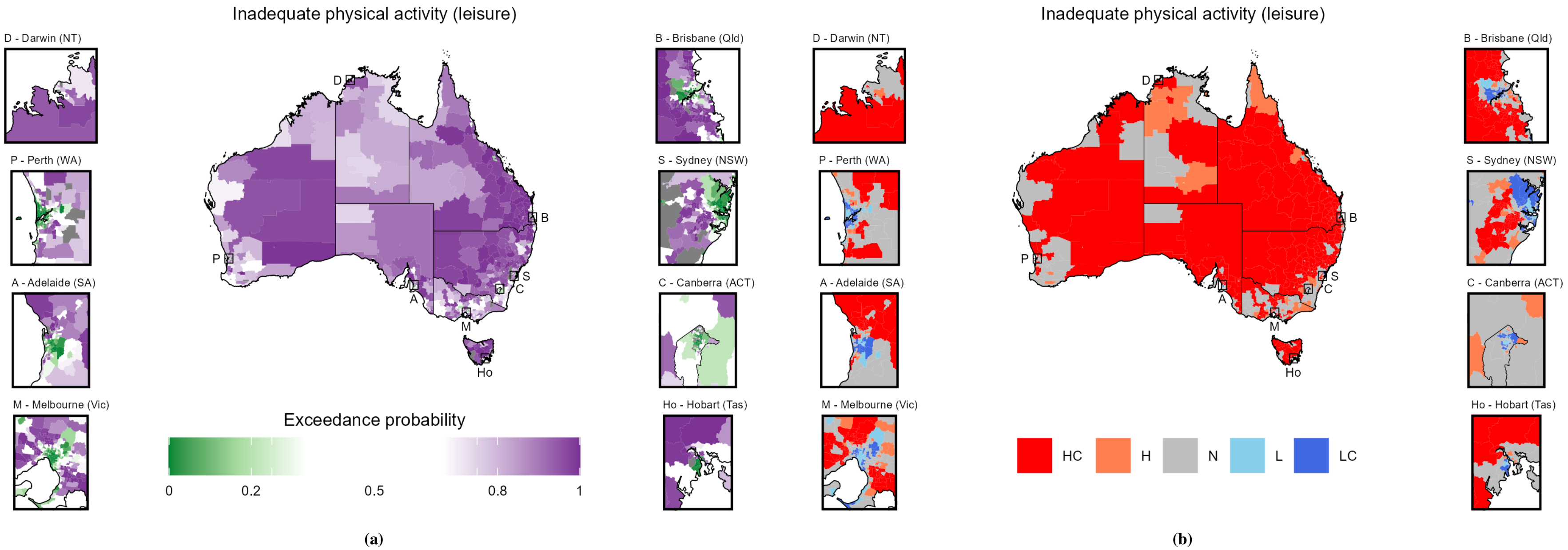

Figure 33: See caption for Fig. 15.

H.9 Inadequate activity (all)

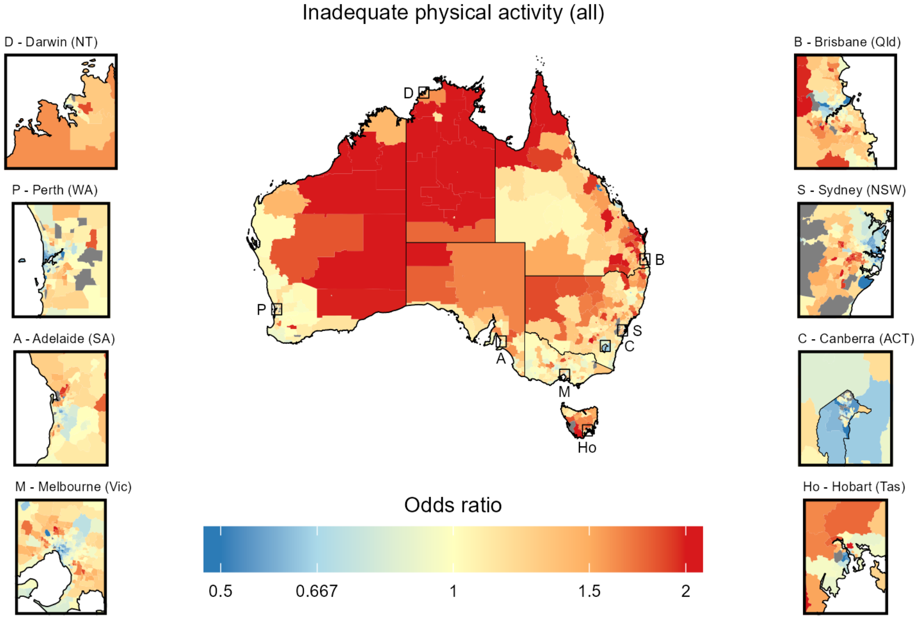

Figure 34: See caption for Fig. 13

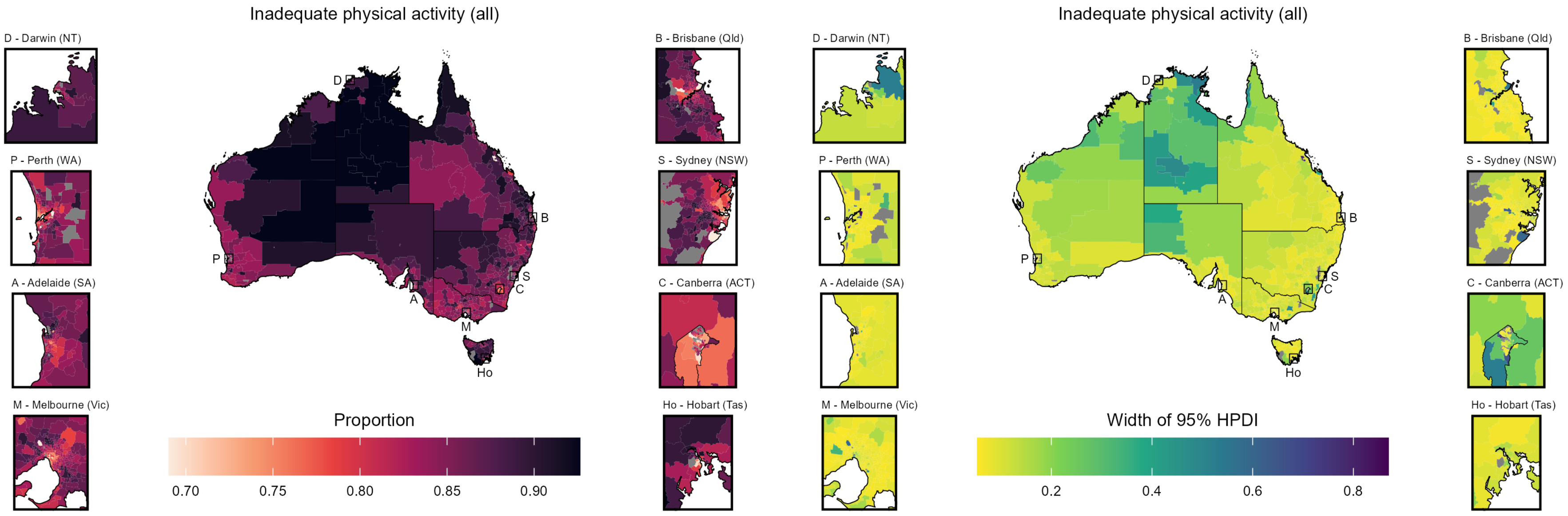

(a) (b)

Figure 35: See caption for Fig. 14.

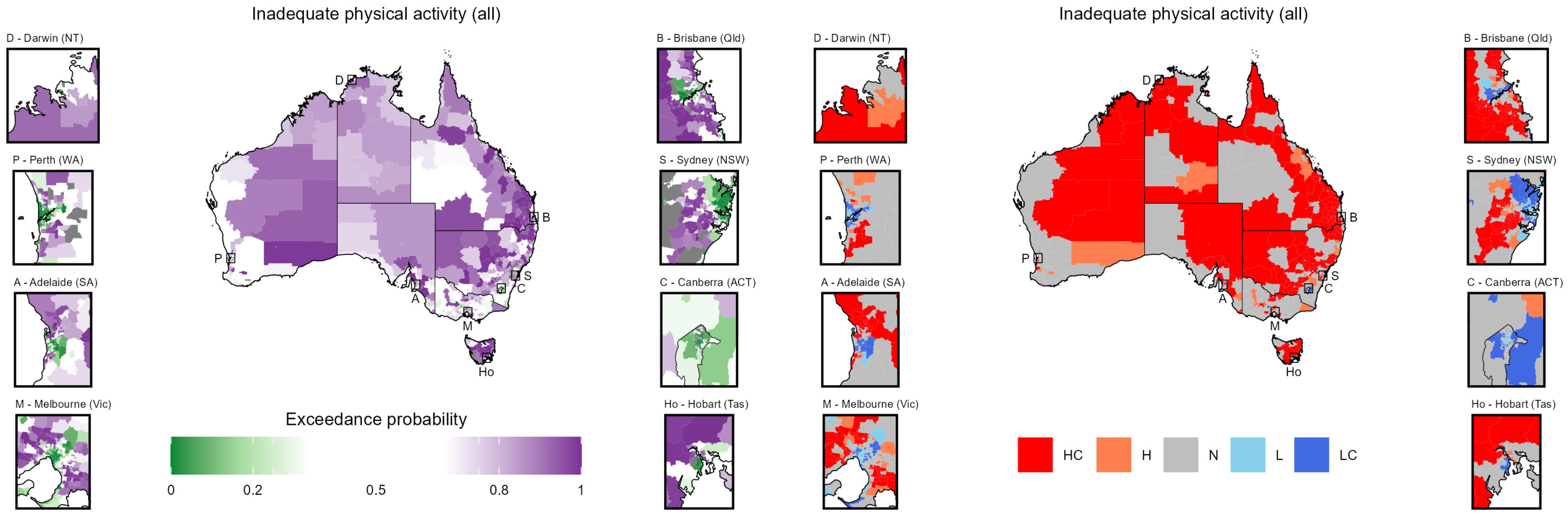

(a) (b)

Figure 36: See caption for Fig. 15.

I Abbreviations

ABS: Australian Bureau of Statistics; ACA: Australian Cancer Atlas; ACT: Australian Capital Territory; AIHW: Australian Institute of Health and Welfare; ALC: Area linear comparison; ASGS: Australian Statistical Geography Standard; BMI: Body Mass Index; DH: Demographic-health; DOH: Department of Health; EP: Exceedance probability; HPDI: Highest posterior density interval; ICAR: Intrinsic conditional autoregressive; IID: Independent and identically distributed; IOP: Interval overlap probability; IQR: Interquartile range; IRSD: Index of Relative Socio-Economic Disadvantage; MARB: Mean absolute relative bias; MCMC: Markov Chain monte carlo; MIOP: Mean interval overlap probability; MrP: Multilevel regression and poststratification; MRRMSE: Mean relative root mean squared error; NHMRC: National Health and Medical Research Council; NHS: National Health Survey; NORF: Non-outcome risk factor; NSW: New South Wales; NT: Northern Territory; PC: Principal component; PHN: Primary health network; QLD: Queensland; RR: Rate ratio; SA: South Australia; SA2: Statistical area level 2; SA3: Statistical area level 3; SA4: Statistical area level 4; SEIFA: Socio-Economic Indexes for Areas; SES: Socioeconomic status; SHAA: Social Health Atlases of Australia; SR: Smoothing ratio; TAS: Tasmania; TSLN: Two-stage logistic-normal; VIC: Victoria; WA: Western Australia

References

[1] Ministry of Health. New south wales population health surveys, 2023. URL https://www.health.nsw.gov.au/surveys/Pages/default.aspx.

[2] Ministry of Health. Healthstats nsw, 2021. URL https://www.healthstats.nsw.gov.au/#/home.

[3] Victorian Agency for Health Information. Victorian population health survey 2017, 2017. URL https://www.health.vic.gov.au/population-health-systems/victorian-population-health-survey-2017.

[4] Australian Bureau of Statistics. 1270.0.55.005 - australian statistical geography standard (asgs): Volume 5 - remoteness structure, july 2016, 2016. URL https://www.abs.gov.au/ausstats/abs@.nsf/Latestproducts/1270.0.55.005Main%20Features15July%202016?opendocument&tabname=Summary&prodno=1270.0.55.005&issue=July%202016&num=&view=.

[5] Queensland Health. About the preventive health survey and queensland survey analytic system, 2021. URL https://www.health.qld.gov.au/research-reports/population-health/preventive-health-surveys/about.

[6] A Radomiljac, C Davies, and T Landrigan. Health and wellbeing of adults in western australia 2018, overview and trends. Report, Department of Health, Western Australia, 2019.

[7] South Australia Health. South australian population health survey, 2023. URL https://www.sahealth.sa.gov.au/wps/wcm/connect/public+content/sa+health+internet/about+us/health+statistics/sa+population+health+survey.

[8] The Social Research Centre. Northern territory population health survey, 2022. URL https://srcentre.com.au/our-research/northern-territory-population-health-survey.

[9] Australian Bureau of Statistics. Microdata: National Health Survey 2017-18 [DataLab], 2017.

[10] Australian Bureau of Statistics. National aboriginal and torres strait islander health survey, 2019. URL https://www.abs.gov.au/statistics/people/aboriginal-and-torres-strait-islander-peoples/national-aboriginal-and-torres-strait-islander-health-survey/2018-19#key-statistics.

[11] Department of Health Tasmania. Report on the tasmanian population health survey 2019. Report, Department of Health Tasmania,, 2020.

[12] ACT Health. Act general health survey 2019 summary results. Report, ACT Government, 2021.

[13] Australian Institute of Health and Welfare. National drug strategy household survey 2019. Report, AIHW, Australian Government, Canberra, 2020.

[14] Australian Bureau of Statistics. Results from the 2018-19 national aboriginal and torres strait islander health survey (natsihs). Report, Australian Bureau of Statistics,, 2019. URL https://apo.org.au/sites/default/files/resource-files/2020-06/apo-nid306475.pdf.

[15] D. C. Whiteman, P. M. Webb, A. C. Green, R. E. Neale, L. Fritschi, C. J. Bain, D. M. Parkin, L. F. Wilson, C. M. Olsen, C. M. Nagle, N. Pandeya, S. J. Jordan, A. Antonsson, B. J. Kendall, M. C. B. Hughes, T. I. Ibiebele, K. Miura, S. Peters, and R. N. Carey. Cancers in australia in 2010 attributable to modifiable factors: introduction and overview. *Australian and New Zealand Journal of Public Health*, 39(5):403–407, 2015. doi:10.1111/1753-6405.12471.

[16] Australian Institute of Health Welfare. Australian burden of disease study: impact and causes of illness and death in australia 2015. Report, Australian Institute of Health Welfare, 2019. URL https://www.aihw.gov.au/reports/burden-of-disease/burden-disease-study-illness-death-2015.

[17] Public Health Information Development Unit. Social health atlases of australia, 2018. URL https://phidu.torrens.edu.au/social-health-atlases.

[18] Hilary A. Tindle, Melanie Stevenson Duncan, Robert A. Greevy, Ramachandran S. Vasan, Suman Kundu, Pierre P. Massion, and Matthew S. Freiberg. Lifetime smoking history and risk of lung cancer: Results from the framingham heart study. *Journal of the National Cancer Institute*, 110(11):1201–1207, 2018. doi:10.1093/jnci/djy041.

[19] National Health and Medical Research Council. Australian guidelines to reduce health risks from drinking alcohol. Report, National Health and Medical Research Council, 2020.

[20] World Cancer Research Fund and American Institute for Cancer Research. Recommendations and public health and policy implications. Report, World Cancer Research Fund/American Institute for Cancer Research,, 2018. URL dietandcancerreport.org.

[21] Cancer Australia. Lifestyle risk factors and the primary prevention of cancer, 2015.

[22] U.S. Department of Agriculture, U.S. Department of Health and Human Services. Dietary guidelines for Americans, 2020-2025. Report, U.S. Department of Agriculture, U.S. Department of Health and Human Services, 2020. URL https://www.dietaryguidelines.gov/resources/2020-2025-dietary-guidelines-online-materials.

[23] Cancer Council Australia. Limit alcohol, 2023. URL https://www.cancer.org.au/cancer-information/causes-and-prevention/diet-and-exercise/limit-alcohol.

[24] World Cancer Research Fund and American Institute for Cancer Research. Exposures, risk factors and cancer, 2018. URL https://www.wcrf.org/diet-and-cancer/exposures/.

[25] Australian Bureau of Statistics. 4363.0 - national health survey: Users' guide, 2017-18, 2017.

[26] National Health and Medical Research Council. Australian dietary guidelines. Report, National Health and Medical Research Council, 2013.

[27] Australian Bureau of Statistics. National Health Survey: First results methodology, 2018. URL https://www.abs.gov.au/methodologies/national-health-survey-first-results-methodology/2017-18.

[28] James Hogg, Jessica Cameron, Susanna Cramb, Peter Baade, and Kerrie Mengersen. A two-stage bayesian small area estimation method for proportions. *arXiv preprint arXiv:2306.11302*, 2003.

[29] World Health Organization. Obesity and overweight, 2020. URL https://www.who.int/news-room/fact-sheets/detail/healthy-diet.

[30] National Health and Medical Research Council. Clinical practice guidelines for the management of overweight and obesity in adults, adolescents and children in Australia. Report, National Health and Medical Research Council, 2013.

[31] Cancer Council Australia. Maintain a healthy weight, 2023. URL https://www.cancer.org.au/cancer-information/causes-and-prevention/diet-and-exercise/maintain-a-healthy-weight.

[32] X. Zhang, J. B. Holt, S. Yun, H. Lu, K. J. Greenlund, and J. B. Croft. Validation of multilevel regression and poststratification methodology for small area estimation of health indicators from the behavioral risk factor surveillance system. *American Journal of Epidemiology*, 182(2):127–37, 2015. ISSN 0002-9262 (Print) 0002-9262. doi:10.1093/aje/kwv002.

[33] Harrison Quick, Dina Terloyeva, Yaxin Wu, Kari Moore, and Ana V. Diez Roux. Trends in tract-level prevalence of obesity in philadelphia by race-ethnicity, space, and time. *Epidemiology*, 31(1), 2020. ISSN 1044-3983.

[34] Centre for Disease Control. PLACES: Local data for better health, 2021. URL https://www.cdc.gov/places.

[35] M. W. Sullivan, F. T. Camacho, A. M. Mills, and S. C. Modesitt. Missing information in statewide and national cancer databases: Correlation with health risk factors, geographic disparities, and outcomes. *Gynecologic Oncology*, 152(1):119–126, 2019. doi:10.1016/j.ygyno.2018.10.029.

[36] Melissa D. Olfert, Makenzie L. Barr, Camille M. Charlier, Oluremi A. Famodu, Wenjun Zhou, Anne E. Mathews, Carol Byrd-Bredbenner, and Sarah E. Colby. Self-reported vs. measured height, weight, and bmi in young adults. *International Journal of Environmental Research and Public Health*, 15(10):2216, 2018. doi:10.3390/ijerph15102216.

[37] Department of Health. Physical activity and exercise guidelines for all australians, 2014. URL https://www.health.gov.au/resources/collections/collection-of-physical-activity-and-sedentary-behaviour-guidelines-for-all-ages.

[38] World Health Organization. Physical activity, 2020. URL https://www.who.int/news-room/fact-sheets/detail/physical-activity.

[39] Wendy J Brown, Adrian E Bauman, Fiona C Bull, and Nicola W Burton. Development of evidence-based physical activity recommendations for adults (18-64 years). Report, Department of Health, Australian Government, 2012.

[40] Anne McTiernan, Christine M. Friedenreich, Peter T. Katzmarzyk, Kenneth E. Powell, Richard Macko, David Buchner, Linda S. Pescatello, Bonny Bloodgood, Bethany Tennant, Alison Vaux-Bjerke, Stephanie M. George, Richard P. Troiano, Katrina L. Piercy, and Committee Physical Activity Guidelines Advisory. Physical activity in cancer prevention and survival: A systematic review. *Medicine and Science in Sports and Exercise*, 51(6): 1252–1261, 2019. ISSN 1530-0315 0195-9131. doi:10.1249/MSS.0000000000001937.

[41] Australian Institute of Health and Welfare. Physical activity, 2022. URL https://www.aihw.gov.au/reports-data/behaviours-risk-factors/physical-activity/overview.

[42] Paul A. Parker, Ryan Janicki, and Scott H. Holan. Unit level modeling of survey data for small area estimation under informative sampling: A comprehensive overview with extensions. *arXiv preprint arXiv:1908.10488*, 2019.

[43] Australian Bureau of Statistics. Technical paper: Socio-economic indexes for areas (SEIFA), 2016.

[44] Yair Ghitza and Andrew Gelman. Deep interactions with mrp: Election turnout and voting patterns among small electoral subgroups. *American Journal of Political Science*, 57(3):762–776, 2013. ISSN 0092-5853. doi:https://doi.org/10.1111/ajps.12004. URL https://doi.org/10.1111/ajps.12004.

[45] David A Binder. On the variances of asymptotically normal estimators from complex surveys. *International Statistical Review*, pages 279–292, 1983. ISSN 0306-7734.

[46] Terrance D Savitsky and Daniell Toth. Bayesian estimation under informative sampling. *Electronic Journal of Statistics*, 10(1):1677–1708, 2016. ISSN 1935-7524.

[47] Raymond J. Carroll, David Ruppert, Leonard A. Stefanski, and Ciprian M. Crainiceanu. *Measurement Error in Nonlinear Models : A Modern Perspective, Second Edition*. CRC Press LLC, London, UNITED KINGDOM, 2006. ISBN 9781420010138. URL http://ebookcentral.proquest.com/lib/qut/detail.action?docID=274076.

[48] Connor Donegan, Yongwan Chun, and Daniel A. Griffith. Modeling community health with areal data: Bayesian inference with survey standard errors and spatial structure. *International Journal of Environmental Research and Public Health*, 18(13):6856, 2021. ISSN 1660-4601. URL https://www.mdpi.com/1660-4601/18/13/6856.

[49] Hong Xia and Bradley P Carlin. Spatio-temporal models with errors in covariates: mapping ohio lung cancer mortality. *Statistics in medicine*, 17(18):2025–2043, 1998. ISSN 0277-6715.

[50] L Bernadinelli, Cristian Pascutto, NG Best, and WR Gilks. Disease mapping with errors in covariates. *Statistics in medicine*, 16(7):741–752, 1997. ISSN 0277-6715.

[51] Hee Cheol Chung and Gauri Sankar Datta. Bayesian hierarchical spatial models for small area estimation. Report, Center for Statistical Research and Methodology, 2020.

[52] Y. Vandendijck, C. Faes, R. S. Kirby, A. Lawson, and N. Hens. Model-based inference for small area estimation with sampling weights. *Spatial Statistics*, 18(1):455–473, 2016. doi:10.1016/j.spasta.2016.09.004. URL https://www.scopus.com/inward/record.uri?eid=2-s2.0-85000838110&doi=10.1016%2fj.spasta.2016.09.004&partnerID=40&md5=ebcb15c3b399bab7fb52f71583efed64.

[53] Peter A. Gao and Jon Wakefield. Smoothed model-assisted small area estimation of proportions. *Canadian Journal of Statistics*, 2023. doi:https://doi.org/10.1002/cjs.11787.

[54] John Paige, Geir-Arne Fuglstad, Andrea Riebler, and Jon Wakefield. Design-and model-based approaches to small-area estimation in a low-and middle-income country context: comparisons and recommendations. *Journal of Survey Statistics and Methodology*, 10(1):50–80, 2022. ISSN 2325-0984.

[55] Julian Besag, Jeremy York, and Annie Mollié. Bayesian image restoration, with two applications in spatial statistics. *Annals of the Institute of Statistical Mathematics*, 43(1):1–20, 1991. ISSN 1572-9052. doi:10.1007/BF00116466. URL https://doi.org/10.1007/BF00116466.

[56] V. Gomez-Rubio, Nicky Best, Sylvia Richardson, Guangquan Li, and Philip Clarke. Bayesian statistics small area estimation. Report, Office for National Statistics, 01/01 2008.

[57] Andrea Riebler, Sigrunn H. Sørbye, Daniel Simpson, and Håvard Rue. An intuitive bayesian spatial model for disease mapping that accounts for scaling. 2016. doi:arXiv:1601.01180.

[58] Susanna Cramb, Earl Duncan, Peter Baade, and Kerrie L. Mengersen. *A Comparison of Bayesian Spatial Models for Cancer Incidence at a Small Area Level: Theory and Performance*, pages 245–274. Springer International Publishing, New York City, USA, 2020. ISBN 978-3-030-42553-1. doi:10.1007/978-3-030-42553-1_10. URL https://doi.org/10.1007/978-3-030-42553-1_10.

[59] Leyla Mohadjer, JNK Rao, Benmei Liu, Tom Krenzke, and Wendy Van de Kerckhove. Hierarchical bayes small area estimates of adult literacy using unmatched sampling and linking models. In *Proceedings of the Survey Research Methods Section*, pages 3203–3210.

[60] Sudipto Banerjee, Bradley P. Carlin, and Alan E. Gelfand. *Hierarchical modeling and analysis for spatial data*. Monographs on statistics and applied probability ; 135. CRC Press, Taylor and Francis Group, Boca Raton, second edition. edition, 2015. ISBN 9781439819180.

[61] M. Morris, K. Wheeler-Martin, D. Simpson, S. J. Mooney, A. Gelman, and C. DiMaggio. Bayesian hierarchical spatial models: Implementing the besag york mollié model in stan. *Spatial and Spatio-temporal Epidemiology*, (1877-5853 (Electronic)), 2019. doi:10.1016/j.sste.2019.100301.

[62] Kirk M. Wolter. *Introduction to Variance Estimation*. Springer, New York, NY, 2007. doi:https://doi.org/10.1007/978-0-387-35099-8.

[63] J.N.K. Rao and Isabel Molina. *Small Area Estimation*. Wiley Series in Survey Methodology, Hoboken, New Jersey, 2nd edition, 2015.

[64] M. Hidiroglou and Y. You. Comparison of unit level and area level small area estimators. *Survey Methodology*, 42 (1):41–61, 2016.

[65] S. Das, J. van den Brakel, H.J. Boonstra, and S. Haslett. Multilevel time series modelling of antenatal care coverage in bangladesh at disaggregated administrative levels. *Survey Methodology*, 48(2), 2022. URL http://www.statcan.gc.ca/pub/12-001-x/2022002/article/00010-eng.htm.

[66] Peter A. Gao and Jon Wakefield. A spatial variance-smoothing area level model for small area estimation of demographic rates. *arXiv preprint arXiv:2209.02602*, 2022. doi:arXiv:2209.02602.

[67] J. Hajek. Comment on "an essay on the logical foundations of survey sampling, part one". *The Foundations of Survey Sampling*, 1971.

[68] Australian Bureau of Statistics. 4710.0 - housing and infrastructure in aboriginal and torres strait islander communities, australia, 2006, 2007. URL https://www.abs.gov.au/ausstats/abs@.nsf/mf/4710.0.

[69] Australian Institute of Health and Welfare. Rural and remote health, 2022. URL https://www.aihw.gov.au/reports/rural-remote-australians/rural-and-remote-health.

[70] Australian Bureau of Statistics. Census of population and housing: Census dictionary, 2016, 2016. URL https://www.abs.gov.au/ausstats/abs@.nsf/Lookup/2901.0Main%20Features12016.

[71] Marco Gramatica, Peter Congdon, and Silvia Liverani. Bayesian modelling for spatially misaligned health areal data: A multiple membership approach. *Journal of the Royal Statistical Society: Series C (Applied Statistics)*, 70(3):645–666, 2021. ISSN 0035-9254. doi:https://doi.org/10.1111/rssc.12480. URL https://doi.org/10.1111/rssc.12480.

[72] Peter Congdon. Assessing persistence in spatial clustering of disease, with an application to drug related deaths in scottish neighbourhoods. *Epidemiology Biostatistics and Public Health*, 2020.

[73] L. Anselin. Local indicators of spatial association - LISA. *Geographical Analysis*, 27(2):93–115, 1995. ISSN 0016-7363. doi:10.1111/j.1538-4632.1995.tb00338.x.

[74] A. Vehtari, A. Gelman, and J. Gabry. Practical bayesian model evaluation using leave-one-out cross-validation and waic. *Statistics and Computing*, 27(5):1413–1432, 2017. doi:10.1007/s11222-016-9696-4. URL https://www.scopus.com/inward/record.uri?eid=2-s2.0-85026299835&doi=10.1007%2fs11222-016-9696-4&partnerID=40&md5=d4d48dc9b435386f7e4be2c5cb580f4b.

[75] Sumonkanti Das, Bernard Baffour, Alice Richardson, Susanna Cramb, and Stephen Haslett. Daily smoking prevalence for small domains in Australia. *Research Square preprint*, 2023. doi:10.21203/rs.3.rs-2829471/v1. URL https://www.researchsquare.com/article/rs-2829471/v1.

[76] S. E. Collins. Associations between socioeconomic factors and alcohol outcomes. *Alcohol Research*, 38(2168-3492 (Print)):83–94.

[77] K. A. E. Patterson, V. Cleland, A. Venn, L. Blizzard, and S. Gall. A cross-sectional study of geographic differences in health risk factors among young australian adults: The role of socioeconomic position. *BMC Public Health*, 14(1), 2014. doi:10.1186/1471-2458-14-1278. URL https://www.scopus.com/inward/record.uri?eid=2-s2.0-84924288776&doi=10.1186%2f1471-2458-14-1278&partnerID=40&md5=775e49b9ba2c4478ae77d0e0c641ce77.
